# Measurements of electroweak $$Wjj $$ production and constraints on anomalous gauge couplings with the ATLAS detector

**DOI:** 10.1140/epjc/s10052-017-5007-2

**Published:** 2017-07-17

**Authors:** M. Aaboud, G. Aad, B. Abbott, J. Abdallah, O. Abdinov, B. Abeloos, S. H. Abidi, O. S. AbouZeid, N. L. Abraham, H. Abramowicz, H. Abreu, R. Abreu, Y. Abulaiti, B. S. Acharya, S. Adachi, L. Adamczyk, D. L. Adams, J. Adelman, M. Adersberger, T. Adye, A. A. Affolder, T. Agatonovic-Jovin, C. Agheorghiesei, J. A. Aguilar-Saavedra, S. P. Ahlen, F. Ahmadov, G. Aielli, S. Akatsuka, H. Akerstedt, T. P. A. Åkesson, A. V. Akimov, G. L. Alberghi, J. Albert, M. J. Alconada Verzini, M. Aleksa, I. N. Aleksandrov, C. Alexa, G. Alexander, T. Alexopoulos, M. Alhroob, B. Ali, M. Aliev, G. Alimonti, J. Alison, S. P. Alkire, B. M. M. Allbrooke, B. W. Allen, P. P. Allport, A. Aloisio, A. Alonso, F. Alonso, C. Alpigiani, A. A. Alshehri, M. Alstaty, B. Alvarez Gonzalez, D. Álvarez Piqueras, M. G. Alviggi, B. T. Amadio, Y. Amaral Coutinho, C. Amelung, D. Amidei, S. P. Amor Dos Santos, A. Amorim, S. Amoroso, G. Amundsen, C. Anastopoulos, L. S. Ancu, N. Andari, T. Andeen, C. F. Anders, J. K. Anders, K. J. Anderson, A. Andreazza, V. Andrei, S. Angelidakis, I. Angelozzi, A. Angerami, F. Anghinolfi, A. V. Anisenkov, N. Anjos, A. Annovi, C. Antel, M. Antonelli, A. Antonov, D. J. Antrim, F. Anulli, M. Aoki, L. Aperio Bella, G. Arabidze, Y. Arai, J. P. Araque, V. Araujo Ferraz, A. T. H. Arce, R. E. Ardell, F. A. Arduh, J.-F. Arguin, S. Argyropoulos, M. Arik, A. J. Armbruster, L. J. Armitage, O. Arnaez, H. Arnold, M. Arratia, O. Arslan, A. Artamonov, G. Artoni, S. Artz, S. Asai, N. Asbah, A. Ashkenazi, L. Asquith, K. Assamagan, R. Astalos, M. Atkinson, N. B. Atlay, K. Augsten, G. Avolio, B. Axen, M. K. Ayoub, G. Azuelos, A. E. Baas, M. J. Baca, H. Bachacou, K. Bachas, M. Backes, M. Backhaus, P. Bagiacchi, P. Bagnaia, J. T. Baines, M. Bajic, O. K. Baker, E. M. Baldin, P. Balek, T. Balestri, F. Balli, W. K. Balunas, E. Banas, Sw. Banerjee, A. A. E. Bannoura, L. Barak, E. L. Barberio, D. Barberis, M. Barbero, T. Barillari, M-S Barisits, T. Barklow, N. Barlow, S. L. Barnes, B. M. Barnett, R. M. Barnett, Z. Barnovska-Blenessy, A. Baroncelli, G. Barone, A. J. Barr, L. Barranco Navarro, F. Barreiro, J. Barreiro Guimarães da Costa, R. Bartoldus, A. E. Barton, P. Bartos, A. Basalaev, A. Bassalat, R. L. Bates, S. J. Batista, J. R. Batley, M. Battaglia, M. Bauce, F. Bauer, H. S. Bawa, J. B. Beacham, M. D. Beattie, T. Beau, P. H. Beauchemin, P. Bechtle, H. P. Beck, K. Becker, M. Becker, M. Beckingham, C. Becot, A. J. Beddall, A. Beddall, V. A. Bednyakov, M. Bedognetti, C. P. Bee, T. A. Beermann, M. Begalli, M. Begel, J. K. Behr, A. S. Bell, G. Bella, L. Bellagamba, A. Bellerive, M. Bellomo, K. Belotskiy, O. Beltramello, N. L. Belyaev, O. Benary, D. Benchekroun, M. Bender, K. Bendtz, N. Benekos, Y. Benhammou, E. Benhar Noccioli, J. Benitez, D. P. Benjamin, M. Benoit, J. R. Bensinger, S. Bentvelsen, L. Beresford, M. Beretta, D. Berge, E. Bergeaas Kuutmann, N. Berger, J. Beringer, S. Berlendis, N. R. Bernard, G. Bernardi, C. Bernius, F. U. Bernlochner, T. Berry, P. Berta, C. Bertella, G. Bertoli, F. Bertolucci, I. A. Bertram, C. Bertsche, D. Bertsche, G. J. Besjes, O. Bessidskaia Bylund, M. Bessner, N. Besson, C. Betancourt, A. Bethani, S. Bethke, A. J. Bevan, R. M. Bianchi, M. Bianco, O. Biebel, D. Biedermann, R. Bielski, N. V. Biesuz, M. Biglietti, J. Bilbao De Mendizabal, T. R. V. Billoud, H. Bilokon, M. Bindi, A. Bingul, C. Bini, S. Biondi, T. Bisanz, C. Bittrich, D. M. Bjergaard, C. W. Black, J. E. Black, K. M. Black, D. Blackburn, R. E. Blair, T. Blazek, I. Bloch, C. Blocker, A. Blue, W. Blum, U. Blumenschein, S. Blunier, G. J. Bobbink, V. S. Bobrovnikov, S. S. Bocchetta, A. Bocci, C. Bock, M. Boehler, D. Boerner, D. Bogavac, A. G. Bogdanchikov, C. Bohm, V. Boisvert, P. Bokan, T. Bold, A. S. Boldyrev, M. Bomben, M. Bona, M. Boonekamp, A. Borisov, G. Borissov, J. Bortfeldt, D. Bortoletto, V. Bortolotto, K. Bos, D. Boscherini, M. Bosman, J. D. Bossio Sola, J. Boudreau, J. Bouffard, E. V. Bouhova-Thacker, D. Boumediene, C. Bourdarios, S. K. Boutle, A. Boveia, J. Boyd, I. R. Boyko, J. Bracinik, A. Brandt, G. Brandt, O. Brandt, U. Bratzler, B. Brau, J. E. Brau, W. D. Breaden Madden, K. Brendlinger, A. J. Brennan, L. Brenner, R. Brenner, S. Bressler, D. L. Briglin, T. M. Bristow, D. Britton, D. Britzger, F. M. Brochu, I. Brock, R. Brock, G. Brooijmans, T. Brooks, W. K. Brooks, J. Brosamer, E. Brost, J. H Broughton, P. A. Bruckman de Renstrom, D. Bruncko, A. Bruni, G. Bruni, L. S. Bruni, BH Brunt, M. Bruschi, N. Bruscino, P. Bryant, L. Bryngemark, T. Buanes, Q. Buat, P. Buchholz, A. G. Buckley, I. A. Budagov, F. Buehrer, M. K. Bugge, O. Bulekov, D. Bullock, H. Burckhart, S. Burdin, C. D. Burgard, A. M. Burger, B. Burghgrave, K. Burka, S. Burke, I. Burmeister, J. T. P. Burr, E. Busato, D. Büscher, V. Büscher, P. Bussey, J. M. Butler, C. M. Buttar, J. M. Butterworth, P. Butti, W. Buttinger, A. Buzatu, A. R. Buzykaev, S. Cabrera Urbán, D. Caforio, V. M. Cairo, O. Cakir, N. Calace, P. Calafiura, A. Calandri, G. Calderini, P. Calfayan, G. Callea, L. P. Caloba, S. Calvente Lopez, D. Calvet, S. Calvet, T. P. Calvet, R. Camacho Toro, S. Camarda, P. Camarri, D. Cameron, R. Caminal Armadans, C. Camincher, S. Campana, M. Campanelli, A. Camplani, A. Campoverde, V. Canale, M. Cano Bret, J. Cantero, T. Cao, M. D. M. Capeans Garrido, I. Caprini, M. Caprini, M. Capua, R. M. Carbone, R. Cardarelli, F. Cardillo, I. Carli, T. Carli, G. Carlino, B. T. Carlson, L. Carminati, R. M. D. Carney, S. Caron, E. Carquin, G. D. Carrillo-Montoya, J. Carvalho, D. Casadei, M. P. Casado, M. Casolino, D. W. Casper, R. Castelijn, A. Castelli, V. Castillo Gimenez, N. F. Castro, A. Catinaccio, J. R. Catmore, A. Cattai, J. Caudron, V. Cavaliere, E. Cavallaro, D. Cavalli, M. Cavalli-Sforza, V. Cavasinni, E. Celebi, F. Ceradini, L. Cerda Alberich, A. S. Cerqueira, A. Cerri, L. Cerrito, F. Cerutti, A. Cervelli, S. A. Cetin, A. Chafaq, D. Chakraborty, S. K. Chan, W. S. Chan, Y. L. Chan, P. Chang, J. D. Chapman, D. G. Charlton, A. Chatterjee, C. C. Chau, C. A. Chavez Barajas, S. Che, S. Cheatham, A. Chegwidden, S. Chekanov, S. V. Chekulaev, G. A. Chelkov, M. A. Chelstowska, C. Chen, H. Chen, S. Chen, S. Chen, X. Chen, Y. Chen, H. C. Cheng, H. J. Cheng, Y. Cheng, A. Cheplakov, E. Cheremushkina, R. Cherkaoui El Moursli, V. Chernyatin, E. Cheu, L. Chevalier, V. Chiarella, G. Chiarelli, G. Chiodini, A. S. Chisholm, A. Chitan, Y. H. Chiu, M. V. Chizhov, K. Choi, A. R. Chomont, S. Chouridou, B. K. B. Chow, V. Christodoulou, D. Chromek-Burckhart, M. C. Chu, J. Chudoba, A. J. Chuinard, J. J. Chwastowski, L. Chytka, A. K. Ciftci, D. Cinca, V. Cindro, I. A. Cioara, C. Ciocca, A. Ciocio, F. Cirotto, Z. H. Citron, M. Citterio, M. Ciubancan, A. Clark, B. L. Clark, M. R. Clark, P. J. Clark, R. N. Clarke, C. Clement, Y. Coadou, M. Cobal, A. Coccaro, J. Cochran, L. Colasurdo, B. Cole, A. P. Colijn, J. Collot, T. Colombo, P. Conde Muiño, E. Coniavitis, S. H. Connell, I. A. Connelly, V. Consorti, S. Constantinescu, G. Conti, F. Conventi, M. Cooke, B. D. Cooper, A. M. Cooper-Sarkar, F. Cormier, K. J. R. Cormier, T. Cornelissen, M. Corradi, F. Corriveau, A. Cortes-Gonzalez, G. Cortiana, G. Costa, M. J. Costa, D. Costanzo, G. Cottin, G. Cowan, B. E. Cox, K. Cranmer, S. J. Crawley, R. A. Creager, G. Cree, S. Crépé-Renaudin, F. Crescioli, W. A. Cribbs, M. Crispin Ortuzar, M. Cristinziani, V. Croft, G. Crosetti, A. Cueto, T. Cuhadar Donszelmann, J. Cummings, M. Curatolo, J. Cúth, H. Czirr, P. Czodrowski, G. D’amen, S. D’Auria, M. D’Onofrio, M. J. Da Cunha Sargedas De Sousa, C. Da Via, W. Dabrowski, T. Dado, T. Dai, O. Dale, F. Dallaire, C. Dallapiccola, M. Dam, J. R. Dandoy, N. P. Dang, A. C. Daniells, N. S. Dann, M. Danninger, M. Dano Hoffmann, V. Dao, G. Darbo, S. Darmora, J. Dassoulas, A. Dattagupta, T. Daubney, W. Davey, C. David, T. Davidek, M. Davies, P. Davison, E. Dawe, I. Dawson, K. De, R. de Asmundis, A. De Benedetti, S. De Castro, S. De Cecco, N. De Groot, P. de Jong, H. De la Torre, F. De Lorenzi, A. De Maria, D. De Pedis, A. De Salvo, U. De Sanctis, A. De Santo, K. De Vasconcelos Corga, J. B. De Vivie De Regie, W. J. Dearnaley, R. Debbe, C. Debenedetti, D. V. Dedovich, N. Dehghanian, I. Deigaard, M. Del Gaudio, J. Del Peso, T. Del Prete, D. Delgove, F. Deliot, C. M. Delitzsch, A. Dell’Acqua, L. Dell’Asta, M. Dell’Orso, M. Della Pietra, D. della Volpe, M. Delmastro, P. A. Delsart, D. A. DeMarco, S. Demers, M. Demichev, A. Demilly, S. P. Denisov, D. Denysiuk, D. Derendarz, J. E. Derkaoui, F. Derue, P. Dervan, K. Desch, C. Deterre, K. Dette, P. O. Deviveiros, A. Dewhurst, S. Dhaliwal, A. Di Ciaccio, L. Di Ciaccio, W. K. Di Clemente, C. Di Donato, A. Di Girolamo, B. Di Girolamo, B. Di Micco, R. Di Nardo, K. F. Di Petrillo, A. Di Simone, R. Di Sipio, D. Di Valentino, C. Diaconu, M. Diamond, F. A. Dias, M. A. Diaz, E. B. Diehl, J. Dietrich, S. Díez Cornell, A. Dimitrievska, J. Dingfelder, P. Dita, S. Dita, F. Dittus, F. Djama, T. Djobava, J. I. Djuvsland, M. A. B. do Vale, D. Dobos, M. Dobre, C. Doglioni, J. Dolejsi, Z. Dolezal, M. Donadelli, S. Donati, P. Dondero, J. Donini, J. Dopke, A. Doria, M. T. Dova, A. T. Doyle, E. Drechsler, M. Dris, Y. Du, J. Duarte-Campderros, E. Duchovni, G. Duckeck, O. A. Ducu, D. Duda, A. Dudarev, A. Chr. Dudder, E. M. Duffield, L. Duflot, M. Dührssen, M. Dumancic, A. E. Dumitriu, A. K. Duncan, M. Dunford, H. Duran Yildiz, M. Düren, A. Durglishvili, D. Duschinger, B. Dutta, M. Dyndal, C. Eckardt, K. M. Ecker, R. C. Edgar, T. Eifert, G. Eigen, K. Einsweiler, T. Ekelof, M. El Kacimi, V. Ellajosyula, M. Ellert, S. Elles, F. Ellinghaus, A. A. Elliot, N. Ellis, J. Elmsheuser, M. Elsing, D. Emeliyanov, Y. Enari, O. C. Endner, J. S. Ennis, J. Erdmann, A. Ereditato, G. Ernis, M. Ernst, S. Errede, E. Ertel, M. Escalier, H. Esch, C. Escobar, B. Esposito, A. I. Etienvre, E. Etzion, H. Evans, A. Ezhilov, F. Fabbri, L. Fabbri, G. Facini, R. M. Fakhrutdinov, S. Falciano, R. J. Falla, J. Faltova, Y. Fang, M. Fanti, A. Farbin, A. Farilla, C. Farina, E. M. Farina, T. Farooque, S. Farrell, S. M. Farrington, P. Farthouat, F. Fassi, P. Fassnacht, D. Fassouliotis, M. Faucci Giannelli, A. Favareto, W. J. Fawcett, L. Fayard, O. L. Fedin, W. Fedorko, S. Feigl, L. Feligioni, C. Feng, E. J. Feng, H. Feng, A. B. Fenyuk, L. Feremenga, P. ernandez Martinez, S. Fernandez Perez, J. Ferrando, A. Ferrari, P. Ferrari, R. Ferrari, D. E. Ferreira de Lima, A. Ferrer, D. Ferrere, C. Ferretti, F. Fiedler, A. Filipčič, M. Filipuzzi, F. Filthaut, M. Fincke-Keeler, K. D. Finelli, M. C. N. Fiolhais, L. Fiorini, A. Fischer, C. Fischer, J. Fischer, W. C. Fisher, N. Flaschel, I. Fleck, P. Fleischmann, R. R. M. Fletcher, T. Flick, B. M. Flierl, L. R. Flores Castillo, M. J. Flowerdew, G. T. Forcolin, A. Formica, A. Forti, A. G. Foster, D. Fournier, H. Fox, S. Fracchia, P. Francavilla, M. Franchini, D. Francis, L. Franconi, M. Franklin, M. Frate, M. Fraternali, D. Freeborn, S. M. Fressard-Batraneanu, B. Freund, D. Froidevaux, J. A. Frost, C. Fukunaga, E. Fullana Torregrosa, T. Fusayasu, J. Fuster, C. Gabaldon, O. Gabizon, A. Gabrielli, A. Gabrielli, G. P. Gach, S. Gadatsch, S. Gadomski, G. Gagliardi, L. G. Gagnon, P. Gagnon, C. Galea, B. Galhardo, E. J. Gallas, B. J. Gallop, P. Gallus, G. Galster, K. K. Gan, S. Ganguly, J. Gao, Y. Gao, Y. S. Gao, F. M. Garay Walls, C. García, J. E. García Navarro, M. Garcia-Sciveres, R. W. Gardner, N. Garelli, V. Garonne, A. Gascon Bravo, K. Gasnikova, C. Gatti, A. Gaudiello, G. Gaudio, I. L. Gavrilenko, C. Gay, G. Gaycken, E. N. Gazis, C. N. P. Gee, M. Geisen, M. P. Geisler, K. Gellerstedt, C. Gemme, M. H. Genest, C. Geng, S. Gentile, C. Gentsos, S. George, D. Gerbaudo, A. Gershon, S. Ghasemi, M. Ghneimat, B. Giacobbe, S. Giagu, P. Giannetti, S. M. Gibson, M. Gignac, M. Gilchriese, D. Gillberg, G. Gilles, D. M. Gingrich, N. Giokaris, M. P. Giordani, F. M. Giorgi, P. F. Giraud, P. Giromini, D. Giugni, F. Giuli, C. Giuliani, M. Giulini, B. K. Gjelsten, S. Gkaitatzis, I. Gkialas, E. L. Gkougkousis, L. K. Gladilin, C. Glasman, J. Glatzer, P. C. F. Glaysher, A. Glazov, M. Goblirsch-Kolb, J. Godlewski, S. Goldfarb, T. Golling, D. Golubkov, A. Gomes, R. Gonçalo, R. Goncalves Gama, J. Goncalves Pinto Firmino Da Costa, G. Gonella, L. Gonella, A. Gongadze, S. González de la Hoz, S. Gonzalez-Sevilla, L. Goossens, P. A. Gorbounov, H. A. Gordon, I. Gorelov, B. Gorini, E. Gorini, A. Gorišek, A. T. Goshaw, C. Gössling, M. I. Gostkin, C. R. Goudet, D. Goujdami, A. G. Goussiou, N. Govender, E. Gozani, L. Graber, I. Grabowska-Bold, P. O. J. Gradin, J. Gramling, E. Gramstad, S. Grancagnolo, V. Gratchev, P. M. Gravila, H. M. Gray, Z. D. Greenwood, C. Grefe, K. Gregersen, I. M. Gregor, P. Grenier, K. Grevtsov, J. Griffiths, A. A. Grillo, K. Grimm, S. Grinstein, Ph. Gris, J.-F. Grivaz, S. Groh, E. Gross, J. Grosse-Knetter, G. C. Grossi, Z. J. Grout, L. Guan, W. Guan, J. Guenther, F. Guescini, D. Guest, O. Gueta, B. Gui, E. Guido, T. Guillemin, S. Guindon, U. Gul, C. Gumpert, J. Guo, W. Guo, Y. Guo, R. Gupta, S. Gupta, G. Gustavino, P. Gutierrez, N. G. Gutierrez Ortiz, C. Gutschow, C. Guyot, M. P. Guzik, C. Gwenlan, C. B. Gwilliam, A. Haas, C. Haber, H. K. Hadavand, A. Hadef, S. Hageböck, M. Hagihara, H. Hakobyan, M. Haleem, J. Haley, G. Halladjian, G. D. Hallewell, K. Hamacher, P. Hamal, K. Hamano, A. Hamilton, G. N. Hamity, P. G. Hamnett, L. Han, S. Han, K. Hanagaki, K. Hanawa, M. Hance, B. Haney, P. Hanke, R. Hanna, J. B. Hansen, J. D. Hansen, M. C. Hansen, P. H. Hansen, K. Hara, A. S. Hard, T. Harenberg, F. Hariri, S. Harkusha, R. D. Harrington, P. F. Harrison, F. Hartjes, N. M. Hartmann, M. Hasegawa, Y. Hasegawa, A. Hasib, S. Hassani, S. Haug, R. Hauser, L. Hauswald, L. B. Havener, M. Havranek, C. M. Hawkes, R. J. Hawkings, D. Hayakawa, D. Hayden, C. P. Hays, J. M. Hays, H. S. Hayward, S. J. Haywood, S. J. Head, T. Heck, V. Hedberg, L. Heelan, K. K. Heidegger, S. Heim, T. Heim, B. Heinemann, J. J. Heinrich, L. Heinrich, C. Heinz, J. Hejbal, L. Helary, A. Held, S. Hellman, C. Helsens, J. Henderson, R. C. W. Henderson, Y. Heng, S. Henkelmann, A. M. Henriques Correia, S. Henrot-Versille, G. H. Herbert, H. Herde, V. Herget, Y. Hernández Jiménez, G. Herten, R. Hertenberger, L. Hervas, T. C. Herwig, G. G. Hesketh, N. P. Hessey, J. W. Hetherly, S. Higashino, E. Higón-Rodriguez, E. Hill, J. C. Hill, K. H. Hiller, S. J. Hillier, I. Hinchliffe, M. Hirose, D. Hirschbuehl, B. Hiti, O. Hladik, X. Hoad, J. Hobbs, N. Hod, M. C. Hodgkinson, P. Hodgson, A. Hoecker, M. R. Hoeferkamp, F. Hoenig, D. Hohn, T. R. Holmes, M. Homann, S. Honda, T. Honda, T. M. Hong, B. H. Hooberman, W. H. Hopkins, Y. Horii, A. J. Horton, J.-Y. Hostachy, S. Hou, A. Hoummada, J. Howarth, J. Hoya, M. Hrabovsky, I. Hristova, J. Hrivnac, T. Hryn’ova, A. Hrynevich, P. J. Hsu, S.-C. Hsu, Q. Hu, S. Hu, Y. Huang, Z. Hubacek, F. Hubaut, F. Huegging, T. B. Huffman, E. W. Hughes, G. Hughes, M. Huhtinen, P. Huo, N. Huseynov, J. Huston, J. Huth, G. Iacobucci, G. Iakovidis, I. Ibragimov, L. Iconomidou-Fayard, P. Iengo, O. Igonkina, T. Iizawa, Y. Ikegami, M. Ikeno, Y. Ilchenko, D. Iliadis, N. Ilic, G. Introzzi, P. Ioannou, M. Iodice, K. Iordanidou, V. Ippolito, N. Ishijima, M. Ishino, M. Ishitsuka, C. Issever, S. Istin, F. Ito, J. M. Iturbe Ponce, R. Iuppa, H. Iwasaki, J. M. Izen, V. Izzo, S. Jabbar, P. Jackson, V. Jain, K. B. Jakobi, K. Jakobs, S. Jakobsen, T. Jakoubek, D. O. Jamin, D. K. Jana, R. Jansky, J. Janssen, M. Janus, P. A. Janus, G. Jarlskog, N. Javadov, T. Javůrek, M. Javurkova, F. Jeanneau, L. Jeanty, J. Jejelava, A. Jelinskas, P. Jenni, C. Jeske, S. Jézéquel, H. Ji, J. Jia, H. Jiang, Y. Jiang, Z. Jiang, S. Jiggins, J. Jimenez Pena, S. Jin, A. Jinaru, O. Jinnouchi, H. Jivan, P. Johansson, K. A. Johns, C. A. Johnson, W. J. Johnson, K. Jon-And, R. W. L. Jones, S. Jones, T. J. Jones, J. Jongmanns, P. M. Jorge, J. Jovicevic, X. Ju, A. Juste Rozas, M. K. Köhler, A. Kaczmarska, M. Kado, H. Kagan, M. Kagan, S. J. Kahn, T. Kaji, E. Kajomovitz, C. W. Kalderon, A. Kaluza, S. Kama, A. Kamenshchikov, N. Kanaya, S. Kaneti, L. Kanjir, V. A. Kantserov, J. Kanzaki, B. Kaplan, L. S. Kaplan, D. Kar, K. Karakostas, N. Karastathis, M. J. Kareem, E. Karentzos, S. N. Karpov, Z. M. Karpova, K. Karthik, V. Kartvelishvili, A. N. Karyukhin, K. Kasahara, L. Kashif, R. D. Kass, A. Kastanas, Y. Kataoka, C. Kato, A. Katre, J. Katzy, K. Kawade, K. Kawagoe, T. Kawamoto, G. Kawamura, E. F. Kay, V. F. Kazanin, R. Keeler, R. Kehoe, J. S. Keller, J. J. Kempster, H. Keoshkerian, O. Kepka, B. P. Kerševan, S. Kersten, R. A. Keyes, M. Khader, F. Khalil-zada, A. Khanov, A. G. Kharlamov, T. Kharlamova, A. Khodinov, T. J. Khoo, V. Khovanskiy, E. Khramov, J. Khubua, S. Kido, C. R. Kilby, H. Y. Kim, S. H. Kim, Y. K. Kim, N. Kimura, O. M. Kind, B. T. King, D. Kirchmeier, J. Kirk, A. E. Kiryunin, T. Kishimoto, D. Kisielewska, K. Kiuchi, O. Kivernyk, E. Kladiva, T. Klapdor-Kleingrothaus, M. H. Klein, M. Klein, U. Klein, K. Kleinknecht, P. Klimek, A. Klimentov, R. Klingenberg, T. Klioutchnikova, E.-E. Kluge, P. Kluit, S. Kluth, J. Knapik, E. Kneringer, E. B. F. G. Knoops, A. Knue, A. Kobayashi, D. Kobayashi, T. Kobayashi, M. Kobel, M. Kocian, P. Kodys, T. Koffas, E. Koffeman, N. M. Köhler, T. Koi, M. Kolb, I. Koletsou, A. A. Komar, Y. Komori, T. Kondo, N. Kondrashova, K. Köneke, A. C. König, T. Kono, R. Konoplich, N. Konstantinidis, R. Kopeliansky, S. Koperny, A. K. Kopp, K. Korcyl, K. Kordas, A. Korn, A. A. Korol, I. Korolkov, E. V. Korolkova, O. Kortner, S. Kortner, T. Kosek, V. V. Kostyukhin, A. Kotwal, A. Koulouris, A. Kourkoumeli-Charalampidi, C. Kourkoumelis, V. Kouskoura, A. B. Kowalewska, R. Kowalewski, T. Z. Kowalski, C. Kozakai, W. Kozanecki, A. S. Kozhin, V. A. Kramarenko, G. Kramberger, D. Krasnopevtsev, M. W. Krasny, A. Krasznahorkay, D. Krauss, A. Kravchenko, J. A. Kremer, M. Kretz, J. Kretzschmar, K. Kreutzfeldt, P. Krieger, K. Krizka, K. Kroeninger, H. Kroha, J. Kroll, J. Kroseberg, J. Krstic, U. Kruchonak, H. Krüger, N. Krumnack, M. C. Kruse, M. Kruskal, T. Kubota, H. Kucuk, S. Kuday, J. T. Kuechler, S. Kuehn, A. Kugel, F. Kuger, T. Kuhl, V. Kukhtin, R. Kukla, Y. Kulchitsky, S. Kuleshov, Y. P. Kulinich, M. Kuna, T. Kunigo, A. Kupco, O. Kuprash, H. Kurashige, L. L. Kurchaninov, Y. A. Kurochkin, M. G. Kurth, V. Kus, E. S. Kuwertz, M. Kuze, J. Kvita, T. Kwan, D. Kyriazopoulos, A. La Rosa, J. L. La Rosa Navarro, L. La Rotonda, C. Lacasta, F. Lacava, J. Lacey, H. Lacker, D. Lacour, E. Ladygin, R. Lafaye, B. Laforge, T. Lagouri, S. Lai, S. Lammers, W. Lampl, E. Lançon, U. Landgraf, M. P. J. Landon, M. C. Lanfermann, V. S. Lang, J. C. Lange, A. J. Lankford, F. Lanni, K. Lantzsch, A. Lanza, A. Lapertosa, S. Laplace, J. F. Laporte, T. Lari, F. Lasagni Manghi, M. Lassnig, P. Laurelli, W. Lavrijsen, A. T. Law, P. Laycock, T. Lazovich, M. Lazzaroni, B. Le, O. Le Dortz, E. Le Guirriec, E. P. Le Quilleuc, M. LeBlanc, T. LeCompte, F. Ledroit-Guillon, C. A. Lee, S. C. Lee, L. Lee, B. Lefebvre, G. Lefebvre, M. Lefebvre, F. Legger, C. Leggett, A. Lehan, G. Lehmann Miotto, X. Lei, W. A. Leight, A. G. Leister, M. A. L. Leite, R. Leitner, D. Lellouch, B. Lemmer, K. J. C. Leney, T. Lenz, B. Lenzi, R. Leone, S. Leone, C. Leonidopoulos, G. Lerner, C. Leroy, A. A. J. Lesage, C. G. Lester, M. Levchenko, J. Levêque, D. Levin, L. J. Levinson, M. Levy, D. Lewis, M. Leyton, B. Li, C. Li, H. Li, L. Li, L. Li, Q. Li, S. Li, X. Li, Y. Li, Z. Liang, B. Liberti, A. Liblong, K. Lie, J. Liebal, W. Liebig, A. Limosani, S. C. Lin, T. H. Lin, B. E. Lindquist, A. E. Lionti, E. Lipeles, A. Lipniacka, M. Lisovyi, T. M. Liss, A. Lister, A. M. Litke, B. Liu, H. Liu, H. Liu, J. Liu, J. B. Liu, K. Liu, L. Liu, M. Liu, Y. L. Liu, Y. Liu, M. Livan, A. Lleres, J. Llorente Merino, S. L. Lloyd, C. Y. Lo, F. Lo Sterzo, E. M. Lobodzinska, P. Loch, F. K. Loebinger, K. M. Loew, A. Loginov, T. Lohse, K. Lohwasser, M. Lokajicek, B. A. Long, J. D. Long, R. E. Long, L. Longo, K. A. Looper, J. A. Lopez, D. Lopez Mateos, I. Lopez Paz, A. Lopez Solis, J. Lorenz, N. Lorenzo Martinez, M. Losada, P. J. Lösel, X. Lou, A. Lounis, J. Love, P. A. Love, H. Lu, N. Lu, Y. J. Lu, H. J. Lubatti, C. Luci, A. Lucotte, C. Luedtke, F. Luehring, W. Lukas, L. Luminari, O. Lundberg, B. Lund-Jensen, P. M. Luzi, D. Lynn, R. Lysak, E. Lytken, V. Lyubushkin, H. Ma, L. L. Ma, Y. Ma, G. Maccarrone, A. Macchiolo, C. M. Macdonald, B. Maček, J. Machado Miguens, D. Madaffari, R. Madar, H. J. Maddocks, W. F. Mader, A. Madsen, J. Maeda, S. Maeland, T. Maeno, A. Maevskiy, E. Magradze, J. Mahlstedt, C. Maiani, C. Maidantchik, A. A. Maier, T. Maier, A. Maio, S. Majewski, Y. Makida, N. Makovec, B. Malaescu, Pa. Malecki, V. P. Maleev, F. Malek, U. Mallik, D. Malon, C. Malone, S. Maltezos, S. Malyukov, J. Mamuzic, G. Mancini, L. Mandelli, I. Mandić, J. Maneira, L. Manhaes de Andrade Filho, J. Manjarres Ramos, A. Mann, A. Manousos, B. Mansoulie, J. D. Mansour, R. Mantifel, M. Mantoani, S. Manzoni, L. Mapelli, G. Marceca, L. March, G. Marchiori, M. Marcisovsky, M. Marjanovic, D. E. Marley, F. Marroquim, S. P. Marsden, Z. Marshall, M. U. F Martensson, S. Marti-Garcia, C. B. Martin, T. A. Martin, V. J. Martin, B. Martin dit Latour, M. Martinez, V. I. Martinez Outschoorn, S. Martin-Haugh, V. S. Martoiu, A. C. Martyniuk, A. Marzin, L. Masetti, T. Mashimo, R. Mashinistov, J. Masik, A. L. Maslennikov, L. Massa, P. Mastrandrea, A. Mastroberardino, T. Masubuchi, P. Mättig, J. Maurer, S. J. Maxfield, D. A. Maximov, R. Mazini, I. Maznas, S. M. Mazza, N. C. Mc Fadden, G. Mc Goldrick, S. P. Mc Kee, A. McCarn, R. L. McCarthy, T. G. McCarthy, L. I. McClymont, E. F. McDonald, J. A. Mcfayden, G. Mchedlidze, S. J. McMahon, P. C. McNamara, R. A. McPherson, S. Meehan, T. J. Megy, S. Mehlhase, A. Mehta, T. Meideck, K. Meier, C. Meineck, B. Meirose, D. Melini, B. R. Mellado Garcia, M. Melo, F. Meloni, S. B. Menary, L. Meng, X. T. Meng, A. Mengarelli, S. Menke, E. Meoni, S. Mergelmeyer, P. Mermod, L. Merola, C. Meroni, F. S. Merritt, A. Messina, J. Metcalfe, A. S. Mete, C. Meyer, J.-P. Meyer, J. Meyer, H. Meyer Zu Theenhausen, F. Miano, R. P. Middleton, S. Miglioranzi, L. Mijović, G. Mikenberg, M. Mikestikova, M. Mikuž, M. Milesi, A. Milic, D. W. Miller, C. Mills, A. Milov, D. A. Milstead, A. A. Minaenko, Y. Minami, I. A. Minashvili, A. I. Mincer, B. Mindur, M. Mineev, Y. Minegishi, Y. Ming, L. M. Mir, K. P. Mistry, T. Mitani, J. Mitrevski, V. A. Mitsou, A. Miucci, P. S. Miyagawa, A. Mizukami, J. U. Mjörnmark, M. Mlynarikova, T. Moa, K. Mochizuki, P. Mogg, S. Mohapatra, S. Molander, R. Moles-Valls, R. Monden, M. C. Mondragon, K. Mönig, J. Monk, E. Monnier, A. Montalbano, J. Montejo Berlingen, F. Monticelli, S. Monzani, R. W. Moore, N. Morange, D. Moreno, M. Moreno Llácer, P. Morettini, S. Morgenstern, D. Mori, T. Mori, M. Morii, M. Morinaga, V. Morisbak, A. K. Morley, G. Mornacchi, J. D. Morris, L. Morvaj, P. Moschovakos, M. Mosidze, H. J. Moss, J. Moss, K. Motohashi, R. Mount, E. Mountricha, E. J. W. Moyse, S. Muanza, R. D. Mudd, F. Mueller, J. Mueller, R. S. P. Mueller, D. Muenstermann, P. Mullen, G. A. Mullier, F. J. Munoz Sanchez, W. J. Murray, H. Musheghyan, M. Muškinja, A. G. Myagkov, M. Myska, B. P. Nachman, O. Nackenhorst, K. Nagai, R. Nagai, K. Nagano, Y. Nagasaka, K. Nagata, M. Nagel, E. Nagy, A. M. Nairz, Y. Nakahama, K. Nakamura, T. Nakamura, I. Nakano, R. F. Naranjo Garcia, R. Narayan, D. I. Narrias Villar, I. Naryshkin, T. Naumann, G. Navarro, R. Nayyar, H. A. Neal, P. Yu. Nechaeva, T. J. Neep, A. Negri, M. Negrini, S. Nektarijevic, C. Nellist, A. Nelson, S. Nemecek, P. Nemethy, A. A. Nepomuceno, M. Nessi, M. S. Neubauer, M. Neumann, R. M. Neves, P. Nevski, P. R. Newman, T. Y. Ng, T. Nguyen Manh, R. B. Nickerson, R. Nicolaidou, J. Nielsen, V. Nikolaenko, I. Nikolic-Audit, K. Nikolopoulos, J. K. Nilsen, P. Nilsson, Y. Ninomiya, A. Nisati, N. Nishu, R. Nisius, T. Nobe, Y. Noguchi, M. Nomachi, I. Nomidis, M. A. Nomura, T. Nooney, M. Nordberg, N. Norjoharuddeen, O. Novgorodova, S. Nowak, M. Nozaki, L. Nozka, K. Ntekas, E. Nurse, F. Nuti, D. C. O’Neil, A. A. O’Rourke, V. O’Shea, F. G. Oakham, H. Oberlack, T. Obermann, J. Ocariz, A. Ochi, I. Ochoa, J. P. Ochoa-Ricoux, S. Oda, S. Odaka, H. Ogren, A. Oh, S. H. Oh, C. C. Ohm, H. Ohman, H. Oide, H. Okawa, Y. Okumura, T. Okuyama, A. Olariu, L. F. Oleiro Seabra, S. A. Olivares Pino, D. Oliveira Damazio, A. Olszewski, J. Olszowska, A. Onofre, K. Onogi, P. U. E. Onyisi, M. J. Oreglia, Y. Oren, D. Orestano, N. Orlando, R. S. Orr, B. Osculati, R. Ospanov, G. Otero y Garzon, H. Otono, M. Ouchrif, F. Ould-Saada, A. Ouraou, K. P. Oussoren, Q. Ouyang, M. Owen, R. E. Owen, V. E. Ozcan, N. Ozturk, K. Pachal, A. Pacheco Pages, L. Pacheco Rodriguez, C. Padilla Aranda, S. Pagan Griso, M. Paganini, F. Paige, P. Pais, G. Palacino, S. Palazzo, S. Palestini, M. Palka, D. Pallin, E. St. Panagiotopoulou, I. Panagoulias, C. E. Pandini, J. G. Panduro Vazquez, P. Pani, S. Panitkin, D. Pantea, L. Paolozzi, Th. D. Papadopoulou, K. Papageorgiou, A. Paramonov, D. Paredes Hernandez, A. J. Parker, M. A. Parker, K. A. Parker, F. Parodi, J. A. Parsons, U. Parzefall, V. R. Pascuzzi, J. M. Pasner, E. Pasqualucci, S. Passaggio, Fr. Pastore, S. Pataraia, J. R. Pater, T. Pauly, J. Pearce, B. Pearson, L. E. Pedersen, S. Pedraza Lopez, R. Pedro, S. V. Peleganchuk, O. Penc, C. Peng, H. Peng, J. Penwell, B. S. Peralva, M. M. Perego, D. V. Perepelitsa, L. Perini, H. Pernegger, S. Perrella, R. Peschke, V. D. Peshekhonov, K. Peters, R. F. Y. Peters, B. A. Petersen, T. C. Petersen, E. Petit, A. Petridis, C. Petridou, P. Petroff, E. Petrolo, M. Petrov, F. Petrucci, N. E. Pettersson, A. Peyaud, R. Pezoa, P. W. Phillips, G. Piacquadio, E. Pianori, A. Picazio, E. Piccaro, M. A. Pickering, R. Piegaia, J. E. Pilcher, A. D. Pilkington, A. W. J. Pin, M. Pinamonti, J. L. Pinfold, H. Pirumov, M. Pitt, L. Plazak, M.-A. Pleier, V. Pleskot, E. Plotnikova, D. Pluth, P. Podberezko, R. Poettgen, L. Poggioli, D. Pohl, G. Polesello, A. Poley, A. Policicchio, R. Polifka, A. Polini, C. S. Pollard, V. Polychronakos, K. Pommès, L. Pontecorvo, B. G. Pope, G. A. Popeneciu, A. Poppleton, S. Pospisil, K. Potamianos, I. N. Potrap, C. J. Potter, C. T. Potter, G. Poulard, J. Poveda, M. E. Pozo Astigarraga, P. Pralavorio, A. Pranko, S. Prell, D. Price, L. E. Price, M. Primavera, S. Prince, K. Prokofiev, F. Prokoshin, S. Protopopescu, J. Proudfoot, M. Przybycien, D. Puddu, A. Puri, P. Puzo, J. Qian, G. Qin, Y. Qin, A. Quadt, W. B. Quayle, M. Queitsch-Maitland, D. Quilty, S. Raddum, V. Radeka, V. Radescu, S. K. Radhakrishnan, P. Radloff, P. Rados, F. Ragusa, G. Rahal, J. A. Raine, S. Rajagopalan, C. Rangel-Smith, M. G. Ratti, D. M. Rauch, F. Rauscher, S. Rave, T. Ravenscroft, I. Ravinovich, M. Raymond, A. L. Read, N. P. Readioff, M. Reale, D. M. Rebuzzi, A. Redelbach, G. Redlinger, R. Reece, R. G. Reed, K. Reeves, L. Rehnisch, J. Reichert, A. Reiss, C. Rembser, H. Ren, M. Rescigno, S. Resconi, E. D. Resseguie, S. Rettie, E. Reynolds, O. L. Rezanova, P. Reznicek, R. Rezvani, R. Richter, S. Richter, E. Richter-Was, O. Ricken, M. Ridel, P. Rieck, C. J. Riegel, J. Rieger, O. Rifki, M. Rijssenbeek, A. Rimoldi, M. Rimoldi, L. Rinaldi, B. Ristić, E. Ritsch, I. Riu, F. Rizatdinova, E. Rizvi, C. Rizzi, R. T. Roberts, S. H. Robertson, A. Robichaud-Veronneau, D. Robinson, J. E. M. Robinson, A. Robson, C. Roda, Y. Rodina, A. Rodriguez Perez, D. Rodriguez Rodriguez, S. Roe, C. S. Rogan, O. Røhne, J. Roloff, A. Romaniouk, M. Romano, S. M. Romano Saez, E. Romero Adam, N. Rompotis, M. Ronzani, L. Roos, S. Rosati, K. Rosbach, P. Rose, N.-A. Rosien, V. Rossetti, E. Rossi, L. P. Rossi, J. H. N. Rosten, R. Rosten, M. Rotaru, I. Roth, J. Rothberg, D. Rousseau, A. Rozanov, Y. Rozen, X. Ruan, F. Rubbo, F. Rühr, A. Ruiz-Martinez, Z. Rurikova, N. A. Rusakovich, A. Ruschke, H. L. Russell, J. P. Rutherfoord, N. Ruthmann, Y. F. Ryabov, M. Rybar, G. Rybkin, S. Ryu, A. Ryzhov, G. F. Rzehorz, A. F. Saavedra, G. Sabato, S. Sacerdoti, H. F.-W. Sadrozinski, R. Sadykov, F. Safai Tehrani, P. Saha, M. Sahinsoy, M. Saimpert, T. Saito, H. Sakamoto, Y. Sakurai, G. Salamanna, J. E. Salazar Loyola, D. Salek, P. H. Sales De Bruin, D. Salihagic, A. Salnikov, J. Salt, D. Salvatore, F. Salvatore, A. Salvucci, A. Salzburger, D. Sammel, D. Sampsonidis, J. Sánchez, V. Sanchez Martinez, A. Sanchez Pineda, H. Sandaker, R. L. Sandbach, C. O. Sander, M. Sandhoff, C. Sandoval, D. P. C. Sankey, M. Sannino, A. Sansoni, C. Santoni, R. Santonico, H. Santos, I. Santoyo Castillo, K. Sapp, A. Sapronov, J. G. Saraiva, B. Sarrazin, O. Sasaki, K. Sato, E. Sauvan, G. Savage, P. Savard, N. Savic, C. Sawyer, L. Sawyer, J. Saxon, C. Sbarra, A. Sbrizzi, T. Scanlon, D. A. Scannicchio, M. Scarcella, V. Scarfone, J. Schaarschmidt, P. Schacht, B. M. Schachtner, D. Schaefer, L. Schaefer, R. Schaefer, J. Schaeffer, S. Schaepe, S. Schaetzel, U. Schäfer, A. C. Schaffer, D. Schaile, R. D. Schamberger, V. Scharf, V. A. Schegelsky, D. Scheirich, M. Schernau, C. Schiavi, S. Schier, C. Schillo, M. Schioppa, S. Schlenker, K. R. Schmidt-Sommerfeld, K. Schmieden, C. Schmitt, S. Schmitt, S. Schmitz, B. Schneider, U. Schnoor, L. Schoeffel, A. Schoening, B. D. Schoenrock, E. Schopf, M. Schott, J. F. P. Schouwenberg, J. Schovancova, S. Schramm, N. Schuh, A. Schulte, M. J. Schultens, H.-C. Schultz-Coulon, H. Schulz, M. Schumacher, B. A. Schumm, Ph. Schune, A. Schwartzman, T. A. Schwarz, H. Schweiger, Ph. Schwemling, R. Schwienhorst, J. Schwindling, T. Schwindt, G. Sciolla, F. Scuri, F. Scutti, J. Searcy, P. Seema, S. C. Seidel, A. Seiden, J. M. Seixas, G. Sekhniaidze, K. Sekhon, S. J. Sekula, N. Semprini-Cesari, C. Serfon, L. Serin, L. Serkin, M. Sessa, R. Seuster, H. Severini, T. Sfiligoj, F. Sforza, A. Sfyrla, E. Shabalina, N. W. Shaikh, L. Y. Shan, R. Shang, J. T. Shank, M. Shapiro, P. B. Shatalov, K. Shaw, S. M. Shaw, A. Shcherbakova, C. Y. Shehu, Y. Shen, P. Sherwood, L. Shi, S. Shimizu, C. O. Shimmin, M. Shimojima, S. Shirabe, M. Shiyakova, J. Shlomi, A. Shmeleva, D. Shoaleh Saadi, M. J. Shochet, S. Shojaii, D. R. Shope, S. Shrestha, E. Shulga, M. A. Shupe, P. Sicho, A. M. Sickles, P. E. Sidebo, E. Sideras Haddad, O. Sidiropoulou, D. Sidorov, A. Sidoti, F. Siegert, Dj. Sijacki, J. Silva, S. B. Silverstein, V. Simak, Lj. Simic, S. Simion, E. Simioni, B. Simmons, M. Simon, P. Sinervo, N. B. Sinev, M. Sioli, G. Siragusa, I. Siral, S. Yu. Sivoklokov, J. Sjölin, M. B. Skinner, P. Skubic, M. Slater, T. Slavicek, M. Slawinska, K. Sliwa, R. Slovak, V. Smakhtin, B. H. Smart, L. Smestad, J. Smiesko, S. Yu. Smirnov, Y. Smirnov, L. N. Smirnova, O. Smirnova, J. W. Smith, M. N. K. Smith, R. W. Smith, M. Smizanska, K. Smolek, A. A. Snesarev, I. M. Snyder, S. Snyder, R. Sobie, F. Socher, A. Soffer, D. A. Soh, G. Sokhrannyi, C. A. Solans Sanchez, M. Solar, E. Yu. Soldatov, U. Soldevila, A. A. Solodkov, A. Soloshenko, O. V. Solovyanov, V. Solovyev, P. Sommer, H. Son, H. Y. Song, A. Sopczak, V. Sorin, D. Sosa, C. L. Sotiropoulou, R. Soualah, A. M. Soukharev, D. South, B. C. Sowden, S. Spagnolo, M. Spalla, M. Spangenberg, F. Spanò, D. Sperlich, F. Spettel, T. M. Spieker, R. Spighi, G. Spigo, L. A. Spiller, M. Spousta, R. D. St. Denis, A. Stabile, R. Stamen, S. Stamm, E. Stanecka, R. W. Stanek, C. Stanescu, M. M. Stanitzki, S. Stapnes, E. A. Starchenko, G. H. Stark, J. Stark, S. H Stark, P. Staroba, P. Starovoitov, S. Stärz, R. Staszewski, P. Steinberg, B. Stelzer, H. J. Stelzer, O. Stelzer-Chilton, H. Stenzel, G. A. Stewart, J. A. Stillings, M. C. Stockton, M. Stoebe, G. Stoicea, P. Stolte, S. Stonjek, A. R. Stradling, A. Straessner, M. E. Stramaglia, J. Strandberg, S. Strandberg, A. Strandlie, M. Strauss, P. Strizenec, R. Ströhmer, D. M. Strom, R. Stroynowski, A. Strubig, S. A. Stucci, B. Stugu, N. A. Styles, D. Su, J. Su, S. Suchek, Y. Sugaya, M. Suk, V. V. Sulin, S. Sultansoy, T. Sumida, S. Sun, X. Sun, K. Suruliz, C. J. E. Suster, M. R. Sutton, S. Suzuki, M. Svatos, M. Swiatlowski, S. P. Swift, I. Sykora, T. Sykora, D. Ta, K. Tackmann, J. Taenzer, A. Taffard, R. Tafirout, N. Taiblum, H. Takai, R. Takashima, T. Takeshita, Y. Takubo, M. Talby, A. A. Talyshev, J. Tanaka, M. Tanaka, R. Tanaka, S. Tanaka, R. Tanioka, B. B. Tannenwald, S. Tapia Araya, S. Tapprogge, S. Tarem, G. F. Tartarelli, P. Tas, M. Tasevsky, T. Tashiro, E. Tassi, A. Tavares Delgado, Y. Tayalati, A. C. Taylor, G. N. Taylor, P. T. E. Taylor, W. Taylor, P. Teixeira-Dias, D. Temple, H. Ten Kate, P. K. Teng, J. J. Teoh, F. Tepel, S. Terada, K. Terashi, J. Terron, S. Terzo, M. Testa, R. J. Teuscher, T. Theveneaux-Pelzer, J. P. Thomas, J. Thomas-Wilsker, P. D. Thompson, A. S. Thompson, L. A. Thomsen, E. Thomson, M. J. Tibbetts, R. E. Ticse Torres, V. O. Tikhomirov, Yu. A. Tikhonov, S. Timoshenko, P. Tipton, S. Tisserant, K. Todome, S. Todorova-Nova, J. Tojo, S. Tokár, K. Tokushuku, E. Tolley, L. Tomlinson, M. Tomoto, L. Tompkins, K. Toms, B. Tong, P. Tornambe, E. Torrence, H. Torres, E. Torró Pastor, J. Toth, F. Touchard, D. R. Tovey, C. J. Treado, T. Trefzger, A. Tricoli, I. M. Trigger, S. Trincaz-Duvoid, M. F. Tripiana, W. Trischuk, B. Trocmé, A. Trofymov, C. Troncon, M. Trottier-McDonald, M. Trovatelli, L. Truong, M. Trzebinski, A. Trzupek, K. W. Tsang, J. C.-L. Tseng, P. V. Tsiareshka, G. Tsipolitis, N. Tsirintanis, S. Tsiskaridze, V. Tsiskaridze, E. G. Tskhadadze, K. M. Tsui, I. I. Tsukerman, V. Tsulaia, S. Tsuno, D. Tsybychev, Y. Tu, A. Tudorache, V. Tudorache, T. T. Tulbure, A. N. Tuna, S. A. Tupputi, S. Turchikhin, D. Turgeman, I. Turk Cakir, R. Turra, P. M. Tuts, G. Ucchielli, I. Ueda, M. Ughetto, F. Ukegawa, G. Unal, A. Undrus, G. Unel, F. C. Ungaro, Y. Unno, C. Unverdorben, J. Urban, P. Urquijo, P. Urrejola, G. Usai, J. Usui, L. Vacavant, V. Vacek, B. Vachon, C. Valderanis, E. Valdes Santurio, N. Valencic, S. Valentinetti, A. Valero, L. Valéry, S. Valkar, A. Vallier, J. A. Valls Ferrer, W. Van Den Wollenberg, H. van der Graaf, N. van Eldik, P. van Gemmeren, J. Van Nieuwkoop, I. van Vulpen, M. C. van Woerden, M. Vanadia, W. Vandelli, R. Vanguri, A. Vaniachine, P. Vankov, G. Vardanyan, R. Vari, E. W. Varnes, C. Varni, T. Varol, D. Varouchas, A. Vartapetian, K. E. Varvell, J. G. Vasquez, G. A. Vasquez, F. Vazeille, T. Vazquez Schroeder, J. Veatch, V. Veeraraghavan, L. M. Veloce, F. Veloso, S. Veneziano, A. Ventura, M. Venturi, N. Venturi, A. Venturini, V. Vercesi, M. Verducci, W. Verkerke, J. C. Vermeulen, M. C. Vetterli, N. Viaux Maira, O. Viazlo, I. Vichou, T. Vickey, O. E. Vickey Boeriu, G. H. A. Viehhauser, S. Viel, L. Vigani, M. Villa, M. Villaplana Perez, E. Vilucchi, M. G. Vincter, V. B. Vinogradov, A. Vishwakarma, C. Vittori, I. Vivarelli, S. Vlachos, M. Vlasak, M. Vogel, P. Vokac, G. Volpi, M. Volpi, H. von der Schmitt, E. von Toerne, V. Vorobel, K. Vorobev, M. Vos, R. Voss, J. H. Vossebeld, N. Vranjes, M. Vranjes Milosavljevic, V. Vrba, M. Vreeswijk, R. Vuillermet, I. Vukotic, P. Wagner, W. Wagner, H. Wahlberg, S. Wahrmund, J. Wakabayashi, J. Walder, R. Walker, W. Walkowiak, V. Wallangen, C. Wang, C. Wang, F. Wang, H. Wang, H. Wang, J. Wang, J. Wang, Q. Wang, R. Wang, S. M. Wang, T. Wang, W. Wang, W. Wang, C. Wanotayaroj, A. Warburton, C. P. Ward, D. R. Wardrope, A. Washbrook, P. M. Watkins, A. T. Watson, M. F. Watson, G. Watts, S. Watts, B. M. Waugh, A. F. Webb, S. Webb, M. S. Weber, S. W. Weber, S. A. Weber, J. S. Webster, A. R. Weidberg, B. Weinert, J. Weingarten, C. Weiser, H. Weits, P. S. Wells, T. Wenaus, T. Wengler, S. Wenig, N. Wermes, M. D. Werner, P. Werner, M. Wessels, K. Whalen, N. L. Whallon, A. M. Wharton, A. White, M. J. White, R. White, D. Whiteson, F. J. Wickens, W. Wiedenmann, M. Wielers, C. Wiglesworth, L. A. M. Wiik-Fuchs, A. Wildauer, F. Wilk, H. G. Wilkens, H. H. Williams, S. Williams, C. Willis, S. Willocq, J. A. Wilson, I. Wingerter-Seez, F. Winklmeier, O. J. Winston, B. T. Winter, M. Wittgen, M. Wobisch, T. M. H. Wolf, R. Wolff, M. W. Wolter, H. Wolters, S. D. Worm, B. K. Wosiek, J. Wotschack, M. J. Woudstra, K. W. Wozniak, M. Wu, S. L. Wu, X. Wu, Y. Wu, T. R. Wyatt, B. M. Wynne, S. Xella, Z. Xi, L. Xia, D. Xu, L. Xu, B. Yabsley, S. Yacoob, D. Yamaguchi, Y. Yamaguchi, A. Yamamoto, S. Yamamoto, T. Yamanaka, K. Yamauchi, Y. Yamazaki, Z. Yan, H. Yang, H. Yang, Y. Yang, Z. Yang, W.-M. Yao, Y. C. Yap, Y. Yasu, E. Yatsenko, K. H. Yau Wong, J. Ye, S. Ye, I. Yeletskikh, E. Yildirim, K. Yorita, K. Yoshihara, C. Young, C. J. S. Young, S. Youssef, D. R. Yu, J. Yu, J. Yu, L. Yuan, S. P. Y. Yuen, I. Yusuff, B. Zabinski, G. Zacharis, R. Zaidan, A. M. Zaitsev, N. Zakharchuk, J. Zalieckas, A. Zaman, S. Zambito, D. Zanzi, C. Zeitnitz, M. Zeman, A. Zemla, J. C. Zeng, Q. Zeng, O. Zenin, T. Ženiš, D. Zerwas, D. Zhang, F. Zhang, G. Zhang, H. Zhang, J. Zhang, L. Zhang, L. Zhang, M. Zhang, R. Zhang, R. Zhang, X. Zhang, Y. Zhang, Z. Zhang, X. Zhao, Y. Zhao, Z. Zhao, A. Zhemchugov, J. Zhong, B. Zhou, C. Zhou, L. Zhou, M. Zhou, M. Zhou, N. Zhou, C. G. Zhu, H. Zhu, J. Zhu, Y. Zhu, X. Zhuang, K. Zhukov, A. Zibell, D. Zieminska, N. I. Zimine, C. Zimmermann, S. Zimmermann, Z. Zinonos, M. Zinser, M. Ziolkowski, L. Živković, G. Zobernig, A. Zoccoli, R. Zou, M. zur Nedden, L. Zwalinski, ATLAS Collaboration

**Affiliations:** 1 Department of Physics, University of Adelaide, Adelaide, Australia; 2 Physics Department, SUNY Albany, Albany, NY USA; 3 Department of Physics, University of Alberta, Edmonton, AB Canada; 4 Department of Physics, Ankara University, Ankara, Turkey; 5 Istanbul Aydin University, Istanbul, Turkey; 6 Division of Physics, TOBB University of Economics and Technology, Ankara, Turkey; 7 LAPP, CNRS/IN2P3 and Université Savoie Mont Blanc, Annecy-le-Vieux, France; 8 High Energy Physics Division, Argonne National Laboratory, Argonne, IL USA; 9 Department of Physics, University of Arizona, Tucson, AZ USA; 10 Department of Physics, The University of Texas at Arlington, Arlington, TX USA; 11 Physics Department, National and Kapodistrian University of Athens, Athens, Greece; 12 Physics Department, National Technical University of Athens, Zografou, Greece; 13 Department of Physics, The University of Texas at Austin, Austin, TX USA; 14 Institute of Physics, Azerbaijan Academy of Sciences, Baku, Azerbaijan; 15 Institut de Física d’Altes Energies (IFAE), The Barcelona Institute of Science and Technology, Barcelona, Spain; 16 Institute of Physics, University of Belgrade, Belgrade, Serbia; 17 Department for Physics and Technology, University of Bergen, Bergen, Norway; 18 Physics Division, Lawrence Berkeley National Laboratory and University of California, Berkeley, CA USA; 19 Department of Physics, Humboldt University, Berlin, Germany; 20 Albert Einstein Center for Fundamental Physics and Laboratory for High Energy Physics, University of Bern, Bern, Switzerland; 21 School of Physics and Astronomy, University of Birmingham, Birmingham, UK; 22 Department of Physics, Bogazici University, Istanbul, Turkey; 23 Department of Physics Engineering, Gaziantep University, Gaziantep, Turkey; 24 Faculty of Engineering and Natural Sciences, Istanbul Bilgi University, Istanbul, Turkey; 25 Faculty of Engineering and Natural Sciences, Bahcesehir University, Istanbul, Turkey; 26 Centro de Investigaciones, Universidad Antonio Narino, Bogota, Colombia; 27 INFN Sezione di Bologna, Bologna, Italy; 28 Dipartimento di Fisica e Astronomia, Università di Bologna, Bologna, Italy; 29 Physikalisches Institut, University of Bonn, Bonn, Germany; 30 Department of Physics, Boston University, Boston, MA USA; 31 Department of Physics, Brandeis University, Waltham, MA USA; 32 Universidade Federal do Rio De Janeiro COPPE/EE/IF, Rio de Janeiro, Brazil; 33 Electrical Circuits Department, Federal University of Juiz de Fora (UFJF), Juiz de Fora, Brazil; 34 Federal University of Sao Joao del Rei (UFSJ), Sao Joao del Rei, Brazil; 35 Instituto de Fisica, Universidade de Sao Paulo, Sao Paulo, Brazil; 36 Physics Department, Brookhaven National Laboratory, Upton, NY USA; 37 Transilvania University of Brasov, Brasov, Romania; 38 Horia Hulubei National Institute of Physics and Nuclear Engineering, Bucharest, Romania; 39 Department of Physics, Alexandru Ioan Cuza University of Iasi, Iasi, Romania; 40 Physics Department, National Institute for Research and Development of Isotopic and Molecular Technologies, Cluj Napoca, Romania; 41 University Politehnica Bucharest, Bucharest, Romania; 42 West University in Timisoara, Timisoara, Romania; 43 Departamento de Física, Universidad de Buenos Aires, Buenos Aires, Argentina; 44 Cavendish Laboratory, University of Cambridge, Cambridge, UK; 45 Department of Physics, Carleton University, Ottawa, ON Canada; 46 CERN, Geneva, Switzerland; 47 Enrico Fermi Institute, University of Chicago, Chicago, IL USA; 48 Departamento de Física, Pontificia Universidad Católica de Chile, Santiago, Chile; 49 Departamento de Física, Universidad Técnica Federico Santa María, Valparaiso, Chile; 50 Institute of High Energy Physics, Chinese Academy of Sciences, Beijing, China; 51 Department of Physics, Nanjing University, Nanjing, Jiangsu China; 52 Physics Department, Tsinghua University, Beijing, 100084 China; 53 Department of Modern Physics, University of Science and Technology of China, Hefei, Anhui China; 54 School of Physics, Shandong University, Jinan, Shandong China; 55 Department of Physics and Astronomy, Key Laboratory for Particle Physics, Astrophysics and Cosmology, Ministry of Education; Shanghai Key Laboratory for Particle Physics and Cosmology, Shanghai Jiao Tong University, Shanghai (also at PKU-CHEP), Shanghai, China; 56 Université Clermont Auvergne, CNRS/IN2P3, LPC, Clermont-Ferrand, France; 57 Nevis Laboratory, Columbia University, Irvington, NY USA; 58 Niels Bohr Institute, University of Copenhagen, Kobenhavn, Denmark; 59 INFN Gruppo Collegato di Cosenza, Laboratori Nazionali di Frascati, Frascati, Italy; 60 Dipartimento di Fisica, Università della Calabria, Rende, Italy; 61 Faculty of Physics and Applied Computer Science, AGH University of Science and Technology, Krakow, Poland; 62 Marian Smoluchowski Institute of Physics, Jagiellonian University, Krakow, Poland; 63 Institute of Nuclear Physics, Polish Academy of Sciences, Krakow, Poland; 64 Physics Department, Southern Methodist University, Dallas, TX USA; 65 Physics Department, University of Texas at Dallas, Richardson, TX USA; 66 DESY, Hamburg and Zeuthen, Germany; 67 Lehrstuhl für Experimentelle Physik IV, Technische Universität Dortmund, Dortmund, Germany; 68 Institut für Kern- und Teilchenphysik, Technische Universität Dresden, Dresden, Germany; 69 Department of Physics, Duke University, Durham, NC USA; 70 SUPA-School of Physics and Astronomy, University of Edinburgh, Edinburgh, UK; 71 INFN Laboratori Nazionali di Frascati, Frascati, Italy; 72 Fakultät für Mathematik und Physik, Albert-Ludwigs-Universität, Freiburg, Germany; 73 Departement de Physique Nucleaire et Corpusculaire, Université de Genève, Genova, Switzerland; 74 INFN Sezione di Genova, Genova, Italy; 75 Dipartimento di Fisica, Università di Genova, Genova, Italy; 76 E. Andronikashvili Institute of Physics, Iv. Javakhishvili Tbilisi State University, Tbilisi, Georgia; 77 High Energy Physics Institute, Tbilisi State University, Tbilisi, Georgia; 78 II Physikalisches Institut, Justus-Liebig-Universität Giessen, Giessen, Germany; 79 SUPA-School of Physics and Astronomy, University of Glasgow, Glasgow, UK; 80 II Physikalisches Institut, Georg-August-Universität, Göttingen, Germany; 81 Laboratoire de Physique Subatomique et de Cosmologie, Université Grenoble-Alpes, CNRS/IN2P3, Grenoble, France; 82 Laboratory for Particle Physics and Cosmology, Harvard University, Cambridge, MA USA; 83 Kirchhoff-Institut für Physik, Ruprecht-Karls-Universität Heidelberg, Heidelberg, Germany; 84 Physikalisches Institut, Ruprecht-Karls-Universität Heidelberg, Heidelberg, Germany; 85 ZITI Institut für technische Informatik, Ruprecht-Karls-Universität Heidelberg, Mannheim, Germany; 86 Faculty of Applied Information Science, Hiroshima Institute of Technology, Hiroshima, Japan; 87 Department of Physics, The Chinese University of Hong Kong, Shatin, N.T. Hong Kong; 88 Department of Physics, The University of Hong Kong, Hong Kong, China; 89 Department of Physics and Institute for Advanced Study, The Hong Kong University of Science and Technology, Clear Water Bay, Kowloon, Hong Kong, China; 90 Department of Physics, National Tsing Hua University, Hsinchu, Taiwan; 91 Department of Physics, Indiana University, Bloomington, IN USA; 92 Institut für Astro- und Teilchenphysik, Leopold-Franzens-Universität, Innsbruck, Austria; 93 University of Iowa, Iowa City, IA USA; 94 Department of Physics and Astronomy, Iowa State University, Ames, IA USA; 95 Joint Institute for Nuclear Research, JINR Dubna, Dubna, Russia; 96 KEK, High Energy Accelerator Research Organization, Tsukuba, Japan; 97 Graduate School of Science, Kobe University, Kobe, Japan; 98 Faculty of Science, Kyoto University, Kyoto, Japan; 99 Kyoto University of Education, Kyoto, Japan; 100 Department of Physics, Kyushu University, Fukuoka, Japan; 101 Instituto de Física La Plata, Universidad Nacional de La Plata and CONICET, La Plata, Argentina; 102 Physics Department, Lancaster University, Lancaster, UK; 103 INFN Sezione di Lecce, Lecce, Italy; 104 Dipartimento di Matematica e Fisica, Università del Salento, Lecce, Italy; 105 Oliver Lodge Laboratory, University of Liverpool, Liverpool, UK; 106 Department of Experimental Particle Physics, Jožef Stefan Institute and Department of Physics, University of Ljubljana, Ljubljana, Slovenia; 107 School of Physics and Astronomy, Queen Mary University of London, London, UK; 108 Department of Physics, Royal Holloway University of London, Surrey, UK; 109 Department of Physics and Astronomy, University College London, London, UK; 110 Louisiana Tech University, Ruston, LA USA; 111 Laboratoire de Physique Nucléaire et de Hautes Energies, UPMC and Université Paris-Diderot and CNRS/IN2P3, Paris, France; 112 Fysiska institutionen, Lunds universitet, Lund, Sweden; 113 Departamento de Fisica Teorica C-15, Universidad Autonoma de Madrid, Madrid, Spain; 114 Institut für Physik, Universität Mainz, Mainz, Germany; 115 School of Physics and Astronomy, University of Manchester, Manchester, UK; 116 CPPM, Aix-Marseille Université and CNRS/IN2P3, Marseille, France; 117 Department of Physics, University of Massachusetts, Amherst, MA USA; 118 Department of Physics, McGill University, Montreal, QC Canada; 119 School of Physics, University of Melbourne, Victoria, Australia; 120 Department of Physics, The University of Michigan, Ann Arbor, MI USA; 121 Department of Physics and Astronomy, Michigan State University, East Lansing, MI USA; 122 INFN Sezione di Milano, Milano, Italy; 123 Dipartimento di Fisica, Università di Milano, Milano, Italy; 124 B.I. Stepanov Institute of Physics, National Academy of Sciences of Belarus, Minsk, Republic of Belarus; 125 Research Institute for Nuclear Problems of Byelorussian State University, Minsk, Republic of Belarus; 126 Group of Particle Physics, University of Montreal, Montreal, QC Canada; 127 P.N. Lebedev Physical Institute of the Russian Academy of Sciences, Moscow, Russia; 128 Institute for Theoretical and Experimental Physics (ITEP), Moscow, Russia; 129 National Research Nuclear University MEPhI, Moscow, Russia; 130 D.V. Skobeltsyn Institute of Nuclear Physics, M.V. Lomonosov Moscow State University, Moscow, Russia; 131 Fakultät für Physik, Ludwig-Maximilians-Universität München, München, Germany; 132 Max-Planck-Institut für Physik (Werner-Heisenberg-Institut), München, Germany; 133 Nagasaki Institute of Applied Science, Nagasaki, Japan; 134 Graduate School of Science and Kobayashi-Maskawa Institute, Nagoya University, Nagoya, Japan; 135 INFN Sezione di Napoli, Napoli, Italy; 136 Dipartimento di Fisica, Università di Napoli, Napoli, Italy; 137 Department of Physics and Astronomy, University of New Mexico, Albuquerque, NM USA; 138 Institute for Mathematics, Astrophysics and Particle Physics, Radboud University Nijmegen/Nikhef, Nijmegen, Netherlands; 139 Nikhef National Institute for Subatomic Physics and University of Amsterdam, Amsterdam, Netherlands; 140 Department of Physics, Northern Illinois University, DeKalb, IL USA; 141 Budker Institute of Nuclear Physics, SB RAS, Novosibirsk, Russia; 142 Department of Physics, New York University, New York, NY USA; 143 Ohio State University, Columbus, OH USA; 144 Faculty of Science, Okayama University, Okayama, Japan; 145 Homer L. Dodge Department of Physics and Astronomy, University of Oklahoma, Norman, OK USA; 146 Department of Physics, Oklahoma State University, Stillwater, OK USA; 147 Palacký University, RCPTM, Olomouc, Czech Republic; 148 Center for High Energy Physics, University of Oregon, Eugene, OR USA; 149 LAL, Univ. Paris-Sud, CNRS/IN2P3, Université Paris-Saclay, Orsay, France; 150 Graduate School of Science, Osaka University, Osaka, Japan; 151 Department of Physics, University of Oslo, Oslo, Norway; 152 Department of Physics, Oxford University, Oxford, UK; 153 INFN Sezione di Pavia, Pavia, Italy; 154 Dipartimento di Fisica, Università di Pavia, Pavia, Italy; 155 Department of Physics, University of Pennsylvania, Philadelphia, PA USA; 156 National Research Centre “Kurchatov Institute” B.P. Konstantinov Petersburg Nuclear Physics Institute, St. Petersburg, Russia; 157 INFN Sezione di Pisa, Pisa, Italy; 158 Dipartimento di Fisica E. Fermi, Università di Pisa, Pisa, Italy; 159 Department of Physics and Astronomy, University of Pittsburgh, Pittsburgh, PA USA; 160 Laboratório de Instrumentação e Física Experimental de Partículas-LIP, Lisboa, Portugal; 161 Faculdade de Ciências, Universidade de Lisboa, Lisboa, Portugal; 162 Department of Physics, University of Coimbra, Coimbra, Portugal; 163 Centro de Física Nuclear da Universidade de Lisboa, Lisboa, Portugal; 164 Departamento de Fisica, Universidade do Minho, Braga, Portugal; 165 Departamento de Fisica Teorica y del Cosmos and CAFPE, Universidad de Granada, Granada, Spain; 166 Dep Fisica and CEFITEC of Faculdade de Ciencias e Tecnologia, Universidade Nova de Lisboa, Caparica, Portugal; 167 Institute of Physics, Academy of Sciences of the Czech Republic, Praha, Czech Republic; 168 Czech Technical University in Prague, Praha, Czech Republic; 169 Faculty of Mathematics and Physics, Charles University, Prague, Czech Republic; 170 State Research Center Institute for High Energy Physics (Protvino), NRC KI, Protvino, Russia; 171 Particle Physics Department, Rutherford Appleton Laboratory, Didcot, UK; 172 INFN Sezione di Roma, Roma, Italy; 173 Dipartimento di Fisica, Sapienza Università di Roma, Roma, Italy; 174 INFN Sezione di Roma Tor Vergata, Roma, Italy; 175 Dipartimento di Fisica, Università di Roma Tor Vergata, Roma, Italy; 176 INFN Sezione di Roma Tre, Roma, Italy; 177 Dipartimento di Matematica e Fisica, Università Roma Tre, Roma, Italy; 178 Faculté des Sciences Ain Chock, Réseau Universitaire de Physique des Hautes Energies-Université Hassan II, Casablanca, Morocco; 179 Centre National de l’Energie des Sciences Techniques Nucleaires, Rabat, Morocco; 180 Faculté des Sciences Semlalia, Université Cadi Ayyad, LPHEA-Marrakech, Marrakech, Morocco; 181 Faculté des Sciences, Université Mohamed Premier and LPTPM, Oujda, Morocco; 182 Faculté des Sciences, Université Mohammed V, Rabat, Morocco; 183 DSM/IRFU (Institut de Recherches sur les Lois Fondamentales de l’Univers), CEA Saclay (Commissariat à l’Energie Atomique et aux Energies Alternatives), Gif-sur-Yvette, France; 184 Santa Cruz Institute for Particle Physics, University of California Santa Cruz, Santa Cruz, CA USA; 185 Department of Physics, University of Washington, Seattle, WA USA; 186 Department of Physics and Astronomy, University of Sheffield, Sheffield, UK; 187 Department of Physics, Shinshu University, Nagano, Japan; 188 Department Physik, Universität Siegen, Siegen, Germany; 189 Department of Physics, Simon Fraser University, Burnaby, BC Canada; 190 SLAC National Accelerator Laboratory, Stanford, CA USA; 191 Faculty of Mathematics, Physics and Informatics, Comenius University, Bratislava, Slovak Republic; 192 Department of Subnuclear Physics, Institute of Experimental Physics of the Slovak Academy of Sciences, Kosice, Slovak Republic; 193 Department of Physics, University of Cape Town, Cape Town, South Africa; 194 Department of Physics, University of Johannesburg, Johannesburg, South Africa; 195 School of Physics, University of the Witwatersrand, Johannesburg, South Africa; 196 Department of Physics, Stockholm University, Stockholm, Sweden; 197 The Oskar Klein Centre, Stockholm, Sweden; 198 Physics Department, Royal Institute of Technology, Stockholm, Sweden; 199 Departments of Physics and Astronomy and Chemistry, Stony Brook University, Stony Brook, NY USA; 200 Department of Physics and Astronomy, University of Sussex, Brighton, UK; 201 School of Physics, University of Sydney, Sydney, Australia; 202 Institute of Physics, Academia Sinica, Taipei, Taiwan; 203 Department of Physics, Technion: Israel Institute of Technology, Haifa, Israel; 204 Raymond and Beverly Sackler School of Physics and Astronomy, Tel Aviv University, Tel Aviv, Israel; 205 Department of Physics, Aristotle University of Thessaloniki, Thessaloniki, Greece; 206 International Center for Elementary Particle Physics and Department of Physics, The University of Tokyo, Tokyo, Japan; 207 Graduate School of Science and Technology, Tokyo Metropolitan University, Tokyo, Japan; 208 Department of Physics, Tokyo Institute of Technology, Tokyo, Japan; 209 Tomsk State University, Tomsk, Russia Russia; 210 Department of Physics, University of Toronto, Toronto, ON Canada; 211 INFN-TIFPA, Povo, Italy; 212 University of Trento, Trento, Italy; 213 TRIUMF, Vancouver, BC Canada; 214 Department of Physics and Astronomy, York University, Toronto, ON Canada; 215 Faculty of Pure and Applied Sciences, and Center for Integrated Research in Fundamental Science and Engineering, University of Tsukuba, Tsukuba, Japan; 216 Department of Physics and Astronomy, Tufts University, Medford, MA USA; 217 Department of Physics and Astronomy, University of California Irvine, Irvine, CA USA; 218 INFN Gruppo Collegato di Udine, Sezione di Trieste, Udine, Italy; 219 ICTP, Trieste, Italy; 220 Dipartimento di Chimica, Fisica e Ambiente, Università di Udine, Udine, Italy; 221 Department of Physics and Astronomy, University of Uppsala, Uppsala, Sweden; 222 Department of Physics, University of Illinois, Urbana, IL USA; 223 Instituto de Fisica Corpuscular (IFIC) and Departamento de Fisica Atomica, Molecular y Nuclear and Departamento de Ingeniería Electrónica and Instituto de Microelectrónica de Barcelona (IMB-CNM), University of Valencia and CSIC, Valencia, Spain; 224 Department of Physics, University of British Columbia, Vancouver, BC Canada; 225 Department of Physics and Astronomy, University of Victoria, Victoria, BC Canada; 226 Department of Physics, University of Warwick, Coventry, UK; 227 Waseda University, Tokyo, Japan; 228 Department of Particle Physics, The Weizmann Institute of Science, Rehovot, Israel; 229 Department of Physics, University of Wisconsin, Madison, WI USA; 230 Fakultät für Physik und Astronomie, Julius-Maximilians-Universität, Würzburg, Germany; 231 Fakultät für Mathematik und Naturwissenschaften, Fachgruppe Physik, Bergische Universität Wuppertal, Wuppertal, Germany; 232 Department of Physics, Yale University, New Haven, CT USA; 233 Yerevan Physics Institute, Yerevan, Armenia; 234 Centre de Calcul de l’Institut National de Physique Nucléaire et de Physique des Particules (IN2P3), Villeurbanne, France; 235 CERN, 1211 Geneva 23, Switzerland

## Abstract

Measurements of the electroweak production of a *W* boson in association with two jets at high dijet invariant mass are performed using $\sqrt{s} =$ 7 and 8 $\text {TeV}$ proton–proton collision data produced by the Large Hadron Collider, corresponding respectively to 4.7 and 20.2 fb$^{-1}$ of integrated luminosity collected by the ATLAS detector. The measurements are sensitive to the production of a *W* boson via a triple-gauge-boson vertex and include both the fiducial and differential cross sections of the electroweak process.

## Introduction

The non-Abelian nature of the standard model (SM) electroweak theory predicts the self-interactions of the weak gauge bosons. These triple and quartic gauge-boson couplings provide a unique means to test for new fundamental interactions. The fusion of electroweak (EW) bosons is a particularly important process for measuring particle properties, such as the couplings of the Higgs boson, and for searching for new particles beyond the Standard Model [1–11]. In proton–proton (*pp*) collisions, a characteristic signature of these processes is the production of two high-momentum jets of hadrons at small angles with respect to the incoming proton beams [12]. Measurements of this vector-boson-fusion (VBF) topology have been performed in *W* [13], *Z* [14, 15] and Higgs [16] boson production, though the observation of purely electroweak processes in this topology has only been achieved in individual measurements of *Z*-boson production. This paper presents a precise measurement of electroweak *W*-boson production in the VBF topology, with a significance well above the standard for claiming observation, as well as differential cross section measurements and constraints on anomalous triple-gauge-boson couplings (aTGCs).

The production of a *W* boson in association with two or more jets ($Wjj$) is dominated by processes involving strong interactions (strong $Wjj$ or QCD $Wjj$). These processes have been extensively studied by experiments at the Large Hadron Collider (LHC) [17, 18] and the Tevatron collider [19, 20], motivating the development of precise perturbative predictions [21–33]. The large cross section for *W*-boson production provides greater sensitivity to the VBF topology and to the electroweak production of $Wjj$ (electroweak $Wjj$ or EW $Wjj$) than corresponding measurements of *Z*- or Higgs-boson production.

**Fig. 1:**
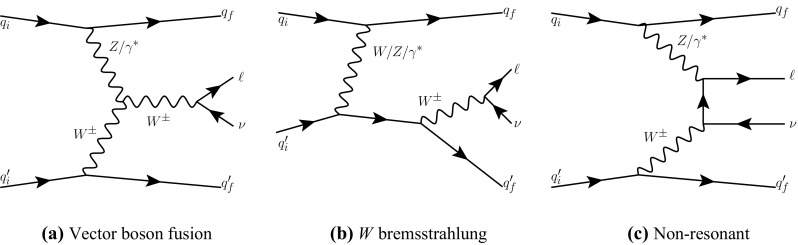
Representative leading-order diagrams for electroweak *Wjj* production at the LHC. In addition to **a** the vector boson fusion process, there are four **b** *W* bremsstrahlung diagrams, corresponding to $W^\pm$ boson radiation by any incoming or outgoing quark, and two **c** non-resonant diagrams, corresponding to $W^\pm$ boson radiation by either incoming quark

**Fig. 2:**
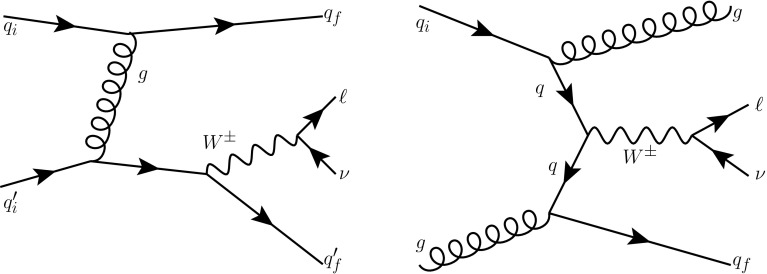
Examples of leading-order diagrams for strong *Wjj* production at the LHC. The *left-hand* diagram interferes with the electroweak diagrams of Fig. 1 when the final-state quarks have the same colours as the initial-state quarks

The VBF process is inseparable from other electroweak $Wjj$  processes, so it is not measured directly; sensitivity to the VBF production mechanism is quantified by determining constraints on operator coefficients in an effective Lagrangian approach [34]. The classes of electroweak diagrams constituting the signal are shown in Figure 1 [35] and contain at least three vertices where an electroweak gauge boson connects to a pair of fermions. Diboson production, where the final-state quarks result from the decay of an *s*-channel gauge boson, is not shown and is considered as a background; it is small for the VBF topology defined in the analysis. The large background from a *W* boson associated with strongly produced jets is shown in Fig. 2 and has only two electroweak vertices. This background has $\mathcal{{O}}$(10) times the yield of the signal process, and can interfere with the signal. This interference is suppressed because only a small subset of the background diagrams have the same initial and final state as the signal.

The analysis signature consists of a neutrino and either an electron or a muon, two jets with a high dijet invariant mass, and no additional jets at a wide angle from the beam. This signature discriminates signal events from the copious background events consisting of strongly produced jets associated with a *W* (or *Z*) boson, top-quark production, or multijet production. The purity of electroweak $Wjj$  production increases with increasing dijet invariant mass, increasing the sensitivity to anomalous triple-gauge-boson couplings.

Measurements of the inclusive and fiducial cross sections of electroweak $Wjj$  production in proton–proton collisions at centre-of-mass energies $\sqrt{s}=7$ and 8 $\text {TeV}$ are performed in a fiducial region with a signal-to-background ratio of approximately 1:8. The electroweak signal is extracted with a binned likelihood fit to the dijet invariant mass distribution. The fit determines the ratio $\mu _{\text {EW}}$ of the measured signal cross section to that of a Standard Model calculation [36]; this ratio is then multiplied by the prediction to provide the measured cross section. To reduce the uncertainties in the modelling of the strong $Wjj$  events, data are used to constrain their dijet mass distribution, resulting in a precise measurement of the electroweak $Wjj$ fiducial cross section. The quantum-mechanical interference between electroweak and strong $Wjj$ processes is not modelled and its impact on the measurement is estimated using a Monte Carlo simulation and taken as an uncertainty.

In order to explore the kinematics of the $Wjj$  topology, and the interplay between strong and electroweak production, the 8 $\text {TeV}$ data are unfolded differentially to particle level in many variables and phase-space regions, and compared to theoretical predictions. Electroweak $Wjj$  production is measured in regions where the signal purity is relatively high ($\gtrsim 10\%$); combined strong and electroweak $Wjj$  production is measured in the other regions. These measurements are then integrated to obtain fiducial cross sections in the different phase-space regions, albeit with larger uncertainties than the measurement with the constrained background.

Sensitivity to the VBF diagram is determined by modifying the triple-gauge-boson couplings. Anomalous couplings arising from new processes at a high energy scale would cause increasing deviations from the SM prediction for increasing momentum transfer between the incoming partons. Hence, a region of high momentum transfer is defined, and constraints on anomalous gauge couplings are set in the context of an effective field theory (EFT), including limits on interactions that violate charge-parity (CP) conservation.

The paper is organized as follows. The ATLAS detector and reconstruction of the final-state particles are described in Sect. 2. The definitions of the measurement phase-space regions and the event selection are given in Sect. 3. The modelling of signal and background processes is discussed in Sect. 4. Section 5 is dedicated to the precise extraction of the inclusive and fiducial cross sections, while Sect. 6 presents differential cross sections unfolded for detector effects. Section 7 describes limits on aTGCs and parameters of an effective field theory. Section 8 summarizes the results and the Appendix provides a comprehensive set of differential cross-section measurements.

## ATLAS detector and data reconstruction

The data set corresponds to LHC *pp* collisions at $\sqrt{s}=7$ $\text {TeV}$ in 2011 and at $\sqrt{s}=8$ $\text {TeV}$ in 2012, with final-state particles measured by the ATLAS detector. This section describes the detector and the reconstruction of the data to produce the final-state physics objects used in the measurements.

### ATLAS detector

ATLAS is a multi-purpose detector used to measure LHC particle collisions. A detailed description of the detector can be found in Ref. [37]. A tracking system comprises the inner detector (ID) surrounding the collision point, with silicon pixel and microstrip detectors most centrally located, followed by a transition radiation tracker at higher radii [38, 39]. These tracking detectors are used to measure the trajectories and momenta of charged particles up to pseudorapidities of $|\eta | = 2.5$.^1^ The ID is surrounded by a superconducting solenoid, providing a 2 T magnetic field for the tracking detectors.

A calorimeter system surrounds the solenoid magnet and consists of electromagnetic and hadronic sections. The electromagnetic section is segmented along the *z*-axis into a barrel region covering $|\eta | < 1.475$, two end-cap components spanning $1.375< |\eta | < 3.2$, and two forward components ($3.1< |\eta | < 4.9$). Similarly, the hadronic section comprises a barrel region ($|\eta | < 1.7$), two end-cap regions ($1.5< |\eta | < 3.2$), and two forward regions ($3.1< |\eta | < 4.9$). The barrel region of the hadronic section uses scintillator tiles as the active medium, while the remaining regions use liquid argon.

A muon spectrometer surrounds the calorimeter system and contains superconducting coils, drift tubes and cathode strip chambers to provide precise measurements of muon momenta within $|\eta | < 2.7$. The spectrometer also includes resistive-plate and thin-gap chambers to trigger on muons in the region $|\eta | < 2.4$.

The ATLAS trigger system uses three consecutive stages to select events for permanent storage. The first level uses custom electronics and the second level uses fast software algorithms to inspect regions of interest flagged by the first trigger level. At the third level, the full event is reconstructed using software algorithms similar to those used offline.

### Object reconstruction

Electrons, muons, and hadronic jets are reconstructed in the ATLAS detector. Each type of object has a distinctive signature and is identified using the criteria described below. The object identification includes track and vertex positions relative to the primary event vertex, defined as the reconstructed vertex with the highest summed $p_{\text {T}} ^2$ of all associated tracks. Each object is calibrated and modelled in Monte Carlo simulation, corrected to match data measurements of the trigger, reconstruction, and identification efficiencies, and of the energy and momentum scales and resolutions [40–44].

## Electrons

Electron candidates are reconstructed from energy clusters in the electromagnetic section of the calorimeter which are matched to tracks reconstructed in the ID. Candidates for signal events are required to satisfy ‘tight’ selection criteria [41, 42], which include requirements on calorimeter shower shape, track hit multiplicity, the ratio of reconstructed energy to track momentum, *E* / *p*, and the matching of the energy clusters to the track. In order to build templates to model the multijet background (see Sect. 4.2), a set of criteria is employed based on ‘loose’ or ‘medium’ selection, which drops the *E* / *p* requirement and uses less restrictive selection criteria for the other discriminating variables.

Electron candidates are required to be isolated to reject possible misidentified jets or heavy-flavour hadron decays. Isolation is calculated as the ratio of energy in an isolation cone around the primary track or calorimeter deposit to the energy of the candidate. Different isolation requirements are made in the 7 and 8 $\text {TeV}$ data sets, due to the different LHC and detector operating conditions. For 7 $\text {TeV}$ data taking, the requirements on track and calorimeter isolation variables associated with the electron candidate achieve a constant identification efficiency as a function of the candidate transverse energy ($E_{\text {T}}$) and pseudorapidity. The 8 $\text {TeV}$ trigger includes a requirement on track isolation, so the selection is more restrictive and requires the summed $p_{\text {T}}$ of surrounding tracks to be $<5\%$ of the electron candidate $E_{\text {T}}$, excluding the electron track and using a cone of size $R \equiv \sqrt{(\Delta \phi )^2 + (\Delta \eta )^2} = 0.2$ around the shower centroid.

## Muons

Muon candidates are identified as reconstructed tracks in the muon spectrometer which are matched to and combined with ID tracks to form a ‘combined’ muon candidate [43]. Quality requirements on the ID track include a minimum number of hits in each subdetector to ensure good track reconstruction. Candidates in 7 $\text {TeV}$ data are selected using a track-based fractional isolation requiring the scalar sum of the $p_{\text {T}}$ values of tracks within a cone of size $R = 0.2$ of the muon track to be less than 10% of the candidate $p_{\text {T}}$. For 8 $\text {TeV}$ data taking, requirements are applied to track and calorimeter fractional isolation using a cone of size $R = 0.3$. The upper bound on each type of isolation increases with increasing muon $p_{\text {T}}$, and is 15% for $p_{\text {T}} >30$ $\text {GeV}$.

Additional transverse ($d_0$) and longitudinal ($z_0$) impact parameter requirements of $|d_0/\sigma _{d_0}|<3$ (where $\sigma _{d_0}$ is the $d_0$ uncertainty) and $|z_0\sin \theta |<0.5$ mm are imposed on all muon and electron candidates to suppress contributions from hadron decays to leptons.

## Jets

Jets are reconstructed using the anti-$k_t$ algorithm [45] with a jet-radius parameter of 0.4, from three-dimensional clustered energy deposits in the calorimeters [46]. Jets are required to have $p_{\text {T}} >30$ $\text {GeV}$ and $|\eta |<4.4$, and must be separated from the lepton in $\eta$–$\phi$ space, $\Delta R (\ell , j) \ge 0.3$. Quality requirements are imposed to remove events where jets are associated with noisy calorimeter cells. Jet energies are corrected for the presence of low-energy contributions from additional in-time or out-of-time collisions (pile-up), the non-compensating response of the calorimeter, detector material variations, and energy losses in uninstrumented regions. This calibration is performed in bins of $p_{\text {T}}$ and $\eta$, using correction factors determined using a combination of Monte Carlo simulations and in-situ calibrations with data [44, 47]. The systematic uncertainties in these correction factors are determined from the same control samples in data. A significant source of uncertainty in this analysis arises from the modelling of the $\eta$ dependence of the jet energy response.

To suppress the contribution of jets from additional coincident *pp* collisions, the jet vertex fraction (JVF) [48] is used to reject central jets ($|\eta |<2.4$) that are not compatible with originating from the primary vertex. The JVF is defined as the scalar sum of the $p_{\text {T}}$ values of tracks associated with both the primary vertex and the jet, divided by the summed $p_{\text {T}}$ of all tracks associated with the jet. For the 7 $\text {TeV}$ data taking, the requirement is $|\mathrm {JVF}|\ge 0.75$; this requirement is loosened in 8 $\text {TeV}$ data taking to $|\mathrm {JVF}|\ge 0.5$ if the jet has $p_{\text {T}} <50$ $\text {GeV}$. The relaxed requirement in 8 $\text {TeV}$ data is due to the larger pile-up rate causing signal events to be rejected when using the 7 $\text {TeV}$ selection, and the requirement of $|\eta |<2.4$ is to ensure the jets are within the ID tracking acceptance.

Jets that are consistent with originating from heavy-flavour quarks are identified using a neural network algorithm trained on input variables related to the impact parameter significance of tracks in the jet and the secondary vertices reconstructed from these tracks [49]. Jets are identified as *b*-jets with a selection on the output of the neural network corresponding to an identification efficiency of 80%.

## Missing transverse momentum

In events with a leptonically decaying *W* boson, one expects large missing momentum in the transverse plane due to the escaping neutrino. The magnitude of this missing transverse momentum ($E_{\text {T}}^{\text {miss}}$) is constructed from the vector sum of muon momenta and three-dimensional energy clusters in the calorimeter [50, 51]. The clusters are corrected to account for the different response to hadrons compared to electrons or photons, as well as dead material and out-of-cluster energy losses. Additional tracking information is used to extrapolate low-momentum particles to the primary vertex to reduce the contribution from pile-up.

## Event selection

The proton–proton collision data samples correspond to a total integrated luminosity of 4.7 fb${}^{-1}$ for the 7 $\text {TeV}$ data and 20.2 fb${}^{-1}$ for the 8 $\text {TeV}$ data with uncertainties of 1.8% [52] and 1.9% [53], respectively.

The measurements use data collected with single-electron and single-muon triggers. The triggers identify candidate muons by combining an ID track with a muon-spectrometer track, and candidate electrons by matching an inner detector track to an energy cluster in the calorimeter consistent with an electromagnetic shower. The triggers in the 7 $\text {TeV}$ data require $p_{\text {T}} > 18$ $\text {GeV}$ for muons and either $E_{\text {T}} > 20$ $\text {GeV}$ or $E_{\text {T}} > 22$ $\text {GeV}$ for electrons, depending on the data-taking period. The 8 $\text {TeV}$ data events are selected by two triggers in each channel. The electron-channel triggers have $E_{\text {T}}$ thresholds of 24 and 60 $\text {GeV}$, where the lower-threshold trigger includes a calorimeter isolation criterion: the measured $E_{\text {T}}$ within a cone of radius $R=0.2$ around the electron candidate, excluding the electron candidate’s $E_{\text {T}}$, must be less than 10% of the $E_{\text {T}}$ of the electron. The muon-channel triggers have $p_{\text {T}}$ thresholds of 24 and 36 $\text {GeV}$. The lower-threshold trigger has a track-isolation requirement, where the scalar summed $p_{\text {T}}$ of tracks within a cone of radius $R = 0.2$ around the muon is required to be less than 12% of the $p_{\text {T}}$ of the muon.

The analysis defines many measurement regions varying in electroweak $Wjj$ purity. Table 1 shows the regions at the generated particle level based on the variables defined below. Particle-level objects are reconstructed as follows: jets are reconstructed using the anti-$k_t$ algorithm with a radius parameter of 0.4 using final-state particles with a proper lifetime longer than 10 ps; and leptons are reconstructed by combining the final-state lepton with photons within a cone of $R=0.1$ around the lepton. The requirements in Table 1 are also used to select data events, except for the following differences: (1) electrons must have $|\eta |<2.47$ and cannot be in the crack region of the calorimeter ($1.37< |\eta | < 1.52$); (2) muons must have $|\eta |<2.4$; and (3) jets are selected using pseudorapidity ($|\eta |<4.4$) rather than rapidity. Also, a *b*-jet veto is applied to the validation region in data when performing the measurement of the fiducial electroweak $Wjj$ cross section described in Sect. 5.

**Table 1:** Phase-space definitions at the generated particle level. Each phase-space region includes the preselection and the additional requirements listed for that region. The variables are defined in Sects. 3.1 and 3.2

Region name	Requirements
Preselection	Lepton $p_{\text {T}} > 25$ $\text {GeV}$
	Lepton $|\eta |<2.5$
	$E_{\text {T}}^{\text {miss}} > 25$ $\text {GeV}$
	$m_{\text{ T }} > 40$ $\text {GeV}$
	$p_{\text {T}} ^{j_1} > 80$ $\text {GeV}$
	$p_{\text {T}} ^{j_2} > 60$ $\text {GeV}$
	Jet $|y|<4.4$
	$M_{jj} > 500$ $\text {GeV}$
	$$\Delta y(j_1,j_2) >2$$
	$$\Delta R(j,\ell )>0.3$$
Fiducial and differential measurements	
Signal region	$N_{\text {lepton}}^{\text {cen}} = 1, N_{\text {jets}}^{\text {cen}} = 0$
Forward-lepton control region	$N_{\text {lepton}}^{\text {cen}} = 0, N_{\text {jets}}^{\text {cen}} = 0$
Central-jet validation region	$N_{\text {lepton}}^{\text {cen}} = 1, N_{\text {jets}}^{\text {cen}} \ge 1$
Differential measurements only	
Inclusive regions	$M_{jj} > 0.5$ $\text {TeV}$, 1 $\text {TeV}$, 1.5 $\text {TeV}$, or 2 $\text {TeV}$
Forward-lepton/central-jet region	$N_{\text {lepton}}^{\text {cen}} = 0, N_{\text {jets}}^{\text {cen}} \ge 1$
High-mass signal region	$M_{jj} > 1$ $\text {TeV}$, $N_{\text {lepton}}^{\text {cen}} = 1, N_{\text {jets}}^{\text {cen}} = 0$
Anomalous coupling measurements only	
High-$q^2$ region	$M_{jj} > 1$ $\text {TeV}$, $N_{\text {lepton}}^{\text {cen}} = 1, N_{\text {jets}}^{\text {cen}} = 0$, $p_{\text {T}} ^{j_1} > 600$ $\text {GeV}$

### Event preselection

Signal candidate events are initially defined by the presence of missing transverse momentum ($E_{\text {T}}^{\text {miss}} >20$ $\text {GeV}$), exactly one charged lepton (electron or muon) candidate with $p_{\text {T}} > 25$ $\text {GeV}$, and at least two jets. The highest-$p_{\text {T}}$ jet is required to have $p_{\text {T}} ^{j_1} >80$ $\text {GeV}$ and the second jet must have $p_{\text {T}} ^{j_2} >60$ $\text {GeV}$. To isolate events with a *W* boson, a veto is imposed on events with a second same-flavour lepton with $p_{\text {T}} > 20$ $\text {GeV}$; these leptons are identified in data using relaxed isolation and impact parameter criteria. A minimum cut on the transverse mass, $m_{\text{ T }} >40$ $\text {GeV}$, of the *W*-boson candidate is additionally imposed, where $m_{\text{ T }}$ is defined by:

$$\begin{aligned} m_{\text{ T }} = \sqrt{\,2p_{\text {T}} \cdot E_{\text {T}}^{\text {miss}} \left[ 1-\cos \Delta \phi (\ell , E_{\text {T}}^{\text {miss}})\right] }. \end{aligned}$$

Jets are selected in data if they have $|\eta |<4.4$ and $\Delta R(j,\ell )>0.3$. A VBF topology is selected by requiring the invariant mass of the dijet system defined by the two highest-$p_{\text {T}}$ jets to satisfy $M_{jj} >500$ $\text {GeV}$, and the absolute value of the rapidity separation of the jets to satisfy $\Delta y(j_1,j_2) > 2$.

### Definitions of the measurement regions

**Fig. 3:**
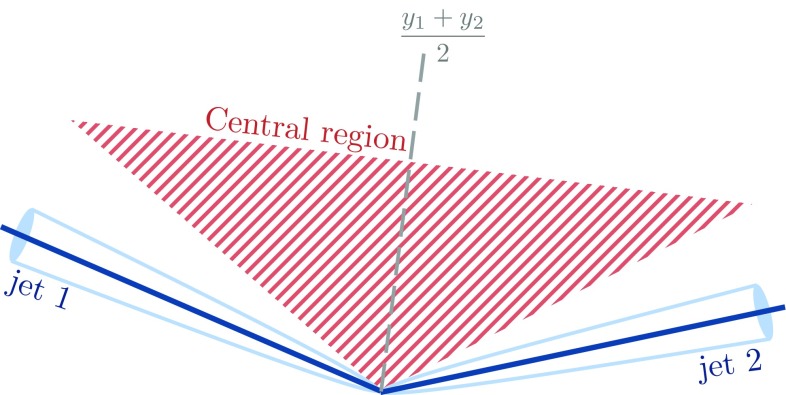
Illustration of the central region used to count leptons and jets in the definition of the signal, control, and validation regions. The rapidity range of the region corresponds to $C_{\text {max}}=0.4$ in Eq. (2). An object in the direction of the dashed line has $C = 0$

The above preselection defines an *inclusive* fiducial region, which is then split into four orthogonal fiducial regions defined by the presence or absence of the lepton or an additional jet in a “central” rapidity range between the two highest-$p_{\text {T}}$ jets. The signal EW $Wjj$ process is characterized by a lepton and no jets in the central rapidity range. This range is determined by the centrality variable $C_{\ell }$ or $C_j$ for the lepton or jets respectively:

1 $$\begin{aligned} C_{\ell ~(j)} \equiv \left| \frac{y_{\ell ~(j)} - \frac{y_1+y_2}{2}}{y_1-y_2}\right| , \end{aligned}$$

where $y_{\ell ~(j)}$ is the rapidity of the candidate lepton (jet), and $y_1$ and $y_2$ are the rapidities of the highest-$p_{\text {T}}$ (leading) and next-highest-$p_{\text {T}}$ (subleading) jets. Requiring the centrality to be below a value $C_{\text {max}}$ defines the selection of a rapidity range centred on the mean rapidity of the leading jets, i.e.,

2 $$\begin{aligned} \left[ \frac{y_1+y_2}{2}-C_{\text {max}}\times |y_1-y_2|,\quad \frac{y_1+y_2}{2}+C_{\text {max}}\times |y_1-y_2|\right] , \end{aligned}$$

as illustrated in Fig. 3. For $C_{\text {max}}=0.5$, the interval spans the entire rapidity region between the two jets; the number of jets within this interval is denoted $N_{\text {jets}}^{\text {gap}}$. In defining the electroweak $Wjj$ signal region, $C_{\text {max}}=0.4$ is used to count the number of leptons ($N_{\text {lepton}}^{\text {cen}}$) or jets ($N_{\text {jets}}^{\text {cen}}$) within the range. A value of $C_{\text {max}}=0.4$ permits an event with the emission of an additional jet close to one of the two highest-$p_{\text {T}}$ jets to be retained as a candidate signal event.

**Fig. 4:**
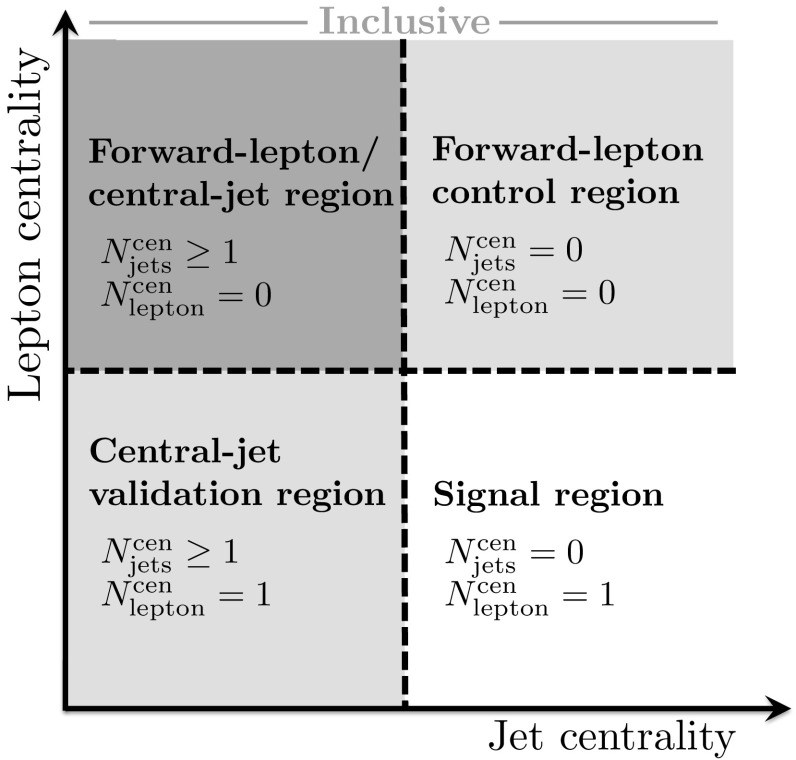
Illustration of the relationship between the signal, control, and validation fiducial regions. The signal region is defined by both a veto on additional jets (beyond the two highest-$p_{\text {T}}$ jets) and the presence of a lepton in the rapidity region defined in Eq. (2). The signal region is studied with either $M_{jj} >0.5$ $\text {TeV}$ or 1 $\text {TeV}$. A forward-lepton/central-jet fiducial region is also defined, for which the centrality requirements on the jets and the lepton are inverted with respect to the signal region. The inclusive region corresponds to the union of all four regions, and is studied with $M_{jj} >0.5,~1.0,~1.5,$ or 2.0 TeV. The quantities $N_{\text {jets}}^{\text {cen}}$ and $N_{\text {lepton}}^{\text {cen}}$ refer to the number of reconstructed leptons and additional jets reconstructed in the rapidity interval defined by Eq. (2) and illustrated in Fig. 3, with $C_{\text {max}} = 0.4$

The fiducial regions are illustrated in Fig. 4. The signal process is characterized by a *W* boson in the rapidity range spanned by the two jets (Fig. 1), with no jets in this range due to the absence of colour flow between the interacting partons. An event is therefore defined as being in the electroweak-enhanced *signal* region if the identified lepton is reconstructed in the rapidity region defined by Eq. (2) and no additional jets are reconstructed in this interval. A QCD-enhanced *forward-lepton control* fiducial region is defined by the requirement that neither the identified lepton nor any additional jets be present in the central rapidity interval. A second QCD-enhanced *central-jet validation* region is defined by events having both the identified lepton and at least one additional jet reconstructed in the central rapidity interval. These three orthogonal fiducial regions are used in Sect. 5 to extract the EW$Wjj$ production cross section, constrain the modelling of QCD $Wjj$production from data, and validate the QCD $Wjj$ modelling, respectively.

For the determination of unfolded differential cross sections presented in Sect. 6, four additional fiducial regions are studied: the inclusive region for the progressively more restrictive dijet invariant mass thresholds of 1.0, 1.5, and 2.0 $\text {TeV}$, and an orthogonal *forward-lepton/central-jet* region defined by events with the lepton outside the central region, but at least one additional jet reconstructed in the interval. For the study of EW $Wjj$ differential cross sections, the signal fiducial region with an increased dijet invariant mass requirement of $M_{jj} >1$ $\text {TeV}$ (*high-mass signal region*) is also analyzed; a further requirement that the leading-jet $p_{\text {T}}$ be greater than 600 $\text {GeV}$ defines a *high-* $q^2$ region used for constraints on aTGCs (discussed in Sect. 7).

## Modelling of signal and background processes

Simulated Monte Carlo (MC) samples are used to model $Wjj$ production, with small data-derived corrections applied to reduce systematic uncertainties. Other processes producing a prompt charged lepton are also modelled with MC samples. The multijet background, where a photon or hadronic jet is misreconstructed as a prompt lepton, or where a lepton is produced in a hadron decay, is modelled using data.

### Monte Carlo simulation

The measurements described in this paper focus on the electroweak production of $Wjj$. This process has different kinematic properties to strong $Wjj$ production, but there is nonetheless some small interference between the processes. The other significant background processes are top-quark, *Z*-boson, and diboson production, which are modelled with MC simulation. All MC samples used to model the data are passed through a detector simulation [54] based on geant4  [55]. Pile-up interactions are modelled with Pythia8 (v. 8.165) [56]. Table 2 lists the MC samples and the cross sections used in the MC normalization.

**Table 2:** Monte Carlo samples used to model the signal and background processes. The cross sections times branching fractions, $\sigma \cdot \mathcal {B}$, are quoted for $\sqrt{s} = 7$ and 8 $\text {TeV}$. The branching fraction corresponds to the decay to a single lepton flavour, and here $\ell$ refers to *e*, $\mu$, or $\tau$. The neutral current $Z/\gamma ^*$ process is denoted by *Z*. To remove overlap between $W(\rightarrow \tau \nu )$ + 2 jets and *WW*/*WZ* in 7 $\text {TeV}$ samples, events with a generated $\tau$ lepton are removed from the 7 $\text {TeV}$ *WW*/*WZ* samples. Jets refer to a quark or gluon in the final state of the matrix-element calculation

Process	MC generator	$\sigma \cdot \mathcal {B}$ [pb]	
		7 $\text {TeV}$	8 $\text {TeV}$
$W(\rightarrow e\nu , \mu \nu )$ + 2 jets			
2 EW vertices	Powheg + Pythia8	4670	5340
4 EW vertices (no dibosons)	Powheg + Pythia8	2.7	3.4
$W(\rightarrow \tau \nu )$ inclusive			
2 EW vertices	Sherpa	10100	11900
$W(\rightarrow \tau \nu )$ + 2 jets			
4 EW vertices (with dibosons)	Sherpa	8.4	
4 EW vertices (no dibosons)	Sherpa		4.2
Top quarks			
$t{\bar{t}}(\rightarrow \ell \nu b\bar{q}q\bar{b}, \ell \nu b\ell \nu \bar{b})$	mc@nlo + Herwig	90.0	
	Powheg + Pythia6		114
*tW*	AcerMC + Pythia6	15.3	
	mc@nlo + Herwig		20.7
$t\bar{b}q \rightarrow \ell \nu b\bar{b}q$	AcerMC + Pythia6	23.5	25.8
$t\bar{b} \rightarrow \ell \nu b\bar{b}$	AcerMC + Pythia6	1.0	
	mc@nlo + Herwig		1.7
$Z(\rightarrow \ell \ell )$ inclusive, $m_{\ell \ell }>40$ $\text {GeV}$			
2 EW vertices	Sherpa	3140	3620
$Z(\rightarrow ee,\mu \mu )$ + 2 jets, $m_{ee,\mu \mu } >40$ $\text {GeV}$			
4 EW vertices (no dibosons)	Sherpa	0.7	0.9
Dibosons			
*WW*	Herwig++	45.9	56.8
*WZ*	Herwig++	18.4	22.5
*ZZ*	Herwig++	6.0	7.2

## $Wjj$

The primary model of the signal and background $Wjj$ processes in the analysis is the next-to-leading-order (NLO) Powheg Monte Carlo generator [29, 36, 57, 58], interfaced with Pythia8 using the AU2 parameter values [59] for the simulation of parton showering, underlying event, and hadronization. Two final-state partons with $p_{\text {T}} >20$ $\text {GeV}$ are required for the signal. A generator-level suppression is applied in the background generation to enhance events with one parton with $p_{\text {T}} > 80$ $\text {GeV}$ and a second parton with $p_{\text {T}} > 60$ $\text {GeV}$, and the mass of the pair larger than 500 $\text {GeV}$. Parton momentum distributions are modelled using the CT10 [60] set of parton distribution functions (PDFs). The QCD factorization and renormalization scales are set to the *W*-boson mass for the sample with jets produced via the electroweak interaction. For the sample with strongly produced jets, the hard-process scale is also the *W*-boson mass while the QCD emission scales are set with the multiscale-improved NLO (MiNLO) procedure [61] to improve the modelling and reduce the scale dependence. Uncertainties due to missing higher-order contributions are estimated by doubling and halving the factorization and renormalization scales independently, but keeping their ratio within the range 0.5–2.0. Uncertainties due to parton distribution functions are estimated using CT10 eigenvector variations rescaled to 68% confidence level, and an uncertainty due to the parton shower and hadronization model is taken from the difference between predictions using the Pythia8 and Herwig++  [62, 63] generators.

Measured particle-level differential distributions are also compared to the Sherpa (v. 1.4) [64] generation of QCD+EW $Wjj$ production at leading-order accuracy, including interference. An uncertainty due to the neglect of interference in the EW $Wjj$ measurement is estimated using this sample and individual Sherpa QCD and EW $Wjj$ samples. The individual samples are also used to model the small contribution from $W\rightarrow \tau \nu$ decays. Measured distributions of QCD+EW $Wjj$ production are compared to the combined QCD+EW and to the QCD $Wjj$ samples, the latter to demonstrate the effect of the EW $Wjj$ process. The QCD $Wjj$ sample is a $W+(n)$-parton prediction with $n\le 4$ partons with $p_{\text {T}} > 15$ $\text {GeV}$ produced via QCD interactions. The EW $Wjj$ sample has two partons produced via electroweak vertices, and up to one additional parton produced by QCD interactions. The CKKW matching scheme [65] is used to remove the overlap between different parton multiplicities at the matrix-element level. The predictions use the CT10 PDFs and the default parameter values for simulating the underlying event. Renormalization and factorization scales are set using the standard dynamical scale scheme in Sherpa. The interference uncertainty is cross-checked with the Madgraph  [28] generator interfaced to Pythia8.

For unfolded distributions with a low purity of electroweak $Wjj$ production, an additional comparison is made to the all-order resummation calculation of hej (High Energy Jets) [33] for strong $Wjj$ production. The calculation improves the accuracy of predictions in wide-angle or high-invariant-mass dijet configurations, where logarithmic corrections are significant. To allow a comparison to unfolded data and to other generators, the small electroweak $Wjj$ contribution is added using Powheg interfaced to Pythia8 and the sum is labelled hej (qcd) + pow+py (ew).

Both the Powheg and Sherpa predictions for electroweak $Wjj$ production omit the small contribution from diboson production processes, assuming negligible interference with these processes. Higher-order electroweak corrections to the background $Wjj$ process are studied with OpenLoops [66, 67] and found to affect the measured fiducial cross section by $<1\%$.

## Other processes

Background contributions from top-quark, $Z + 2\text { jets}$, and diboson processes are estimated using MC simulation.

The top-quark background consists of pair-production and single-production processes, with the latter including *s*-channel production and production in association with a *b* quark or *W* boson. Top-quark pair production is normalized using the cross section calculated at next-to-next-to-leading order (NNLO) in $\alpha _{\text {S}}$, with resummation to next-to-next-to-leading logarithm (NNLL) using TOP++2.0 [68]. Kinematic distributions are modelled at NLO using the mc@nlo  [69] generator and the Herwig  [63, 70] parton shower model for 7 $\text {TeV}$ data, and with Powheg and Pythia6 (v. 6.427) [71] for 8 $\text {TeV}$ data; both use the CT10 PDF set. An uncertainty due to the parton shower model, and its interface to the matrix-element generator, is estimated by comparing the Powheg sample to an mc@nlo sample interfaced to Herwig. Single-top-quark production in the *t*-channel, $t\bar{b}q \rightarrow \ell \nu b\bar{b}q$, is modelled using the leading-order generator AcerMC (v. 3.8) [72] interfaced with Pythia6 and the CTEQ6L1 [73] PDF set, and the sample is normalized using the cross sections calculated by the generator. Modelling of the *s*-channel production of a single top quark, $t\bar{b} \rightarrow \ell \nu b\bar{b}$, and of the associated production of a top quark and a *W* boson are performed using AcerMC with Pythia6 in 7 $\text {TeV}$ data and mc@nlo with Herwig in 8 $\text {TeV}$ data. These samples are also normalized using the generator cross-section values.

Background from the $Z + 2\text { jets}$ (*Zjj*) process, which contributes when one of the leptons is not reconstructed and the $E_{\text {T}}^{\text {miss}}$ is large, is modelled using Sherpa and the CT10 PDF set. For the background with jets from QCD radiation, an inclusive Drell–Yan sample is produced at NLO [74] and merged with the leading-order (LO) production of additional partons (up to five). The background with jets produced purely through the electroweak interaction is modelled at leading order. This combination of samples is also used to model the $W(\rightarrow \tau \nu )$ + 2 jets background; the 7 $\text {TeV}$ sample includes *WW* and *WZ* production. The interference between the electroweak and QCD production of jets for these small backgrounds has a negligible impact on the measurements and is not modelled.

The diboson background processes $WW/WZ\rightarrow \ell \nu q\bar{q}^{(')}$ and $ZZ\rightarrow \ell \ell q\bar{q}$ provide only a small contribution at high dijet mass since the distribution peaks at the mass of the *W* or *Z* boson. The interference between the single and pair production of electroweak bosons is negligible for the mass range selected by the analysis. The diboson processes are modelled at leading order with Herwig++ and normalized to the NLO cross section [75]. The generation uses the CTEQ6L1 PDF set. In 7 $\text {TeV}$ samples, $W\rightarrow \tau \nu$ decays are removed since they are included in the $Wjj$ samples.

### Multijet background

Multijet production constitutes a background to the $Wjj$ process when one of the jets is misidentified as a lepton and significant $E_{\text {T}}^{\text {miss}}$ arises from either a momentum mismeasurement or the loss of particles outside the detector acceptance. Due to the very small fraction of multijet events with both of these properties, and their relatively poor modelling in simulation, a purely data-driven method is used to estimate this background. The method inverts certain lepton identification criteria (described below) to obtain a multijet-dominated sample for modelling kinematic distributions. The $E_{\text {T}}^{\text {miss}}$ distribution is then fit to obtain a multijet normalization factor; this fit is performed separately in the signal, control, and validation regions. Systematic uncertainties are estimated by modifying the fit distribution and the identification criteria, and by propagating detector and theoretical uncertainties.

Modifications to the lepton identification criteria which enhance the multijet contribution are based on isolation and either the impact parameter with respect to the primary vertex (for muons) or the shower and track properties (for electrons). For the 7 TeV analysis, the impact parameter significance requirement is inverted in the muon channel ($|d_0|/\sigma _{d_0} > 3$). This preferentially selects muons from heavy-flavour hadron decays, a dominant source of muons in multijet events. For the 8 TeV analysis, no requirement on impact parameter significance is made and instead a track isolation requirement is applied orthogonal to the requirement for selected muons ($0.15< \sum p_{\text {T}} ^{R=0.3}/ p_{\text {T}} < 0.35$).

For the electron channel in $\sqrt{s}=7$ TeV data, triggers requiring a loose electron candidate are used to obtain a multijet modelling sample. The electron candidate must satisfy medium criteria on track hit multiplicity and track–shower matching in $\eta$, but must fail to satisfy at least one of the tight shower-based criteria. It also must not be isolated in the calorimeter: $\sum E_{\text {T}} ^{R=0.3}/ E_{\text {T}} > 0.2$. In $\sqrt{s}=8$ TeV data, electron candidates must satisfy medium selection criteria consistent with the trigger used in the analysis. As in the muon channel, a track isolation window is applied orthogonal to the requirement for selected electrons ($0.05< \sum p_{\text {T}} ^{R=0.2}/ p_{\text {T}} < 0.1$).

To normalize the multijet-dominated samples to the expected contribution with nominal lepton criteria, a fit to the $E_{\text {T}}^{\text {miss}}$ distribution is performed. The fit simultaneously determines the multijet and strong $Wjj$ normalizations in the region where the nominal lepton criteria are applied, taking the multijet distribution from the sample with inverted lepton identification criteria. Other contributions are fixed to their SM predictions, and the data are consistent with the post-fit distribution within uncertainties. The strong $Wjj$ normalization is consistent with that found in the fit to the dijet mass distribution described in Sect. 5.

Systematic uncertainties in the multijet normalization arise from uncertainties in the kinematic modelling and in jet, lepton, and $E_{\text {T}}^{\text {miss}}$ reconstruction. The modelling uncertainties dominate and are estimated using three methods: (1) modifying the lepton candidate selection for the kinematic distributions; (2) using $m_{\text{ T }}$ as an alternative fit distribution; and (3) varying the kinematic range of the fit. For each method, the largest change in the normalization is taken as a systematic uncertainty and added in quadrature with reconstruction and modelling uncertainties for processes modelled with Monte Carlo simulation. The leading uncertainty arises from the change in multijet normalization when fitting the $m_{\text{ T }}$ distribution instead of the $E_{\text {T}}^{\text {miss}}$ distribution. The next largest uncertainty results from variations of the isolation and impact parameter requirements in the lepton selection used for the kinematic distributions. The total relative systematic uncertainty of the multijet normalization in the muon (electron) channel is 28% (67%) for the $\sqrt{s}=7$ TeV analysis, and 36% (38%) for the $\sqrt{s}=8$ TeV analysis. The relatively large uncertainty in the $\sqrt{s}=7$ TeV electron channel results from a larger dependence on the fit distribution and range than in the other multijet fits.

### Distributions and yields

**Fig. 5:**
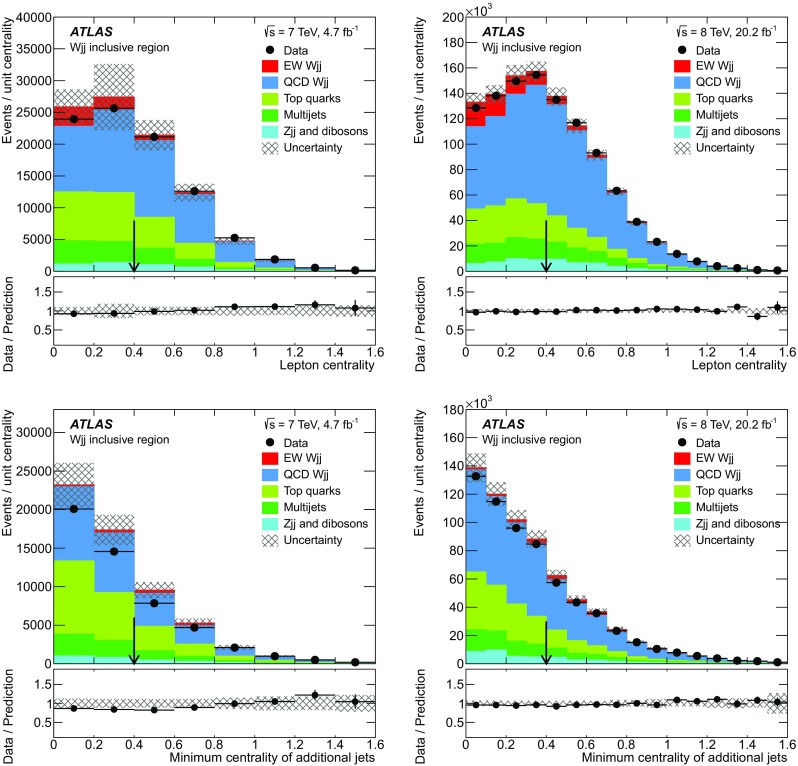
Predicted and observed distributions of the lepton centrality (*top*) and the minimum centrality of additional jets (*bottom*) for events in the inclusive fiducial region (i.e. after preselection) in 7 $\text {TeV}$ (*left*) and 8 $\text {TeV}$ (*right*) data. The *arrows* in the lepton-centrality distributions separate the signal-region selection (to the *left*) from the control-region selection (to the *right*). The *arrows* in the jet-centrality distributions separate the signal-region selection (to the *right*) from the validation-region selection (to the *left*). The *bottom panel* in each distribution shows the ratio of data to the prediction. The *shaded band* represents the statistical and experimental uncertainties summed in quadrature

**Fig. 6:**
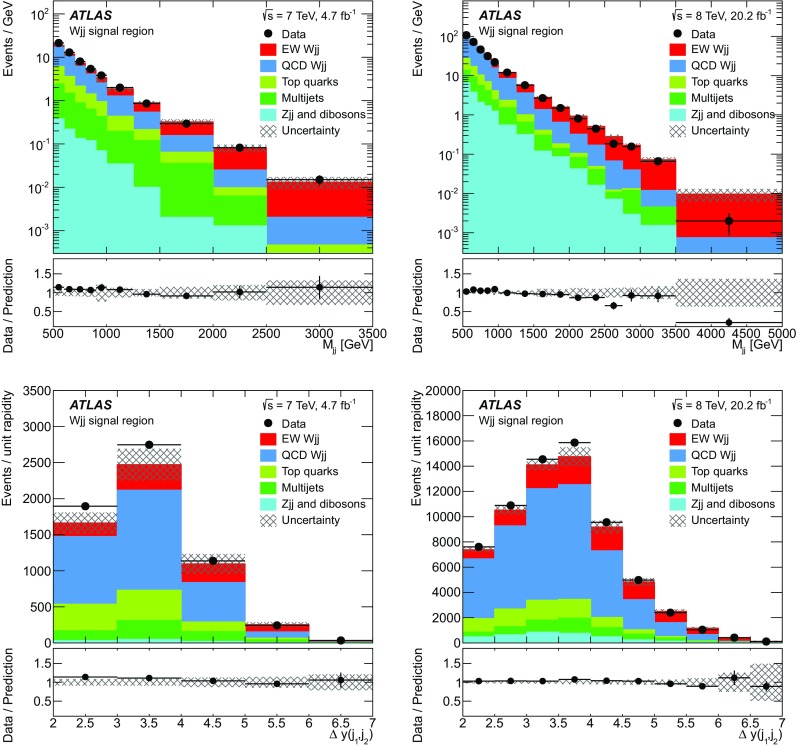
Predicted and observed distributions of the dijet invariant mass (*top*) and $\Delta y(j_1,j_2)$ (*bottom*) for events in the signal region in 7 $\text {TeV}$ (*left*) and 8 $\text {TeV}$ (*right*) data. The bottom panel in each distribution shows the ratio of data to the prediction. The shaded band represents the statistical and experimental uncertainties summed in quadrature

The distributions of lepton centrality and the minimum centrality of additional jets, which are used to separate signal, control, and validation regions, are shown in Fig. 5 for the 7 and 8 TeV data and the corresponding SM predictions after the preselection. The comparisons of the SM predictions to data show general agreement within the estimated uncertainties. The predictions include correction factors for lepton identification and triggering, and the bands correspond to the combination of statistical and experimental uncertainties. The signal-region dijet mass distributions, used to fit for the signal yield in the fiducial and total cross-section measurements, are shown in Fig. 6 for both data sets. The figure also shows the dijet rapidity difference, which is correlated with dijet mass and demonstrates an enhancement in signal at high values. Table 3 details the data and SM predictions for the individual processes in the signal region, and Table 4 shows the total predictions and the observed data in each of the fiducial regions defined in Sect. 3.

**Table 3:** Observed data and predicted SM event yields in the signal region. The MC predictions are normalized to the theoretical cross sections in Table 2. The relative uncertainty of the total SM prediction is $\mathcal{{O}}$(10%)

Process	7 TeV	8 TeV
$$Wjj $$ (EW)	920	5600
$$Wjj $$ (QCD)	3020	19,600
Multijets	500	2350
$t\bar{t}$	430	1960
Single top	244	1470
*Zjj* (QCD)	470	1140
Dibosons	126	272
*Zjj* (EW)	5	79
Total SM	5700	32,500
Data	6063	33,719

**Table 4:** Observed data and total predicted SM event yields in each measurement region. The MC predictions are normalized to the theoretical cross sections times branching ratios in Table 2. The relative uncertainty of the total SM prediction is $\mathcal{{O}}$(10%)

Region name	7 TeV		8 TeV	
	SM prediction	Data	SM prediction	Data
Fiducial and differential measurements				
Signal region	5700	6063	32500	33719
Forward-lepton control region	5000	5273	29400	30986
Central-jet validation region	2170	2187	12400	12677
Differential measurement only				
Inclusive region, $M_{jj} >500$ GeV	–	–	106000	107040
Inclusive region, $M_{jj} >1$ TeV	–	–	17400	16849
Inclusive region, $M_{jj} >1.5$ TeV	–	–	3900	3611
Inclusive region, $M_{jj} >2$ TeV	–	–	1040	890
Forward-lepton/central-jet region	–	–	12000	12267
High-mass signal region	–	–	6100	6052
Anomalous coupling measurements only				
High-$q^2$ region	–	–	39	30

## Fiducial and total electroweak $Wjj$ cross sections

The measurement of the fiducial EW $Wjj$ cross section in the signal region uses a control-region constraint to provide a precise determination of the electroweak production cross section for *W* bosons produced in association with dijets at high invariant mass. The measurement is performed with an extended joint binned likelihood fit [76] of the $M_{jj}$ distribution for the normalization factors of the QCD $Wjj$ and EW $Wjj$ Powheg + Pythia8 predictions, $\mu _{\text {QCD}}$ and $\mu _{\text {EW}}$ respectively, defined as follows:

$$\begin{aligned} (\sigma _i^{\ell \nu jj} \times \mathcal{{A}}_i)^\mathrm {meas}= & {} \mu _i \cdot (\sigma _i^{\ell \nu jj} \times \mathcal{{A}}_i)^\mathrm {theo} \\= & {} \frac{N_i}{\mathcal{{C}}_i \mathcal{{L}}}, \end{aligned}$$

where $\sigma ^{\ell \nu jj}_i$ is the cross section of process *i* (QCD $Wjj$ or EW $Wjj$ production in a single lepton channel), $\mathcal{{A}}_i$ is the acceptance for events to pass the signal selection at the particle level (see Table 1), $N_i$ is the number of measured events, $\mathcal{{L}}$ is the integrated luminosity, and $\mathcal{{C}}_i$ is the ratio of reconstructed to generated events passing the selection and accounts for experimental efficiencies and resolutions. The fit includes a Gaussian constraint for all non-$Wjj$ backgrounds, and accounts only for statistical uncertainties in the expected yield. The fit result for $\mu _{\text {EW}}$ is translated into a fiducial cross section by multiplying $\mu _{\text {EW}}$ by the predicted fiducial cross section from Powheg + Pythia8. In addition, the total cross section for jets with $p_{\text {T}} > 20$ $\text {GeV}$ is calculated by dividing the fiducial cross section by $\mathcal{{A}}$ for the EW $Wjj$ process.

The dijet mass provides the discriminating fit distribution. The region at relatively low invariant mass (${\approx }$500–1000 $\text {GeV}$) has low signal purity and primarily determines $\mu _{\text {QCD}}$, while events with higher invariant mass have higher signal purity and mainly determine $\mu _{\text {EW}}$. The interference between the processes is not included in the fit, and is instead taken as an uncertainty based on SM predictions.

The uncertainty in the shape of the QCD $Wjj$ distribution dominates the measurement, but is reduced by using the forward-lepton control region to correct the modelling of the $M_{jj}$ shape. This control region is defined in Table 1 and uses the same selection as the signal region, except for the inversion of the central-lepton requirement. This section describes the application of the control-region constraint, the uncertainties in the measurement, and the results of the fit.

### Control-region constraint

The SM prediction of the dijet mass distribution receives significant uncertainties from the experimental jet energy scale and resolution. These uncertainties are constrained with a correction to the predicted distribution derived using data in a control region where the signal contribution is suppressed. This forward-lepton control region is selected using the lepton centrality distribution. Residual uncertainties arise primarily from differences in the dijet mass spectrum between the control region and the signal region.

**Fig. 7:**
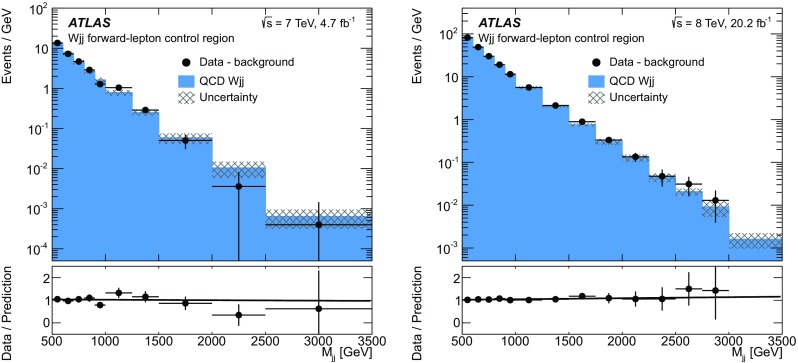
Comparison of the predicted QCD $Wjj$ dijet mass distribution to data with background processes subtracted, for events in the forward-lepton control region in 7 $\text {TeV}$ (*left*) and 8 $\text {TeV}$ (*right*) data. The *bottom panel* in each distribution shows the ratio of data to the QCD $Wjj$ prediction, and the result of a linear fit to the ratio. The *error bars* represent statistical and experimental uncertainties summed in quadrature

To derive the $M_{jj}$ correction, all processes other than strong $Wjj$ production are subtracted from the data and the result is compared to the prediction (Fig. 7). The correction is then determined with a linear fit to the ratio of the subtracted data to the $Wjj$ prediction. The slopes of the fits in 7 and 8 $\text {TeV}$ data are consistent with zero; they are $(0.2 \pm 1.1)$%/$\text {TeV}$ and $(0.28 \pm 0.43)$%/$\text {TeV}$, respectively, where the uncertainties are statistical only. The effect of a slope correction of 1%/$\text {TeV}$ is approximately 0.1 in the measured $\mu _{\text {EW}}$.

**Fig. 8:**
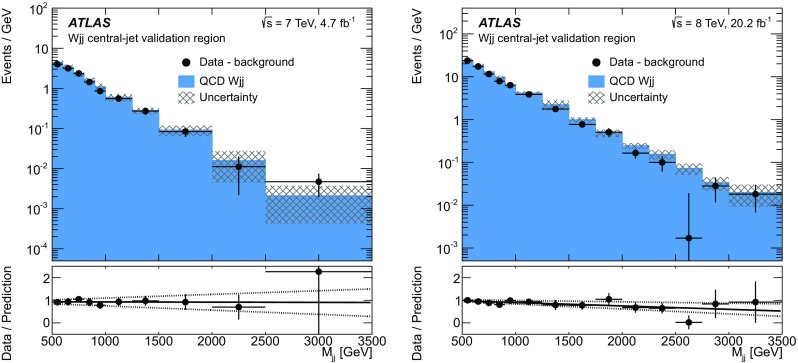
Comparison of the corrected QCD $Wjj$ background dijet mass distribution to data with background processes subtracted, for events in the central-jet validation region in 7 $\text {TeV}$ (*left*) and 8 $\text {TeV}$ (*right*) data. The *bottom panel* in each subfigure shows the ratio of data to prediction, and the result of a linear fit to the ratio (*solid line*). The *error bars* represent statistical and experimental uncertainties summed in quadrature. The *dotted lines* show the fit with slope adjusted up and down by statistical and experimental uncertainties

Systematic uncertainties in the corrected dijet mass distribution in the signal and validation regions are estimated by varying each source of uncertainty up or down by $1\sigma$ and calculating the corresponding slope correction in the control region in the simulation. This correction is applied to the prediction in the signal region and the fit performed on pseudodata derived from the nominal prediction. The resulting change in $\mu _{\text {EW}}$ is taken as the corresponding systematic uncertainty. The method is illustrated in the central-jet validation region in Fig. 8, where the background-subtracted and corrected $Wjj$ dijet mass distribution is compared to data. The ratio of subtracted data to the corrected $Wjj$ prediction is consistent with a line of zero slope when considering statistical and experimental uncertainties (the dotted lines in the figure).

### Uncertainties in $\mu _{\text {EW}}$

Uncertainties in $\mu _{\text {EW}}$ consist of: statistical uncertainties in the fit to the normalizations of the signal and background $Wjj$ processes in the signal region; the statistical uncertainty of the correction from the control region; and experimental and theoretical uncertainties affecting the signal and background predictions. Table 5 summarizes the uncertainties in the measurement of $\mu _{\text {EW}}$.

**Table 5:** The statistical and systematic uncertainty contributions to the measurements of $\mu _{\text {EW}}$ in 7 and 8 TeV data

Source	Uncertainty in $\mu _{\text {EW}}$	
	7 TeV	8 TeV
Statistical		
Signal region	0.094	0.028
Control region	0.127	0.044
Experimental		
Jet energy scale ($\eta$ intercalibration)	0.124	0.053
Jet energy scale and resolution (other)	0.096	0.059
Luminosity	0.018	0.019
Lepton and $E_{\text {T}}^{\text {miss}}$ reconstruction	0.021	0.012
Multijet background	0.064	0.019
Theoretical		
MC statistics (signal region)	0.027	0.026
MC statistics (control region)	0.029	0.019
EW $Wjj$ (scale and parton shower)	0.012	0.031
QCD $Wjj$ (scale and parton shower)	0.043	0.018
Interference (EW and QCD $Wjj$)	0.037	0.032
Parton distribution functions	0.053	0.052
Other background cross sections	0.002	0.002
EW $Wjj$ cross section	0.076	0.061
Total	0.26	0.14

The total statistical uncertainty in $\mu _{\text {EW}}$ of the joint likelihood fit is 0.16 (0.052) in 7 (8) $\text {TeV}$ data, where the leading uncertainty is the statistical uncertainty of the data in the control region rather than in the signal region.

Systematic uncertainties affecting the MC prediction are estimated by varying each uncertainty source up and down by $$1\sigma $$ in all MC processes, fitting the ratio of the varied QCD $$Wjj $$ prediction to the nominal prediction in the control region, and performing the signal region fit using the varied samples as pseudodata and the nominal samples as the templates. The largest change in $$\mu $$ from the up and down variations is taken as a symmetric uncertainty. The dominant experimental uncertainty in $$\mu _{\text {EW}} $$ is due to the calibration of the $$\eta $$ dependence of the jet energy scale, and is 0.124 (0.053) in 7 (8) $$\text {TeV}$$ data. Other uncertainties in the jet energy scale (JES) and resolution (JER) are of similar size when combined, with the largest contribution coming from the uncertainty in modelling the ratio of responses to quarks and gluons. Uncertainties due to multijet modelling are estimated by separately varying the normalization and distribution of the multijet background in each phase-space region and combining the effects in quadrature.

Theoretical uncertainties arise from the statistical uncertainty on the MC predictions; the lack of interference between signal and background $Wjj$ processes in the MC modelling; $Wjj$ renormalization and factorization scale variations and parton-shower modelling, which affect the acceptance of the jet centrality requirement; parton distribution functions; and cross-section uncertainties. The uncertainty due to MC statistics is 0.040 (0.032) in 7 (8) $\text {TeV}$ data. The interference uncertainty is estimated by including the Sherpa leading-order interference model as part of the background $Wjj$ process and affects the measurement of $\mu _{\text {EW}}$ by 0.037 (0.032) in 7 (8) $\text {TeV}$ data. Uncertainties due to PDFs are 0.053 (0.052) for 7 (8) $\text {TeV}$ data. Scale and parton-shower uncertainties are $\approx 0.04$ in both the 7 and 8 $\text {TeV}$ measurements. The scale uncertainty in EW $Wjj$ production is larger at $\sqrt{s}=8$ TeV than at 7 $\text {TeV}$ because of the increasing uncertainty with dijet mass and the higher mean dijet mass at 8 $\text {TeV}$. The scale uncertainty in QCD $Wjj$ production is larger at $\sqrt{s}=7$ $\text {TeV}$ because the data constraint has less statistical power than at 8 $\text {TeV}$.

Finally, a 0.076 (0.061) uncertainty in the signal cross section at 7 (8) $\text {TeV}$ due to higher-order QCD corrections and non-perturbative modelling is estimated using scale and parton-shower variations, affecting the measurement of $\mu _{\text {EW}}$ but not the extracted cross sections.

### Electroweak $Wjj$ cross-section results

**Fig. 9:**
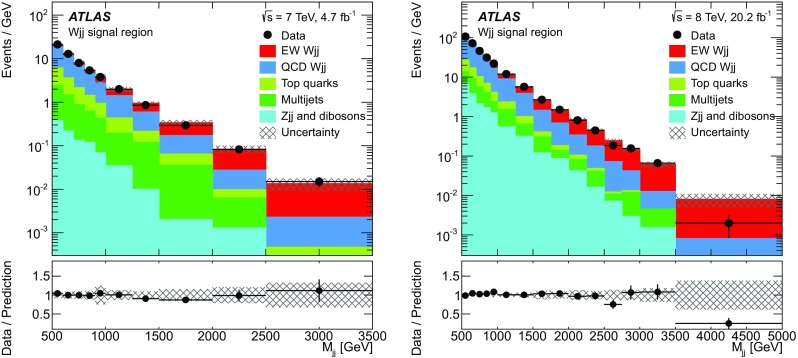
Distributions of the dijet invariant mass for events in the signal region in 7 $\text {TeV}$ (*left*) and 8 $\text {TeV}$ (*right*) data, after fitting for the yields of the individual $Wjj$ processes. The *bottom panel* in each distribution shows the ratio of data to predicted signal-plus-background yields. The *shaded band* centred at unity represents the statistical and experimental uncertainties summed in quadrature

The dijet mass distributions in 7 and 8 $\text {TeV}$ data after fitting for $\mu _{\text {EW}}$ and $\mu _{\text {QCD}}$ are shown in Fig. 9. There is good overall agreement between the normalized distributions and the data. The fit results for $\mu _{\text {QCD}}$ are $1.16 \pm 0.07$ for 7 $\text {TeV}$ data, and $1.09 \pm 0.05$ for 8 $\text {TeV}$ data. The measured values of $\mu _{\text {EW}}$ are consistent between electron and muon channels, with the following combined results:

$$\begin{aligned} \mu _{\text {EW}} ~(7~\mathrm {{\text {TeV}}})= & {} 1.00 \pm 0.16~\mathrm {(stat)}~\pm 0.17~\mathrm {(exp)}~\pm 0.12~\mathrm {(th)}, \\ \mu _{\text {EW}} ~(8~\mathrm {{\text {TeV}}})= & {} 0.81 \pm 0.05~\mathrm {(stat)}~\pm 0.09~\mathrm {(exp)}~\pm 0.10~\mathrm {(th)}. \end{aligned}$$

The measured value of $\mu _{\text {EW}}$ has a total uncertainty of 0.26 (0.14) in 7 (8) $\text {TeV}$ data, and differs from the SM prediction of unity by $<0.1\sigma$ ($1.4\sigma$). In the absence of a control region, the uncertainty would increase to 0.37 (0.18) in 7 (8) $\text {TeV}$ data.

The fiducial signal region is defined by the selection in Table 1 using particle-level quantities after parton showering. The measured and predicted cross sections times branching ratios in this region are shown in Table 6. The acceptance is calculated using Powheg + Pythia8 with a dominant uncertainty due to the parton-shower modelling which is estimated by taking the difference between Powheg + Pythia8 and Powheg + Herwig++. The uncertainty in the predicted fiducial cross section at $\sqrt{s} = 8$ $\text {TeV}$ includes a 4 fb contribution from scale variations and an 11 fb contribution from parton-shower modelling.

**Fig. 10:**
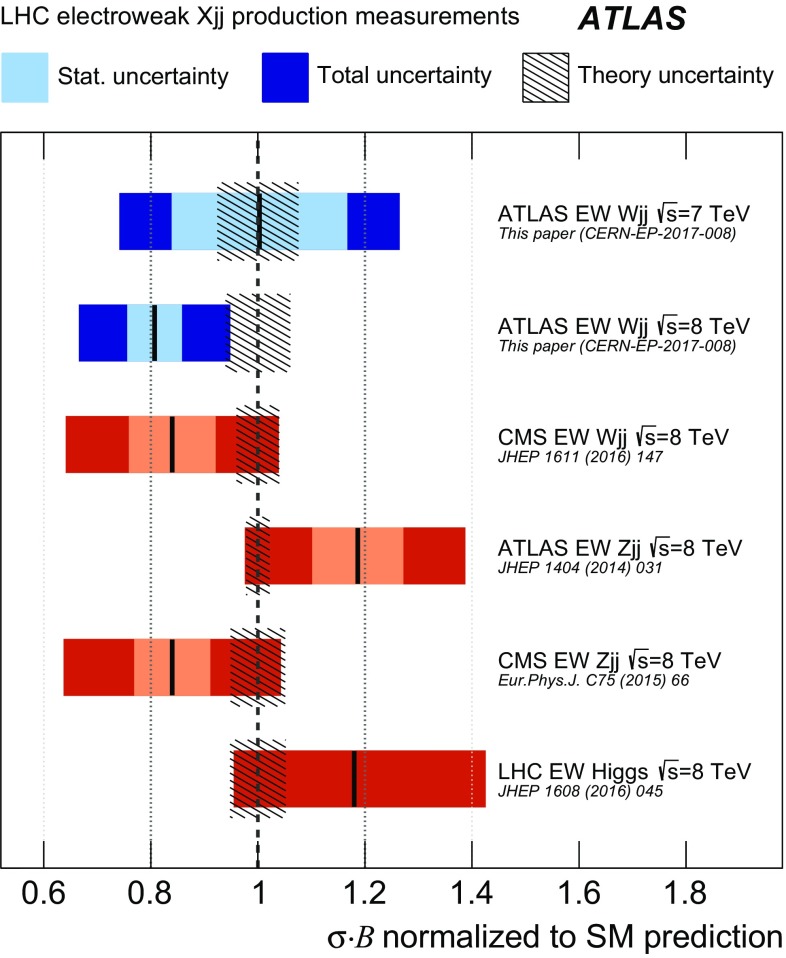
Measurements of the cross section times branching fractions of electroweak production of a single *W*, *Z*, or Higgs boson at high dijet invariant mass, divided by the SM predictions (Powheg +Pythia8 for ATLAS, Madgraph +Pythia8 for CMS, and Powheg +Pythia8 for the LHC combination). The *lighter shaded band* (where shown) represents the statistical uncertainty of the measurement, the *outer darker band* represents the total measurement uncertainty. Theoretical uncertainties in the SM prediction are represented by the *shaded region* centred at unity

A summary of this measurement and other measurements of boson production at high dijet invariant mass is shown in Fig. 10, normalized to SM predictions. The measurement with the smallest relative uncertainty is the 8 TeV $$Wjj $$ measurement presented here.

**Table 6:** Measured fiducial cross sections of electroweak $Wjj$ production in a single lepton channel, compared to predictions from Powheg + Pythia8. The acceptances and the inclusive measured production cross sections with $p_{\text {T}} > 20$ GeV jets are also shown

$\sqrt{s}$	$\sigma ^\mathrm {fid}_\mathrm {meas}$ [fb]	$\sigma ^\mathrm {fid}_\mathrm {SM}$ [fb]	Acceptance $\mathcal{{A}}$	$\sigma ^\mathrm {inc}_\mathrm {meas}$ [fb]
7 $\text {TeV}$	$144 \pm 23~\mathrm {(stat)}~\pm 23~\mathrm {(exp)}~\pm 13~\mathrm {(th)}$	$144 \pm 11$	$0.053 \pm 0.004$	$2760 \pm 670$
8 $\text {TeV}$	$159 \pm 10~\mathrm {(stat)}~\pm 17~\mathrm {(exp)}~\pm 15~\mathrm {(th)}$	$198 \pm 12$	$0.058 \pm 0.003$	$2890 \pm 510$

## Differential cross sections

Differential cross section measurements provide valuable information on the observed kinematic properties of a process, testing the theoretical predictions and providing model-independent results to probe for new physics. This section presents differential measurements in the $\sqrt{s}=8$ $\text {TeV}$ data that discriminate EW $Wjj$ from QCD $Wjj$ production, after first introducing the unfolding procedure, uncertainties, and the fiducial measurement regions. The large event yields allow more precise tests of these distributions than other VBF measurements and provide the most comprehensive tests of predictions in VBF-fiducial regions. Distributions sensitive to anomalous triple gauge couplings are also presented and extend to values of momentum transfer approaching 1 $\text {TeV}$, directly probing these energies for the presence of new interactions. Additional distributions are provided in Appendix A, and the complete set of measurements is available in hepdata [77].

All differential production cross sections are measured both as absolute cross sections and as distributions normalized by the cross section of the measured fiducial region ($\sigma ^\mathrm {fid}_W$). The normalizations are performed self-consistently, i.e. data measurements are normalized by the total fiducial data cross section and MC predictions are normalized by the corresponding MC cross section. Many sources of uncertainty are reduced for normalized distributions, allowing higher-precision tests of the modelling of the shape of the measured observables.

Unfolded differential cross-section measurements are performed for both QCD+EW $Wjj$ and EW $Wjj$ production and compared to theoretical predictions from the Powheg + Pythia8, Sherpa, and hej event generators, which are described in Sect. 4.1. The reported cross sections are for a single lepton flavour and are normalized by the width of the measured bin interval.

### Unfolding and uncertainties

The MC simulations are used to correct the cross sections for detector and event selection inefficiencies, and for the effect of detector resolutions. An implementation [78] of a Bayesian iterative unfolding technique [79] is used to perform these corrections. The unfolding is based on a response matrix from the simulated events which encodes bin-to-bin migrations between a particle-level differential distribution and the equivalent reconstruction-level distribution. The matrix gives transition probabilities from particle level to reconstruction level, and Bayes’ theorem is employed to calculate the inverse probabilities. These probabilities are used in conjunction with a prior particle-level signal distribution, which is taken from the Powheg + Pythia8 simulations, to unfold the background-subtracted reconstruction-level data distributions. After this first unfolding iteration the unfolded data distribution is used as the new prior and the process repeated for another iteration. The unfolding procedure is validated by unfolding the Sherpa simulation using the Powheg + Pythia8 response matrix. For all distributions the unfolded and initial particle-level Sherpa predictions agree within the unfolding uncertainty assigned. Bin boundaries in unfolded distributions are chosen to ensure that ${>}66\%$ of particle-level events remain within the same interval at reconstruction level.

The sources of uncertainty discussed in Sect. 5 are assessed for the unfolded differential production cross sections. Figures are shown with statistical uncertainties as inner bars and total uncertainties as the outer bars. Statistical uncertainties are estimated using pseudoexperiments, with correlations between bins determined using a bootstrap method [80]. The $W\rightarrow e\nu$ and $W\rightarrow \mu \nu$ channels are found to be statistically compatible, and are combined. Theoretical uncertainties include the effects of scale and PDF variations on the prior distribution and on the response matrix. For unfolding EW $Wjj$ production, additional theoretical uncertainties arise from modelling the QCD $Wjj$ contribution subtracted from the data, and from the neglect of interference between the strong and electroweak $Wjj$ processes. The interference uncertainty is estimated using the same procedure as for the fiducial measurement (Sect. 5), i.e. by adding the Sherpa interference model to the background prediction. The interference uncertainty is shown explicitly as a shaded area in each bin of the measured distributions. An uncertainty in the unfolding procedure is estimated by reweighting the simulation such that the distributions match the unfolded data, and then unfolding the data with the reweighted simulation; the change in the unfolded measurement is symmetrized and taken as an uncertainty. Experimental uncertainties are assessed by unfolding the data distributions using a modified response matrix and prior incorporating the change in detector response.

**Fig. 11:**
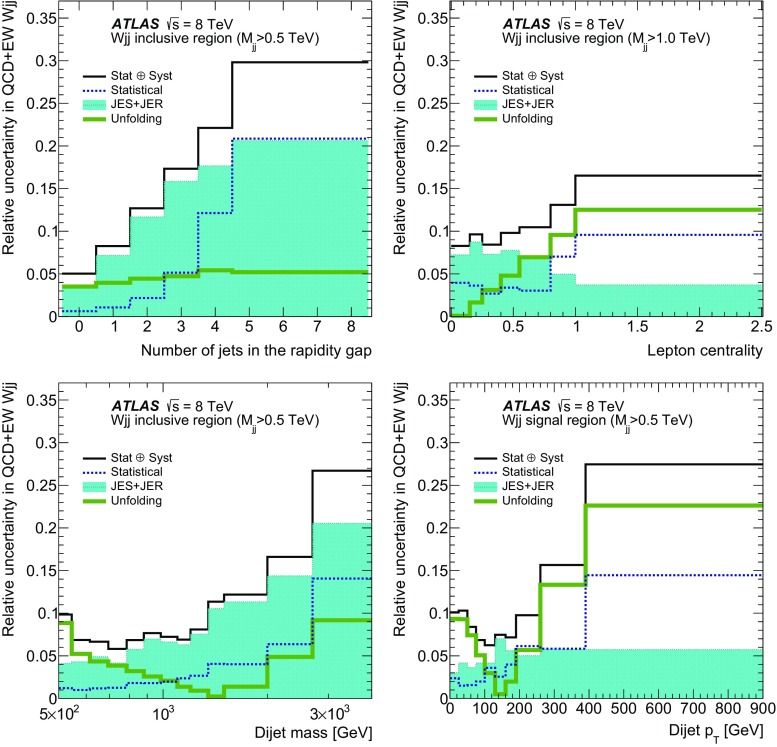
Relative uncertainties in example unfolded differential cross sections for the combined QCD+EW $$Wjj$$ processes. The examples are: the number of jets in the rapidity gap between the two highest-$$p_{\text {T}} $$ jets in the inclusive region (*top left*); the lepton centrality distribution in the inclusive $$M_{jj} >1$$ TeV region (*top right*); $$M_{jj} $$ in the inclusive region (*bottom left*); and the dijet $$p_{\text {T}} $$ in the signal region (*bottom right*). Dominant contributions to the total systematic uncertainty are highlighted separately

**Fig. 12:**
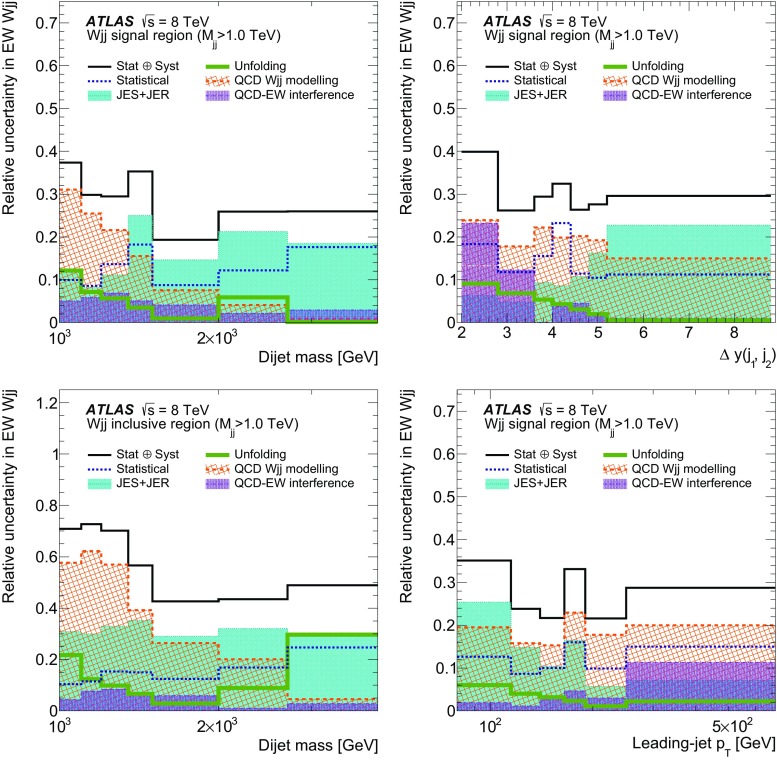
Relative uncertainties in example unfolded differential cross sections for the EW $$Wjj$$ processes. The examples are $$M_{jj} $$ (*top left*) and $$\Delta y(j_1,j_2) $$ (*top right*) in the high-mass signal region; $$M_{jj} $$ in the $$M_{jj} >1$$ TeV inclusive region (*bottom left*); and leading-jet $$p_{\text {T}} $$ in the high-mass signal region (*bottom right*). Dominant contributions to the total systematic uncertainty are highlighted separately

Figures 11 and 12 summarize the uncertainty contributions to example unfolded data distributions for QCD+EW $Wjj$ and EW $Wjj$ production, respectively. For measurements of combined QCD+EW $Wjj$ production, the jet energy scale and resolution uncertainties dominate the total uncertainty except in regions where statistical uncertainties are significant. The unfolding uncertainty is typically relevant in these regions and in regions dominated by QCD $Wjj$ production where the statistical uncertainties are small. In measurements of EW $Wjj$ production, uncertainties in the modelling of strong $Wjj$ production are particularly important at low dijet invariant mass, where the EW $Wjj$ signal purity is lowest. Interference uncertainties become dominant at low dijet rapidity separation but are otherwise not the leading contribution to the total uncertainty. A recent study [81] of interference in *Z*+jets VBF topologies, incorporating NLO electroweak corrections, predicted similar behaviour. For the bulk of the EW $Wjj$ distributions, the leading sources of uncertainty are statistical, QCD $Wjj$ modelling, and jet energy scale and resolution, and contribute roughly equally.

### Fiducial regions and integrated cross sections

The differential cross sections of the combined $Wjj$ processes are measured in the following nine fiducial regions:

- the four mutually orthogonal fiducial regions defined in Fig. 4, three of which are electroweak-suppressed (${<}$5% contribution) and one electroweak-enhanced (15–20% contribution);
- an additional electroweak-enhanced signal region with $M_{jj} >1.0$ $\text {TeV}$ (35–40% electroweak $Wjj$ contribution); and
- four inclusive fiducial regions defined by the preselection requirements in Table 1 with $M_{jj} > 0.5,~1.0,~1.5$ and 2.0 $\text {TeV}$.

The inclusive fiducial regions probe the observables used to distinguish EW and QCD $Wjj$ production, namely lepton and jet centrality, and the number of jets radiated in the rapidity gap between the two leading jets. The four successively higher invariant mass thresholds increasingly enhance the EW $Wjj$ purity of the differential distributions, without lepton and jet topology requirements.

The combined QCD+EW $Wjj$ production is measured in all regions to test the modelling of QCD $Wjj$ production in a VBF topology. In regions sensitive to EW $Wjj$ contributions, the prediction for QCD $Wjj$ only is shown along with the combined QCD+EW $Wjj$ prediction in order to indicate the effect of the EW $Wjj$ process. Differential measurements of EW $Wjj$ production are performed in regions with $M_{jj} >1.0$ $\text {TeV}$, where the expected EW $Wjj$ fraction is ${>}20\%$. The QCD $Wjj$ background is subtracted using the multiplicative normalization factor of $\mu _{\text {QCD}} = 1.09 \pm 0.02$ (stat) determined from the fits in Sect. 5. This substantially reduces the normalization uncertainty, confining theoretical uncertainties to the shapes of the background distributions.

Performing a complete unfolding of the EW $Wjj$ signal process leads to better precision on the unfolded data, particularly in the case of normalized distributions, than could be achieved by subtracting the particle-level QCD $Wjj$ production background from unfolded QCD+EW $Wjj$ production data. All EW $Wjj$ differential measurements are nonetheless also performed as combined QCD+EW $Wjj$ production measurements so that such a subtraction could be performed with other QCD $Wjj$ predictions.

**Fig. 13:**
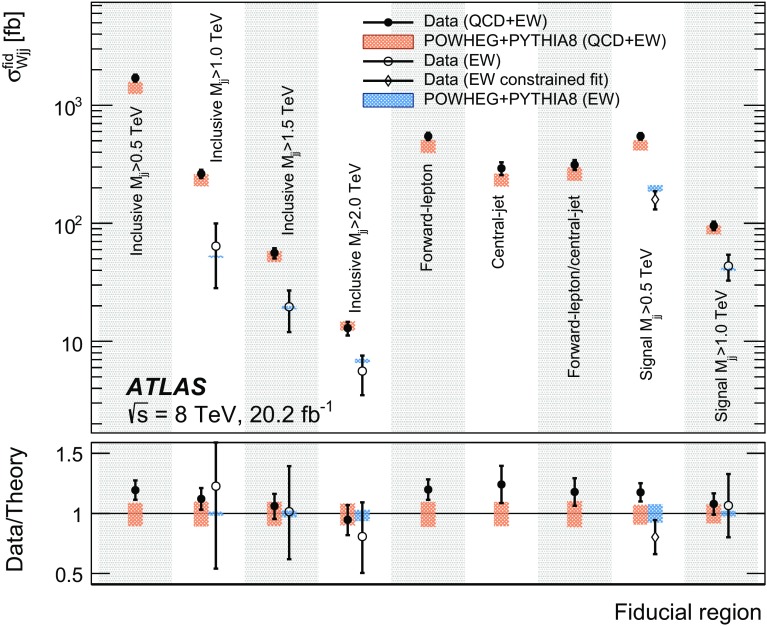
Integrated production cross sections for QCD+EW $Wjj$ (*solid* data points) and EW $Wjj$ (*open* data points) production in each measured particle-level fiducial region in a single lepton channel; EW $Wjj$ production is only measured in fiducial regions where there is sufficient purity. For each measurement the *error bar* represents the statistical and systematic uncertainties summed in quadrature. Comparisons are made to predictions from Powheg + Pythia8 and the *bottom pane* shows the ratio of data to these predictions

Integrated cross sections for $Wjj$ production are determined in each fiducial region. Table 7 and Fig. 13 show the measured integrated production cross sections for a single lepton flavour ($\sigma ^\mathrm {fid}_{W}$) for QCD+EW $Wjj$ production and, in high dijet invariant-mass regions, for EW $Wjj$ production. Also shown is the value of the EW $Wjj$ cross section extracted from the constrained fit described in Sect. 5.3. All measurements are broadly compatible with predictions from Powheg + Pythia8. In fiducial regions dominated by QCD $Wjj$ production the measured cross sections are approximately 15–20% higher than predictions. The integrated EW $Wjj$ production cross sections have larger relative uncertainties than the precisely constrained fiducial EW $Wjj$ cross-section measurement.

**Table 7:** Integrated fiducial cross sections for QCD+EW and EW $Wjj$ production and the equivalent predictions from Powheg + Pythia8. The uncertainties displayed are the values of the statistical and systematic uncertainties added in quadrature

Fiducial region	$\sigma ^\mathrm {fid}_{W}$ [fb]			
	QCD+EW		EW	
	Data	Powheg + Pythia8	Data	Powheg + Pythia8
Inclusive $M_{jj} >0.5$ $\text {TeV}$	$1700\pm 110$	$1420\pm 150$	–	–
Inclusive $M_{jj} >1.0$ $\text {TeV}$	$263\pm 21$	$234\pm 26$	$64\pm 36$	$52\pm 1$
Inclusive $M_{jj} >1.5$ $\text {TeV}$	$56\pm 5$	$53\pm 5$	$20\pm 8$	$19\pm 0.5$
Inclusive $M_{jj} >2.0$ $\text {TeV}$	$13\pm 2$	$14\pm 1$	$5.6\pm 2.1$	$6.9\pm 0.2$
Forward-lepton	$545\pm 39$	$455\pm 51$	–	–
Central-jet	$292\pm 36$	$235\pm 28$	–	–
Forward-lepton/central-jet	$313\pm 30$	$265\pm 32$	–	–
Signal $M_{jj} >0.5$ $\text {TeV}$	$546\pm 35$	$465\pm 39$	$159\pm 25$	$198\pm 12$
Signal $M_{jj} >1.0$ $\text {TeV}$	$96\pm 8$	$89\pm 7$	$43\pm 11$	$41\pm 1$

**Fig. 14:**
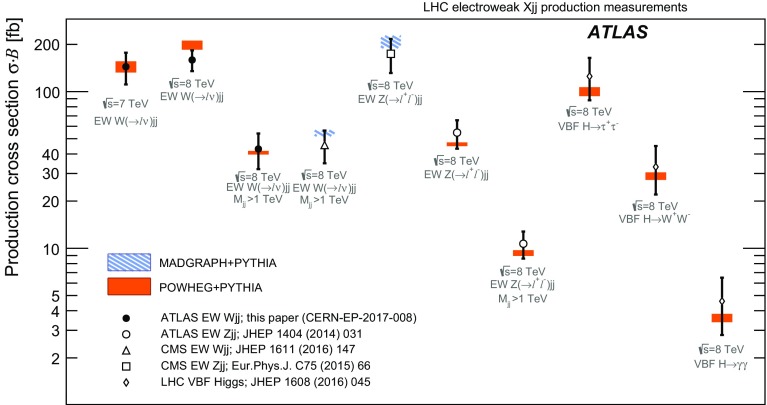
Measurements of the cross sections times branching fractions of electroweak production of a single *W*, *Z*, or Higgs boson with two jets at high dijet invariant mass and in fiducial measurement regions. For each measurement the *error bar* represents the statistical and systematic uncertainties summed in quadrature. *Shaded bands* represent the theory predictions. The $M_{jj}$ threshold defining the fiducial *Zjj* region differs between ATLAS and CMS, leading to different inclusive cross sections

The measurements of electroweak $$Wjj $$ fiducial cross sections are compared to measurements of electroweak *Zjj* production and VBF Higgs boson production in Fig. 14. These other measurements are extrapolated to lower dijet mass (for *Zjj* production) or to inclusive production (for Higgs boson production) so their apparent cross sections are generally increased relative to the $$Wjj$$ fiducial cross sections.

### Observables distinguishing QCD $Wjj$ and EW $Wjj$

Differential measurements are performed in the following distributions that provide discrimination between strong and electroweak $Wjj$ production:

- $$M_{jj} $$, the invariant mass of the two highest-$$p_{\text {T}} $$ jets;
- $\Delta y(j_1,j_2)$, the absolute rapidity separation between the two highest-$p_{\text {T}}$ jets;
- $C_\ell$, lepton centrality, the location in rapidity of the lepton relative to the average rapidity of the two highest-$p_{\text {T}}$ jets, defined in Eq. (1);
- $C_j$, jet centrality, the location in rapidity of any additional jet relative to the average rapidity of the two highest-$p_{\text {T}}$ jets, defined in Eq. (1); and
- $N_{\text {jets}}^{\text {gap}}$, the number of additional jets in the rapidity gap bounded by the two highest-$p_{\text {T}}$ jets (i.e., jets with $C_j < 0.5$).

**Fig. 15:**
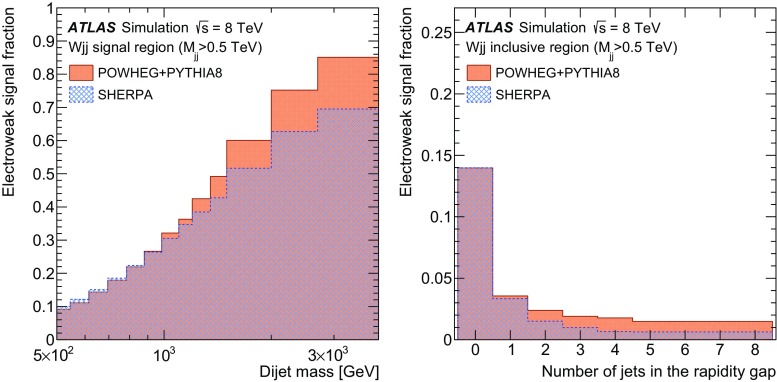
Fraction of EW $Wjj$ signal relative to the combined QCD+EW $Wjj$ production, predicted by Powheg + Pythia8 and Sherpa simulations for observables in the signal (*left*) and inclusive (*right*) fiducial regions

The first two observables use the dijet system to distinguish the *t*-channel VBF topology from the background. The remaining observables use the rapidity of other objects relative to the dijet rapidity gap, exploiting the colourless gauge boson exchange to distinguish the EW $Wjj$ signal from the QCD $Wjj$ background. Figure 15 shows the Powheg + Pythia8 and Sherpa predictions of the fraction of $Wjj$ events produced via electroweak processes, as a function of the dijet invariant mass in the signal fiducial region and the number of jets emitted in the dijet rapidity gap for the inclusive fiducial region with $M_{jj} >0.5$ $\text {TeV}$.

#### Dijet observables

**Fig. 16:**
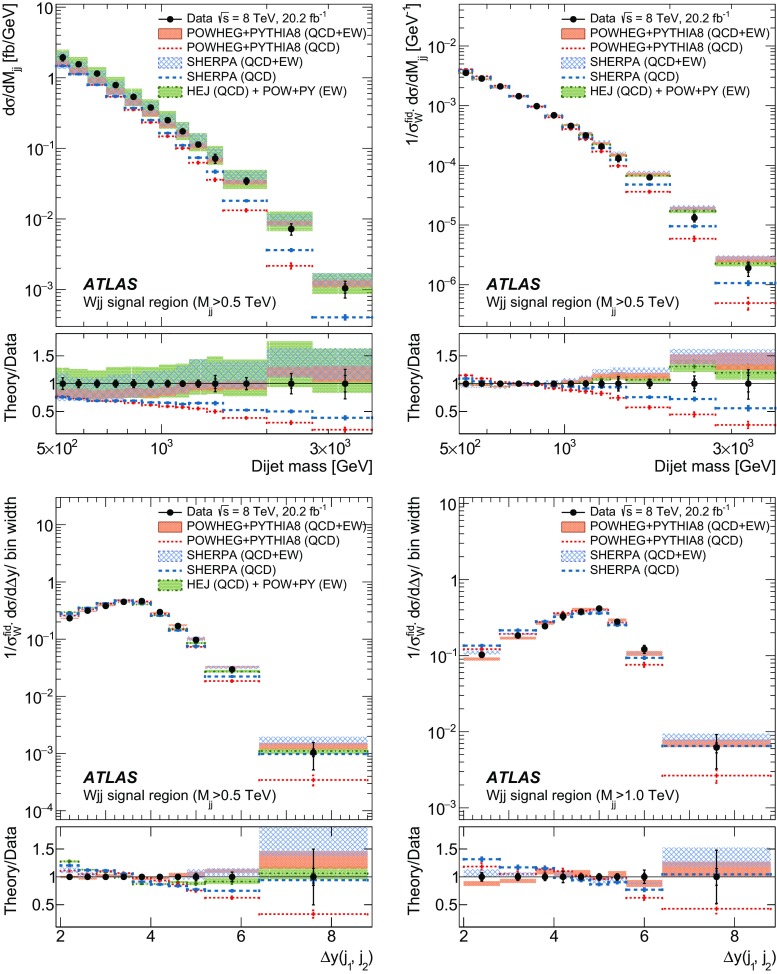
*Top* Unfolded absolute (*left*) and normalized (*right*) differential $Wjj$ production cross sections as a function of dijet mass for the signal fiducial region. *Bottom* Unfolded normalized production cross sections as a function of $\Delta y(j_1,j_2)$ for the signal regions with $M_{jj} > 0.5$ $\text {TeV}$ (*left*) and $M_{jj} > 1.0$ $\text {TeV}$ (*right*). Both statistical (*inner bar*) and total (*outer bar*) measurement uncertainties are shown, as well as ratios of the theoretical predictions to the data (the *bottom panel* in each distribution)

The best discrimination between QCD and EW $Wjj$ production is provided by the dijet mass distribution, as demonstrated in the top plots of Fig. 16. The distribution of dijet rapidity separation is correlated with this distribution but is purely topological. The discrimination provided by $\Delta y(j_1,j_2)$ is shown in the bottom plots of the figure for $M_{jj} >0.5$ and 1 $\text {TeV}$.

**Fig. 17:**
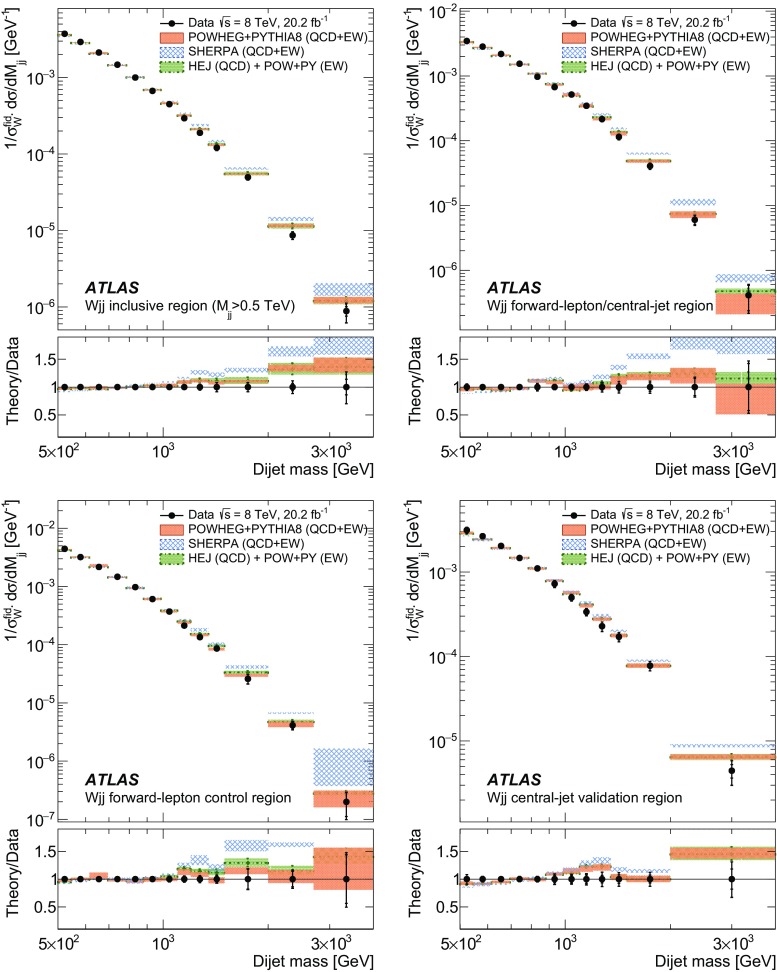
Unfolded normalized differential $Wjj$ production cross sections as a function of dijet invariant mass in the inclusive, forward-lepton/central-jet, forward-lepton, and central-jet fiducial regions. Both statistical (*inner bar*) and total (*outer bar*) measurement uncertainties are shown, as well as ratios of the theoretical predictions to the data (the *bottom panel* in each distribution)

**Fig. 18:**
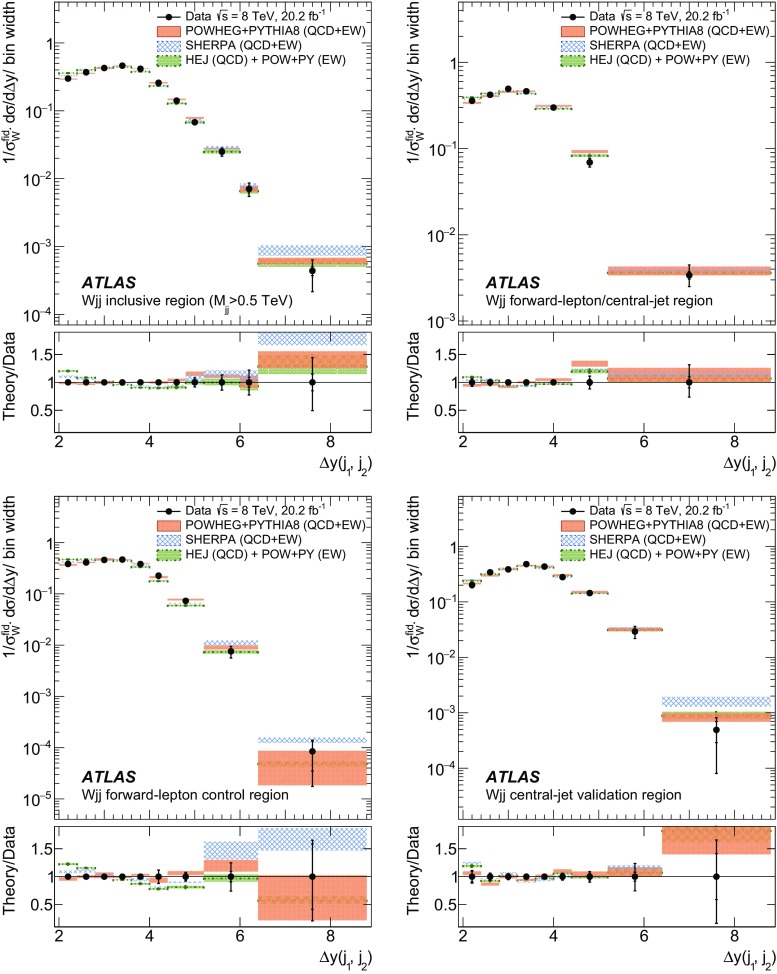
Unfolded normalized differential $Wjj$ production cross sections as a function of $\Delta y(j_1,j_2)$ in the inclusive, forward-lepton/central-jet, forward-lepton, and central-jet fiducial regions. Both statistical (*inner bar*) and total (*outer bar*) measurement uncertainties are shown, as well as ratios of the theoretical predictions to the data (the *bottom panel* in each distribution)

The QCD $Wjj$ modelling of the dijet distributions is important for extracting the cross section for EW $Wjj$ production. The modelling of the $M_{jj}$ distribution in regions dominated by QCD $Wjj$ production is shown in Fig. 17. Predictions from hej, which are expected to provide a good description at high dijet invariant mass where large logarithms contribute, are similar to the NLO predictions from Powheg + Pythia8. Sherpa predicts more events at high dijet invariant mass than observed in data in these fiducial regions, whereas Powheg + Pythia8 and hej are in better agreement with data. The dijet rapidity separation (Fig. 18) shows similar behavior, with Sherpa overestimating the rate at large separation. The hej distributions have larger deviations from the data due to the reduced accuracy of resummation at small $\Delta y(j_1,j_2)$.

**Fig. 19:**
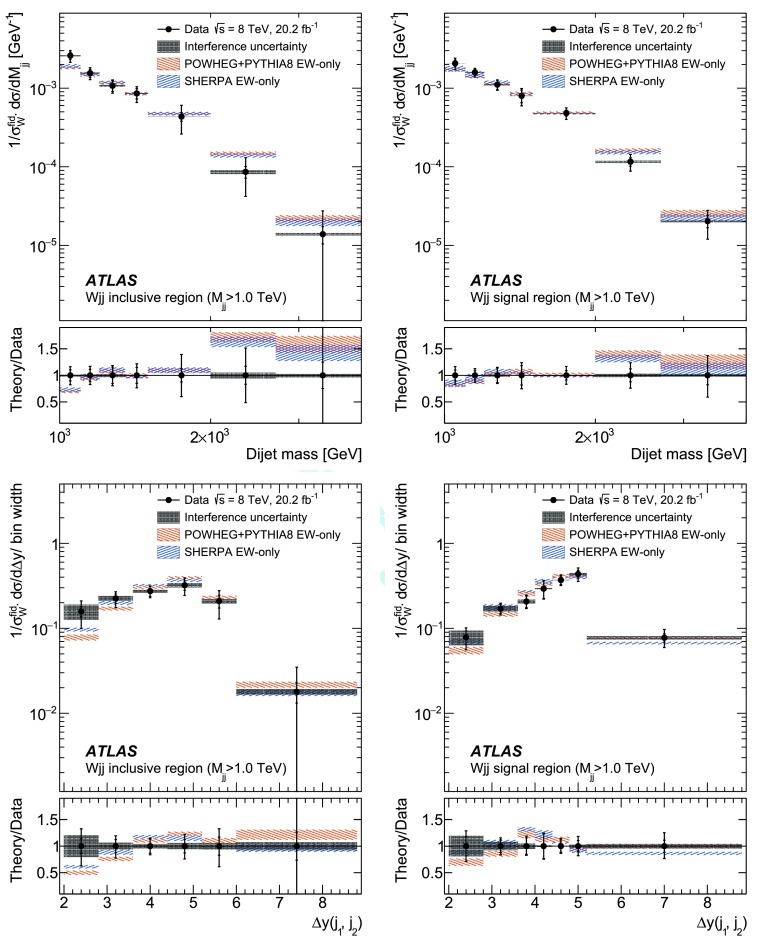
Unfolded normalized differential EW $Wjj$ production cross sections as a function of the dijet invariant mass (*top*) and $\Delta y(j_1,j_2)$ (*bottom*) for the inclusive (*left*) and signal (*right*) fiducial regions with $M_{jj} > 1.0$ $\text {TeV}$. Both statistical (*inner bar*) and total (*outer bar*) measurement uncertainties are shown, as well as ratios of the theoretical predictions to the data (the *bottom panel* in each distribution)

The dijet distributions are generally well modelled for the EW $Wjj$ process, as shown in Fig. 19 for the inclusive and signal regions with $M_{jj} >1.0$ $\text {TeV}$. The reduced purity in the inclusive region causes larger measurement uncertainties, and the measurements have larger absolute discrepancies with respect to predictions. The interference uncertainty is largest at low $\Delta y(j_1,j_2)$, where the topology is less VBF-like.

#### Object topology relative to the rapidity gap

The event topology distinguishes electroweak VBF production from other processes, in particular the lack of hadronic activity in the rapidity gap between the leading two jets and the tendency for the boson to be emitted within this gap. These topological features are studied using the distributions of the jet multiplicity in the gap, the fraction of events with no jets with the gap, and the rapidity of the lepton and jets relative to the gap.

**Fig. 20:**
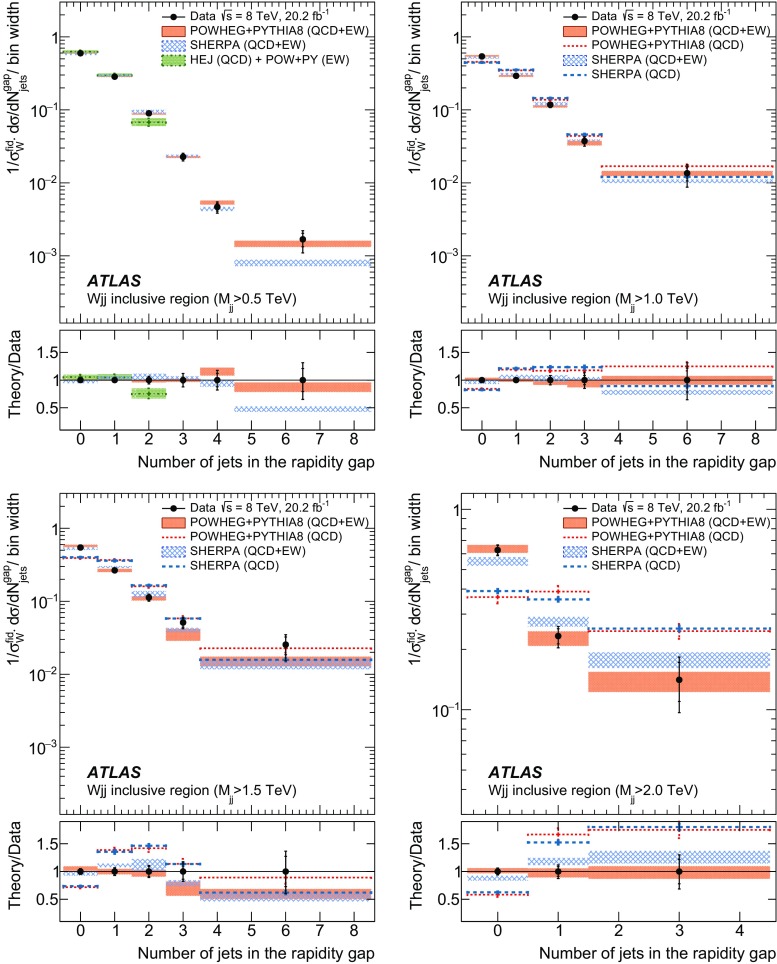
Unfolded normalized distribution of the number of jets with $$p_{\text {T}} >30$$ $$\text {GeV}$$ in the rapidity interval bounded by the two highest-$$p_{\text {T}} $$ jets in the inclusive fiducial region with $$M_{jj} $$ thresholds of 0.5 $$\text {TeV}$$ (*top left*), 1.0 $$\text {TeV}$$ (*top right*), 1.5 $$\text {TeV}$$ (*bottom left*), and 2.0 $$\text {TeV}$$ (*bottom right*). Both statistical (*inner bar*) and total (*outer bar*) measurement uncertainties are shown, as well as ratios of the theoretical predictions to the data (the *bottom panel* in each distribution)

**Fig. 21:**
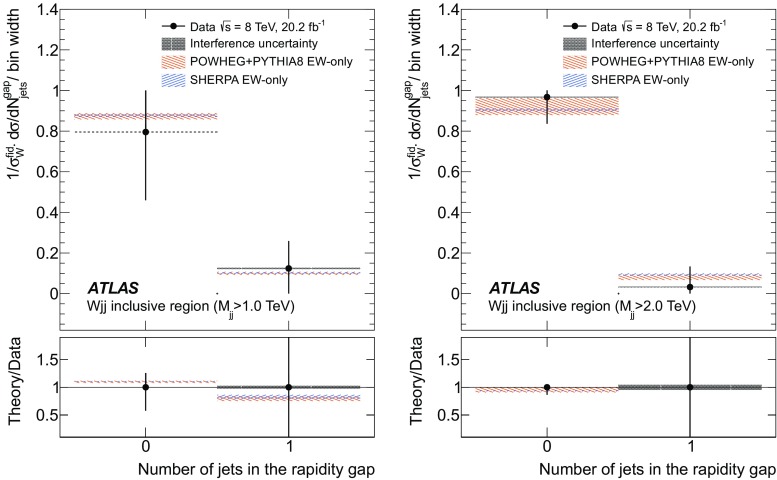
Unfolded normalized differential EW $Wjj$ production cross sections as a function of the number of jets with $p_{\text {T}} >30$ $\text {GeV}$ in the rapidity interval bounded by the two highest-$p_{\text {T}}$ jets in the inclusive fiducial region, with $M_{jj} > 1.0$ $\text {TeV}$ (*left*) and $M_{jj} > 2.0$ $\text {TeV}$ (*right*). Both statistical (*inner bar*) and total (*outer bar*) measurement uncertainties are shown, as well as ratios of the theoretical predictions to the data (the *bottom panel* in each distribution)

Figure 20 shows the normalized differential cross section as a function of the number of $$p_{\text {T}} >30$$ $$\text {GeV}$$ jets emitted into the rapidity gap for progressively increasing $$M_{jj}$$ thresholds. In the lowest invariant-mass fiducial region, strong $$Wjj$$ production dominates and predictions from Powheg + Pythia8, Sherpa, and hej all describe the data well. As the dijet invariant mass threshold is increased, the differences in shape between predictions with and without the EW $$Wjj$$ contribution become apparent. The corresponding differential measurements for EW $$Wjj$$ production are shown in Fig. 21 for the inclusive regions with $$M_{jj} > 1.0$$ and 2.0 $$\text {TeV}$$. The measured fraction of EW $$Wjj$$ events with no additional central jets is higher than that of QCD+EW $$Wjj$$ events, as also demonstrated in Table 8. The table shows that the measured zero-jet fraction, frequently referred to as the jet-veto efficiency, is consistent with the Powheg + Pythia8 QCD+EW $$Wjj$$ prediction for progressively increasing $$M_{jj} $$. As $$M_{jj} $$ increases the relative contribution of the EW $$Wjj$$ process increases substantially.

**Table 8:** Jet-veto efficiency for each $$M_{jj} $$ threshold compared to Powheg + Pythia8 QCD+EW and QCD $$Wjj$$ simulations. The uncertainties comprise statistical and systematic components added in quadrature

	Jet-veto efficiency			
	$M_{jj} >0.5$ TeV	$M_{jj} >1.0$ TeV	$M_{jj} >1.5$ TeV	$M_{jj} >2.0$ TeV
Data	$0.596\pm 0.014$	$0.54\pm 0.02$	$0.55\pm 0.03$	$0.63\pm 0.04$
Powheg +Pythia8 (QCD+EW)	$0.597\pm 0.005$	$0.55\pm 0.01$	$0.57\pm 0.02$	$0.63\pm 0.03$
Powheg +Pythia8 (QCD)	$0.569\pm 0.002$	$0.45\pm 0.01$	$0.39\pm 0.01$	$0.36\pm 0.03$

**Fig. 22:**
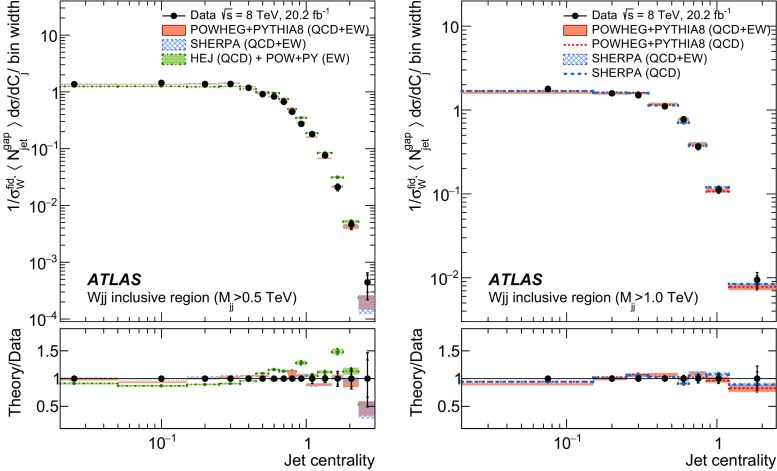
Unfolded normalized differential QCD+EW $Wjj$ production cross sections as a function of jet centrality for the inclusive fiducial region with $M_{jj} > 0.5$ $\text {TeV}$ (*left*) and 1.0 $\text {TeV}$ (*right*). Both statistical (*inner bar*) and total (*outer bar*) measurement uncertainties are shown, as well as ratios of the theoretical predictions to the data (the *bottom panel* in each distribution)

Jet centrality is related to the number of jets in the rapidity gap, as events with $C_j < 0.5$ have a jet within the gap. Figure 22 shows good agreement between the predictions and data in the QCD+EW $Wjj$ differential cross section weighted by the mean number of gap jets. Since the rate for additional jet production is low in EW $Wjj$ production, there are too few events to perform a measurement of the jet centrality distribution for this process.

**Fig. 23:**
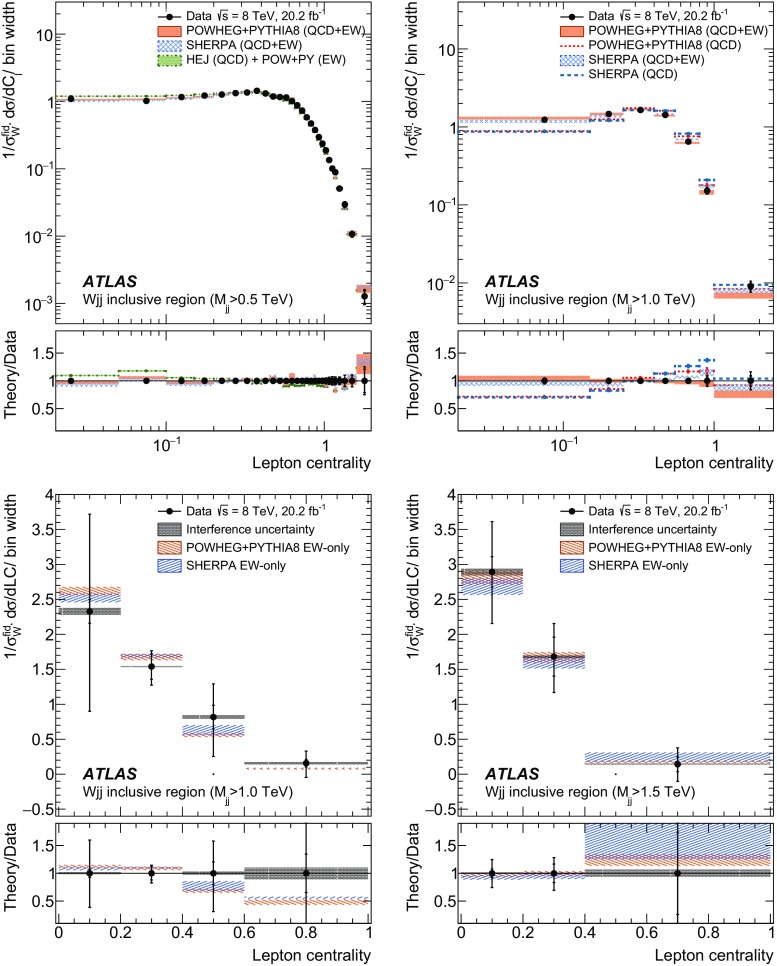
Unfolded normalized differential QCD+EW $Wjj$ (*top*) and EW (*bottom*) production cross sections as a function of lepton centrality for the inclusive fiducial region with $M_{jj} > 0.5$ $\text {TeV}$ (*top left*), 1.0 $\text {TeV}$ (*top right* and *bottom left*), and 1.5 $\text {TeV}$ (*bottom right*). Both statistical (*inner bar*) and total (*outer bar*) measurement uncertainties are shown, as well as ratios of the theoretical predictions to the data (the *bottom panel* in each distribution)

The lepton centrality distribution indirectly probes the rapidity of the *W* boson relative to the dijet rapidity interval. The differential cross section in the inclusive region as a function of lepton centrality is shown in Fig. 23 for three $$M_{jj} $$ thresholds. All QCD+EW $$Wjj$$ predictions adequately describe the lepton centrality in the region with the lowest dijet mass threshold, which is dominated by QCD $$Wjj$$ production. As the $$M_{jj} $$ threshold is increased the differences between QCD and QCD+EW $$Wjj$$ production become more apparent, particularly at low lepton centrality where EW $$Wjj$$ production is enhanced. The measurement of this distribution for EW $$Wjj$$ production shows good agreement with the predictions.

### Observables sensitive to anomalous gauge couplings

Differential measurements are performed in distributions that provide enhanced sensitivity to anomalous gauge couplings:

- $p_{\text {T}} ^{j_1}$, the $p_{\text {T}}$ of the highest-$p_{\text {T}}$ jet;
- $p_{\text {T}} ^{jj}$, the $p_{\text {T}}$ of the dijet system (vector sum of the $p_{\text {T}}$ of the two highest-$p_{\text {T}}$ jets); and
- $\Delta \phi (j_1,j_2)$, the magnitude of the azimuthal angle between the two highest-$p_{\text {T}}$ jets,

where the last observable is sensitive to anomalous CP-violating couplings [82].

**Fig. 24:**
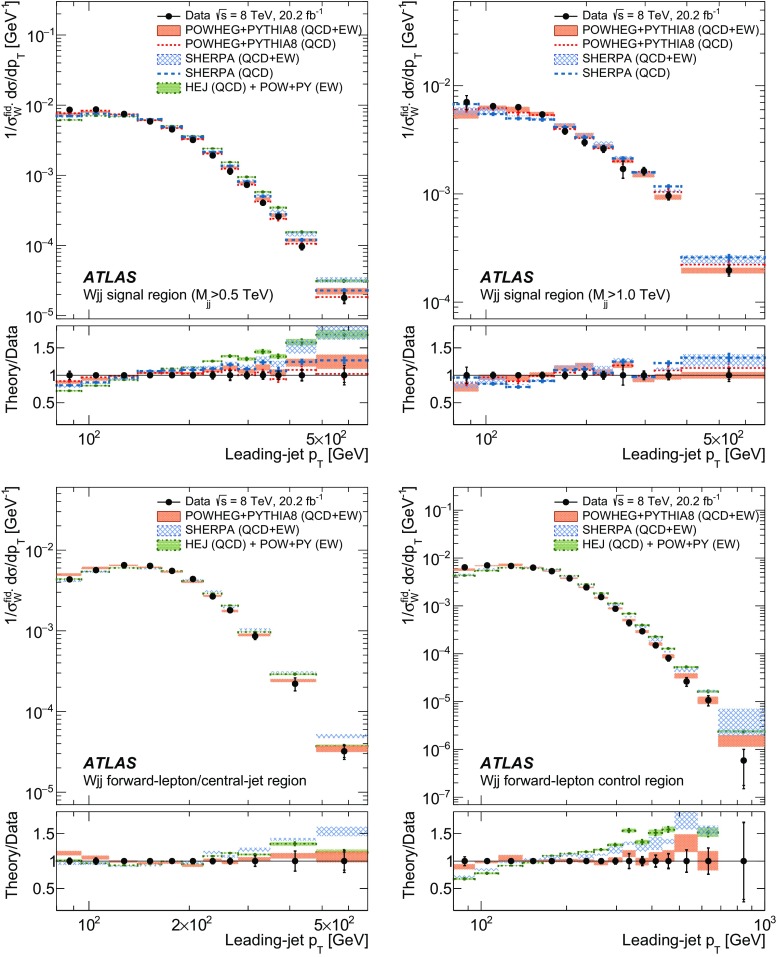
Unfolded normalized differential $Wjj$ production cross sections as a function of the leading-jet $p_{\text {T}}$ in the signal, high-mass signal, forward-lepton/central-jet, forward-lepton, and central-jet fiducial regions. Both statistical (*inner bar*) and total (*outer bar*) measurement uncertainties are shown, as well as ratios of the theoretical predictions to the data (the *bottom panel* in each distribution)

**Fig. 25:**
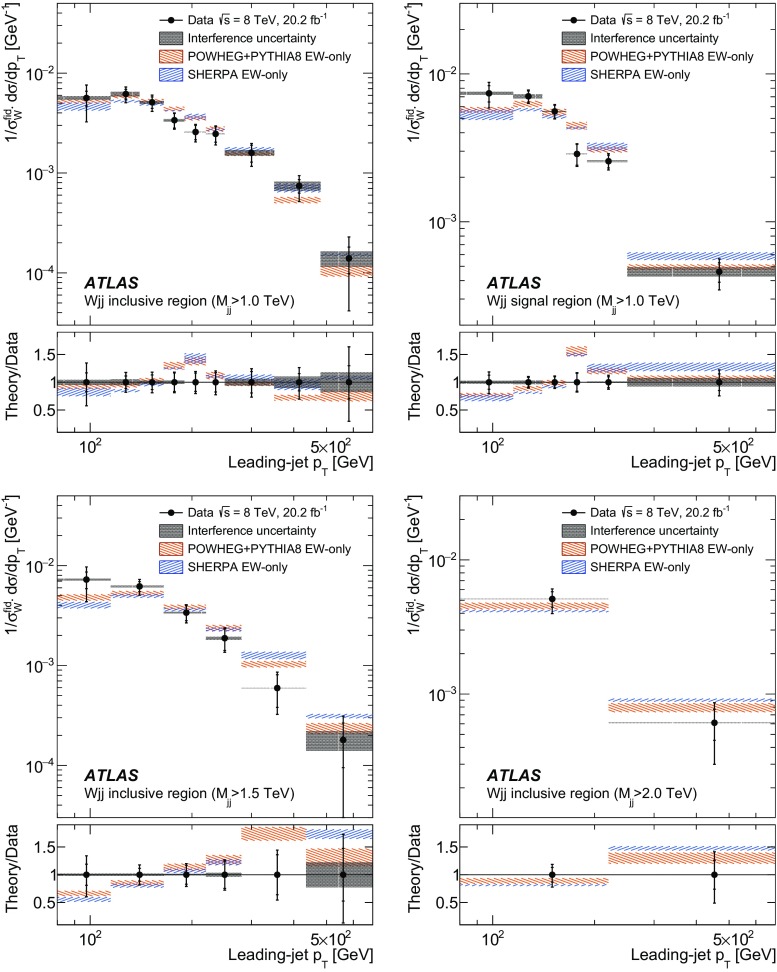
Unfolded normalized differential EW $Wjj$ production cross sections as a function of the leading-jet $p_{\text {T}}$ for the inclusive fiducial region with three thresholds on the dijet invariant mass (1.0, 1.5, and 2.0 $\text {TeV}$), and for the signal-enriched fiducial region with a minimum dijet invariant mass of 1.0 $\text {TeV}$. Both statistical (*inner bar*) and total (*outer bar*) measurement uncertainties are shown, as well as ratios of the theoretical predictions to the data (the *bottom panel* in each distribution)

The transverse momentum distribution of the leading jet, shown in Fig. 24, has a substantial correlation with the momentum transfer in *t*-channel events. The QCD+EW $Wjj$ measurements are globally well described by Powheg + Pythia8, while predictions from Sherpa and hej both show a harder spectrum than observed in data. For EW $Wjj$ production the Powheg + Pythia8 and Sherpa predictions give a harder spectrum than observed in the data, particularly in the higher purity regions (Fig. 25). The overestimation of rates at high jet $p_{\text {T}}$ may be reduced by the inclusion of NLO electroweak corrections [66].

**Fig. 26:**
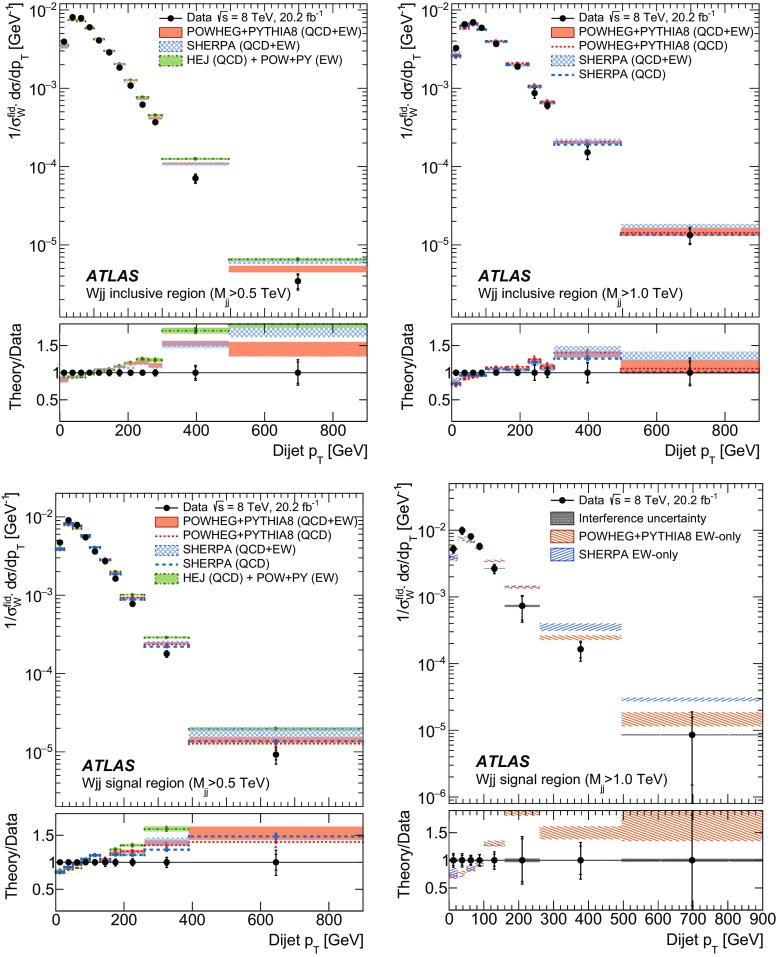
Unfolded normalized differential $Wjj$ production cross sections as a function of dijet $p_{\text {T}}$ for the inclusive (*top*) and signal (*bottom*) regions with $M_{jj} >0.5$ $\text {TeV}$ (*left*) and $M_{jj} > 1.0$ $\text {TeV}$ (*right*). The *bottom right* distribution shows EW $Wjj$ production and the other distributions show QCD+EW $Wjj$ production. Both statistical (*inner bar*) and total (*outer bar*) measurement uncertainties are shown, as well as ratios of the theoretical predictions to the data (the *bottom panel* in each distribution)

The transverse momentum of the dijet system is also correlated with the momentum transfer in *t*-channel events. Figure 26 shows the measured normalized $p_{\text {T}}$ distribution of the dijet system compared to the various predictions. There is a trend for all predictions to overestimate the relative rate at high dijet $p_{\text {T}}$ in the inclusive and signal-enhanced regions, both for QCD+EW $Wjj$ and EW $Wjj$ production. As in the case of the jet $p_{\text {T}}$ distribution, the discrepancy could be due to missing NLO electroweak corrections, which reduce the predictions at high *W*-boson $p_{\text {T}}$  [66].

**Fig. 27:**
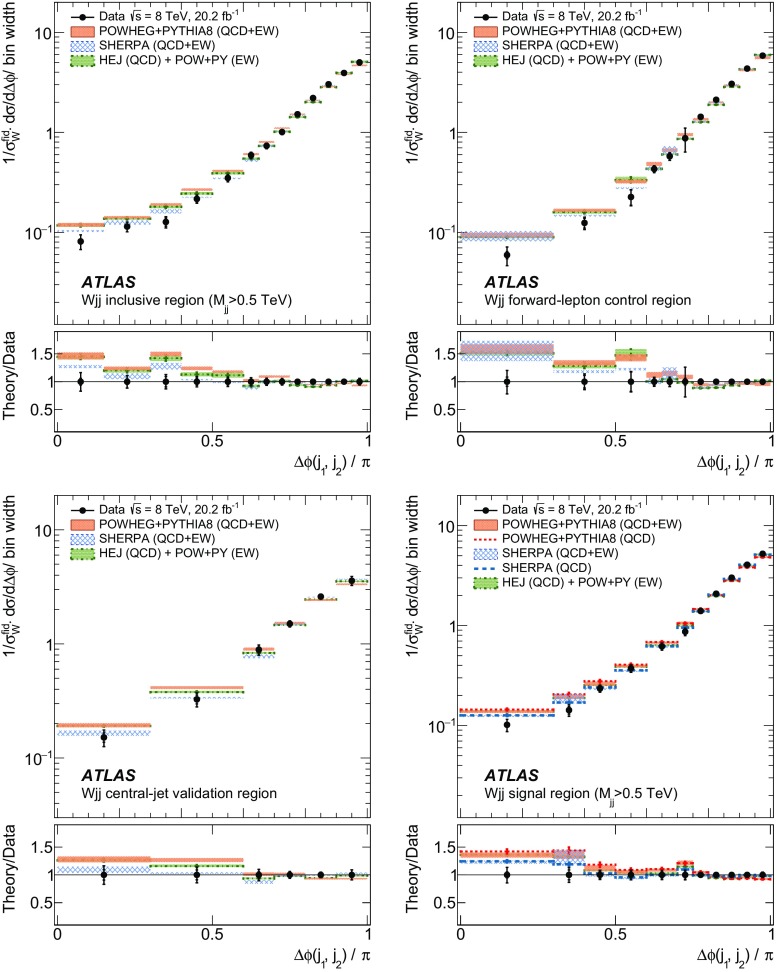
Unfolded normalized differential $Wjj$ production cross sections as a function of $\Delta \phi (j_1,j_2)$ for the inclusive, forward-lepton control, central-jet validation, and signal fiducial regions. Both statistical (*inner bar*) and total (*outer bar*) measurement uncertainties are shown, as well as ratios of the theoretical predictions to the data (the *bottom panel* in each distribution)

**Fig. 28:**
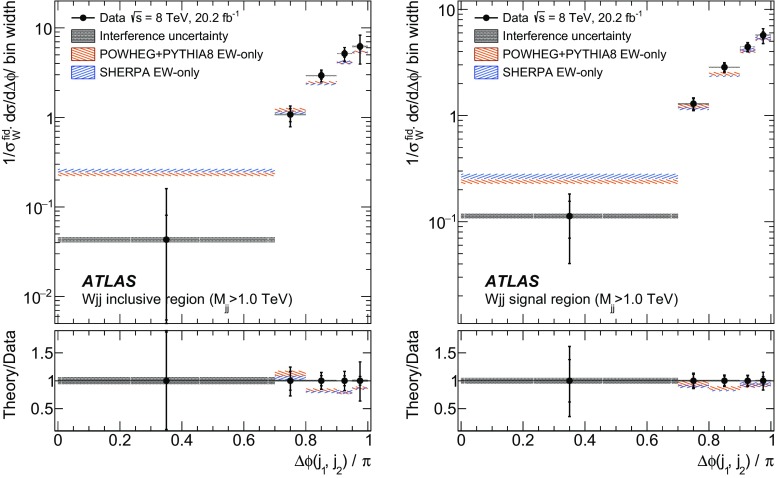
Unfolded normalized differential EW $Wjj$ production cross sections as a function of the azimuthal angle between the two leading jets, for the inclusive and signal fiducial regions with $M_{jj} > 1.0$ $\text {TeV}$. Both statistical (*inner bar*) and total (*outer bar*) measurement uncertainties are shown, as well as ratios of the theoretical predictions to the data (the *bottom panel* in each distribution)

The azimuthal angle between the two leading jets can be used to probe for new CP-odd operators in VBF production. The normalized differential cross sections for QCD+EW $Wjj$ production as a function of this angle are shown in the inclusive, forward-lepton control, central-jet validation, and signal fiducial regions in Fig. 27. Good agreement between the data and all predictions is seen, with a slight tendency for predictions to overestimate the relative rate at small angles in all fiducial regions. Figure 28 shows the normalized EW $Wjj$ cross section as a function of the azimuthal angle between the two leading jets for the inclusive and signal fiducial regions with $M_{jj} >1.0$ $\text {TeV}$.

## Anomalous triple-gauge-boson couplings

The triple-gauge-boson vertex is directly probed by the vector-boson-fusion process. Non-SM couplings at this vertex would affect the production rates and distributions. The couplings are constrained in the context of an aTGC or EFT framework, using the yield in the anomalous coupling signal region (Table 1) to constrain the parameters. The results are complementary [83] to those obtained in diboson production [84], which corresponds to the exchange of one off-shell boson in the *s*-channel rather than two in the *t*-channel.

### Theoretical overview

The signal-region measurements are sensitive to the *WWV* (*V* = *Z* or $\gamma$) couplings present in the *t*-channel production mode shown in Fig. 1a. These couplings can be characterized by an effective Lagrangian $\mathcal {L}^{WWV}_\mathrm {eff}$ including operators up to mass-dimension six [34]:

$$\begin{aligned} i\mathcal {L}^{WWV}_\mathrm {eff}&=g_{WWV}\, \biggl \{ \Bigl [g_1^V V^{\mu }(W^{-}_{\mu \nu }W^{+\nu } -W^{+}_{\mu \nu }W^{-\nu }) + \kappa _{V} W^{+}_{\mu }W^{-}_{\nu }V^{\mu \nu } + \frac{\lambda _{V}}{m_{W}^{2}}V^{\mu \nu }W^{+ \rho }_{\nu }W^{-}_{\rho \mu } \Bigr ]\\&\qquad - \Bigl [\frac{\tilde{\kappa }_{V}}{2} W^{-}_{\mu }W^{+}_{\nu } \epsilon ^{\mu \nu \rho \sigma } V_{\rho \sigma } + \frac{\tilde{\lambda }_{V}}{2 m_{W}^{2}} W^{-}_{\rho \mu }W^{+ \mu }_{\nu } \epsilon ^{ \nu \rho \alpha \beta } V_{\alpha \beta } \Bigr ]\biggr \}, \end{aligned}$$

where $W^{\pm }_{\mu \nu } = \partial _\mu W^{\pm }_\nu - \partial _\nu W^{\pm }_{\mu }$, with $W^{\pm }_\mu$ the $W^{\pm }$ field; $V_{\mu \nu } = \partial _\mu V_\nu - \partial _\nu V_\mu$, with $V_\mu$ the *Z* or $\gamma$ field; $m_W$ is the *W*-boson mass; and the individual couplings have SM values $g_1^V = 1$, $\kappa _{V} = 1$, $\lambda _{V} = 0$, $\tilde{\kappa }_{V} = 0$, and $\tilde{\lambda }_{V} = 0$. The overall coupling constants $g_{WWV}$ are given by $g_{WW\gamma } = -e$ and $g_{WWZ} = -e\cdot \cot (\theta _W)$, where *e* is the electromagnetic coupling and $\theta _W$ is the weak mixing angle. The terms in the first row of the Lagrangian conserve *C*, *P*, and *CP*, while those in the second violate *CP*. Deviations of the $g_1^V$ and $\kappa _{V}$ parameters from the SM are denoted by $\Delta g_1^Z= g_1^Z-1$ and $\Delta \kappa _{V}=\kappa _{V}-1$, respectively. The requirement of gauge invariance at the level of dimension-six operators leads to the following relations [85]:

$$\begin{aligned} \Delta g_1^Z = \Delta \kappa _Z + \Delta \kappa _{\gamma } \tan ^2\theta _W, \quad \lambda _{\gamma } = \lambda _Z \equiv \lambda _V, \quad g_1^{\gamma }=1, \quad \tilde{\kappa }_{\gamma } = - \tilde{\kappa }_Z \cot ^2\theta _W, \quad \mathrm { and } \quad \tilde{\lambda }_{\gamma } = \tilde{\lambda }_Z \equiv \tilde{\lambda }_V. \end{aligned}$$

The presence of anomalous couplings leads to unphysically large cross sections when the square of the momentum transfer $(q^2)$ between the incoming partons is large. To preserve unitarity, a form factor is introduced with a new-physics scale $\Lambda$ that suppresses the anomalous coupling at high energies:

$$\begin{aligned} \alpha (q^2) = \frac{\alpha }{(1+ q^2 / \Lambda ^2)^2}, \end{aligned}$$

where $\alpha$ is the anomalous coupling of interest. In the following, 95% confidence-level intervals are set for a unitarization scale of $\Lambda = 4 \, \text {TeV}$ and for a scale that effectively removes the form factor (shown as $\Lambda = \infty$). The scale $\Lambda = 4 \, \text {TeV}$ is chosen because it does not violate unitarity for any parameter in the expected range of sensitivity.

An alternative to the use of a form factor is to employ an effective field theory, which is an expansion in inverse powers of the energy scale of new interactions assuming perturbative coupling coefficients. An EFT allows the comprehensive investigation of a complete set of dimension-six operators in a Lagrangian with SM fields. The dimension-six terms introduced in the EFT can be expressed as

$$\begin{aligned} \mathcal {L}_{\text {EFT}} = \sum _i \frac{c_i}{\Lambda ^2}O_i, \end{aligned}$$

where $O_i$ are field operators with dimension 6, the scale of new physics is $\Lambda$, and $c_i$ are dimensionless coefficients. The operators relevant to triple-gauge-boson couplings in the HISZ basis [85] are

$$\begin{aligned} O_B= & {} (D_\mu H)^{\dagger } B^{\mu \nu }D_\nu H, \\ O_W= & {} (D_\mu H)^{\dagger } W^{\mu \nu }D_\nu H, \\ O_{WWW}= & {} \mathrm {Tr}[W_{\mu \nu } W^{\nu }_{\rho } W^{\rho \mu }], \\ O_{\tilde{W}}= & {} (D_\mu H)^{\dagger } \tilde{W}^{\mu \nu } D_\nu H, \\ O_{\tilde{W}WW}= & {} \mathrm {Tr}[W_{\mu \nu }W^{\nu }_{\rho } \tilde{W}^{\rho \mu }], \end{aligned}$$

where *H* is the Higgs-boson field, $B_{\mu \nu } = \partial _\mu B_\nu - \partial _\nu B_{\mu }$, $B^{\mu }$ is the U(1)$_\mathrm {Y}$ gauge field, and $\tilde{W}^{\mu \nu } = \frac{1}{2}\epsilon _{\mu \nu \rho \sigma }W^{\rho \sigma }$. The coefficients of these operators are related to the aTGC parameters via the following equations:

$$\begin{aligned} \frac{c_W}{\Lambda ^2}&= \frac{2}{m^2_Z} (g_1^Z -1), \\ \frac{c_B}{\Lambda ^2}&= \frac{2}{\tan ^2\theta _W m^2_Z}(g_1^Z -1)-\frac{2}{\sin ^2\theta _W m^2_Z} (\kappa _{Z} -1), \\ \frac{c_{WWW}}{\Lambda ^2}&= \frac{2}{3 g^2 m^2_W} \lambda _V, \\ \frac{c_{\tilde{W}}}{\Lambda ^2}&= -\frac{2}{\tan ^2\theta _W m^2_W} \tilde{\kappa }_Z, \\ \frac{c_{\tilde{W}WW}}{\Lambda ^2}&= \frac{2}{3 g^2 m^2_W} \tilde{\lambda }_V, \end{aligned}$$

where *g* is the weak coupling, $m_Z$ is the *Z*-boson mass, and the aTGC parameters do not have any form-factor suppression.

### Experimental method

The signal region defined to increase the sensitivity to anomalous triple-gauge-boson couplings requires $M_{jj} > 1$ $\text {TeV}$ and leading-jet $p_{\text {T}} > 600$ $\text {GeV}$ (Table 1). The leading-jet $p_{\text {T}}$ is chosen because it is highly correlated with the $q^2$ of the signal *t*-channel process. The $p_{\text {T}}$ threshold is optimized to maximize sensitivity to anomalous couplings, considering both the statistical and systematic uncertainties. The event yields in the reconstructed signal region used for setting the constraints are given in Table 4. The SM prediction is negligible for $p_{\text {T}} > 1$ $\text {TeV}$, yielding an approximate lower bound for the validity of the EFT constraints.

The effects of anomalous couplings are modelled with Sherpa. Each sample is normalized by a factor $k = \text {NLO}/\text {LO}$ given by the ratio of Powheg + Pythia8 to Sherpa SM predictions of electroweak $Wjj$ production. The number of events expected for a given parameter value is calculated as:

$$\begin{aligned} N_{\text {reco}}=\mathcal {L}\times \sigma ^{\ell \nu jj} \times \mathcal{{A}} \times \mathcal{{C}} \times k, \end{aligned}$$

where $\mathcal {L}$ is the integrated luminosity of the 8 $\text {TeV}$ data, $\sigma ^{\ell \nu jj}$ is the cross section for the corresponding anomalous-coupling variation, $\mathcal{{A}}$ is the selection acceptance at particle level, and $\mathcal{C}$ is the ratio of selected reconstruction-level events to the particle-level events in the fiducial phase-space region. The factor containing the cross section and acceptance ($\sigma ^{\ell \nu jj} \times \mathcal{{A}}$) is parameterized as a quadratic function of each aTGC parameter, with a 10% statistical uncertainty in the parameterization.

Theoretical uncertainties due to missing higher orders, estimated with factors of 2 and 1/2 variations of the renormalization and factorization scales, are estimated to be 8% of the strong $Wjj$ yield and 14% of the electroweak $Wjj$ yield in the region with leading-jet $p_{\text {T}} >600$ $\text {GeV}$. Detector uncertainties are correlated between strong and electroweak production and are estimated to be 11% of the combined yield.

### Confidence-level intervals for aTGC parameters

Confidence-level (C.L.) intervals are calculated using a frequentist approach [86]. A negative log-likelihood function is constructed based on the expected numbers of background and signal events, and the number of observed data events. The likelihood is calculated as a function of individual aTGC parameter variations, with the other parameters set to their SM values. To obtain 95% confidence-level intervals, pseudoexperiments are produced with the number of pseudodata events drawn from a Poisson distribution, where the mean is given by the total SM prediction Gaussian-fluctuated according to theoretical and experimental uncertainties.

**Table 9:** Expected and observed 95% C.L. allowed ranges for all aTGC parameters considered with the other parameters set to their SM values. A form factor with unitarization scale equal to 4 TeV enforces unitarity for all aTGC parameters. The results are derived from the high-$q^2$ region yields given in Table 4

	$\Lambda$ = 4 $\text {TeV}$		$\Lambda$ = $\infty$	
	Expected	Observed	Expected	Observed
$\Delta g_1^{Z}$	$[-0.39, 0.35]$	$[-0.32, 0.28]$	$[-0.16, 0.15]$	$[-0.13, 0.12]$
$\Delta \kappa _{Z}$	$[-0.38, 0.51]$	$[-0.29, 0.42]$	$[-0.19, 0.19]$	$[-0.15, 0.16]$
$\lambda _{V}$	$[-0.16, 0.12]$	$[-0.13, 0.090]$	$[-0.064, 0.054]$	$[-0.053, 0.042]$
$\tilde{\kappa }_{Z}$	$[-1.7, 1.8]$	$[-1.4, 1.4]$	$[-0.70, 0.70]$	$[-0.56, 0.56]$
$\tilde{\lambda }_{V}$	$[-0.13, 0.15]$	$[-0.10, 0.12]$	$[-0.058, 0.057]$	$[-0.047, 0.046]$

**Table 10:** Expected and observed 95% C.L. intervals for individual EFT coefficients divided by the square of the new physics scale $\Lambda$, with other coefficients set to zero. Intervals are calculated using the high-$q^2$ region yields (Table 4)

Parameter	Expected ($\text {TeV}$ $^{-2}$)	Observed ($\text {TeV}$ $^{-2}$)
$\frac{c_W}{\Lambda ^2}$	$[-39, 37]$	$[-33, 30]$
$\frac{c_B}{\Lambda ^2}$	$[-200, 190]$	$[-170, 160]$
$\frac{c_{WWW}}{\Lambda ^2}$	$[-16, 13]$	$[-13, 9]$
$\frac{c_{\tilde{W}}}{\Lambda ^2}$	$[-720, 720]$	$[-580, 580]$
$\frac{c_{\tilde{W}WW}}{\Lambda ^2}$	$[-14, 14]$	$[-11, 11]$

**Fig. 29:**
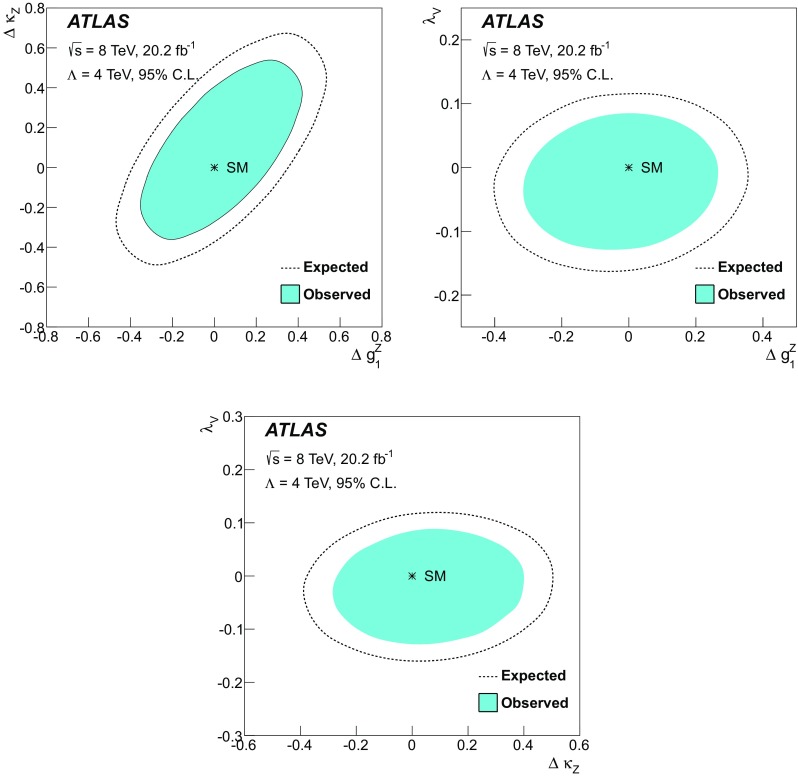
The observed (*solid blue*) and expected (*open dashed*) 95% C.L. allowed regions in two-parameter planes for $\Lambda = 4$ $\text {TeV}$. The regions are derived using a single measured yield and therefore reduce to the corresponding one-parameter interval when the other parameter is set to zero. Constraints on $\tilde{\lambda }_V$ are similar to those on $\lambda _V$

Tables 9 and 10 give the expected and observed 95% C.L. interval for each parameter probed, with the other parameters set to their SM values. All observed intervals are narrower than the expected intervals due to a slight deficit of data events compared with the SM prediction (Table 4). The $\lambda _V$ intervals are competitive with those derived from *WW* production [84]. The 95% C.L. regions in planes with two parameters deviating from their SM values are shown in Fig. 29. Since the regions are determined using a single measured yield, only the size of the region is constrained and not its shape. Thus, along an axis where one parameter is equal to zero, the corresponding one-parameter C.L. interval is recovered. The constraints on $\tilde{\lambda }_V$ are similar to $\lambda _V$ since the sensitivity is dominated by the square of the anomalous-coupling amplitude rather than its interference with the SM amplitude.

## Summary

Measurements of the fiducial and differential cross sections of electroweak production of *W* bosons in association with two jets have been performed using the lepton decay channel and events with high dijet invariant mass. The measurements use data collected by the ATLAS detector from proton–proton collisions at the LHC at centre-of-mass energies of $\sqrt{s}=7$ and 8 $\text {TeV}$, corresponding to 4.7 and 20.2 fb$^{-1}$ of integrated luminosity, respectively. The cross sections in a fiducial region with a signal purity of $\mathcal{{O}}$(15%) are

$$\begin{aligned} \sigma ^\mathrm {fid}_{\mathrm {EW}~\ell \nu jj}~(7~\mathrm {TeV})= & {} 144 \pm 23~\mathrm {(stat)}~\pm 23~\mathrm {(exp)}~\pm 13~\mathrm {(th)~fb}, \\ \sigma ^\mathrm {fid}_{\mathrm {EW}~\ell \nu jj}~(8~\mathrm {TeV})= & {} 159 \pm 10~\mathrm {(stat)}~\pm 17~\mathrm {(exp)}~\pm 15~\mathrm {(th)~fb}, \end{aligned}$$

corresponding to a deviation of $<0.1\sigma ~(1.4\sigma )$ from the SM prediction of $144 \pm 11$ ($198 \pm 12$) fb at $\sqrt{s}=7~(8)$ $\text {TeV}$. The large sample size of the 8 $\text {TeV}$ measurement yields the smallest relative uncertainty of existing fiducial cross-section measurements of electroweak boson production in a VBF topology.

Differential cross sections of the $\sqrt{s}=8$ $\text {TeV}$ electroweak $Wjj$ production process are measured in a high-purity region with $M_{jj} >1$ $\text {TeV}$. The cross sections are measured as a function of dijet mass, dijet rapidity separation, dijet azimuthal angular separation, dijet $p_{\text {T}}$, leading-jet $p_{\text {T}}$, the number of jets within the dijet rapidity gap, and lepton and jet centralities. Additionally, differential cross sections are measured in various fiducial regions for the combined electroweak and strong $Wjj$ production with high dijet invariant mass. The differential measurements are integrated in each fiducial region to obtain additional fiducial cross-section measurements. The most inclusive region, where $M_{jj} >0.5$ $\text {TeV}$, $\Delta y(j_1,j_2) > 2$, $p_{\text {T}} ^{j_1} > 80$ $\text {GeV}$, and $p_{\text {T}} ^{j_2} > 60$ $\text {GeV}$, has a measured QCD+EW fiducial cross section at $\sqrt{s}=8$ $\text {TeV}$ of $\sigma ^\mathrm {fid}_{\mathrm {QCD+EW}~\ell \nu jj} =1700 \pm 110$ fb.

The region of increased purity for electroweak production of $Wjj$ ($M_{jj} >1$ $\text {TeV}$) is used to constrain dimension-six triple-gauge-boson operators motivated by an effective field theory. To improve the sensitivity to high-scale physics affecting the triple-gauge-boson vertex, events with leading-jet $p_{\text {T}} >600$ $\text {GeV}$ are also used to constrain CP-conserving and CP-violating operators in the HISZ scenario, both with and without a form-factor suppression. A 95% C.L. range of $[-0.13,0.09]$ is determined for $\lambda _V$ with a suppression scale of 4 $\text {TeV}$ and the other parameters set to their SM values. Limits are also set on the parameters of an effective field theory. The operator coefficient $c_{WWW}/\Lambda ^2$ is proportional to $\lambda _V$ and is constrained to $[-13, 9]/{\mathrm {\text {TeV}}}^2$ at 95% C.L. Constraints on CP-violating operators are similar to those on the CP-conserving operators.

## Figures and Tables

**Fig. 30:**
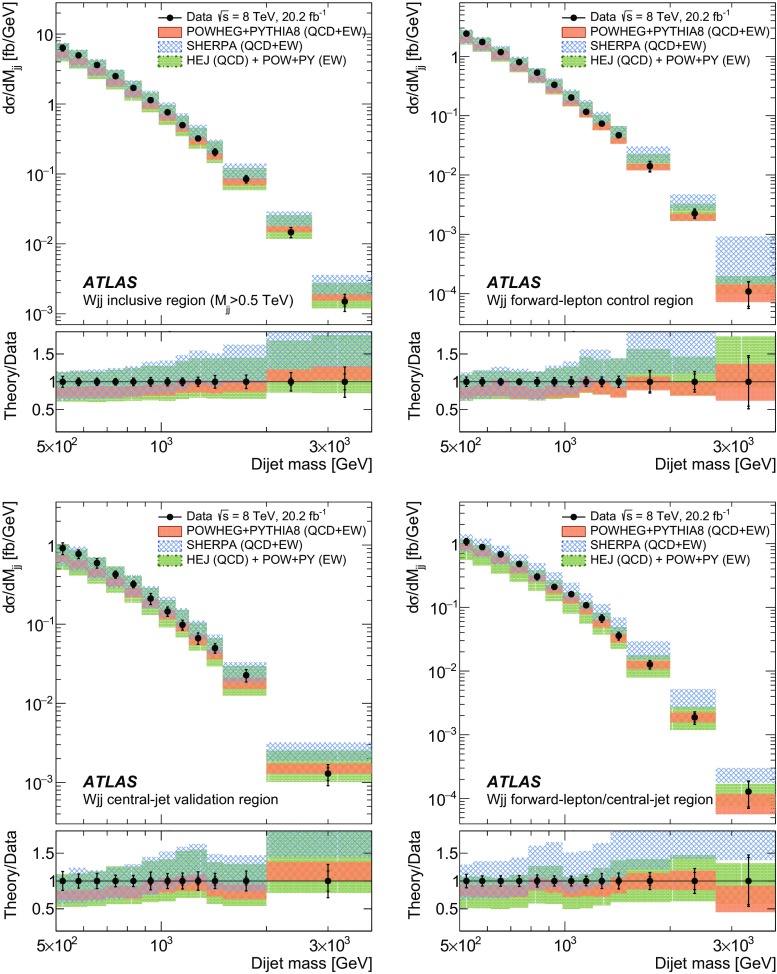
Unfolded differential $Wjj$ production cross sections as a function of dijet mass for the inclusive (*top left*), forward-lepton (*top right*), central-jet (*bottom left*), and forward-lepton/central-jet (*bottom right*) fiducial regions, which are enriched in strong $Wjj$ production. Both statistical (*inner bar*) and total (*outer bar*) measurement uncertainties are shown, as well as ratios of the theoretical predictions to the data (the *bottom panel* in each distribution)

**Fig. 31:**
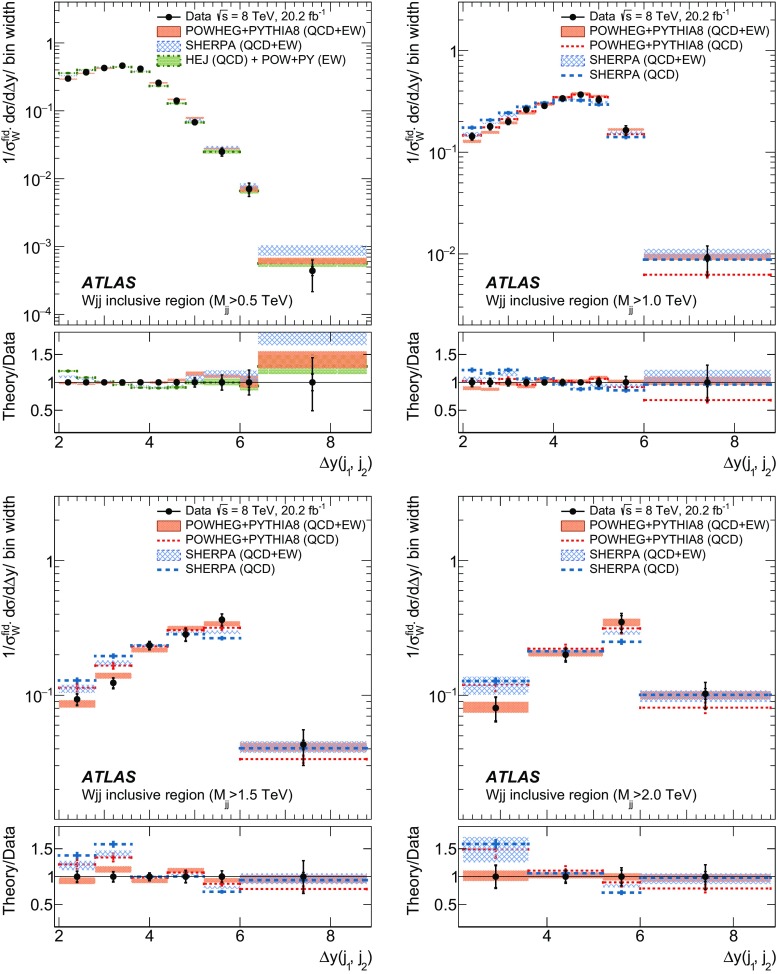
Unfolded normalized differential $Wjj$ production cross sections as a function of $\Delta y(j_1,j_2)$ in the inclusive fiducial region with four thresholds on the dijet invariant mass (0.5, 1.0, 1.5, and 2.0 $\text {TeV}$). Both statistical (*inner bar*) and total (*outer bar*) measurement uncertainties are shown, as well as ratios of the theoretical predictions to the data (the *bottom panel* in each distribution)

**Fig. 32:**
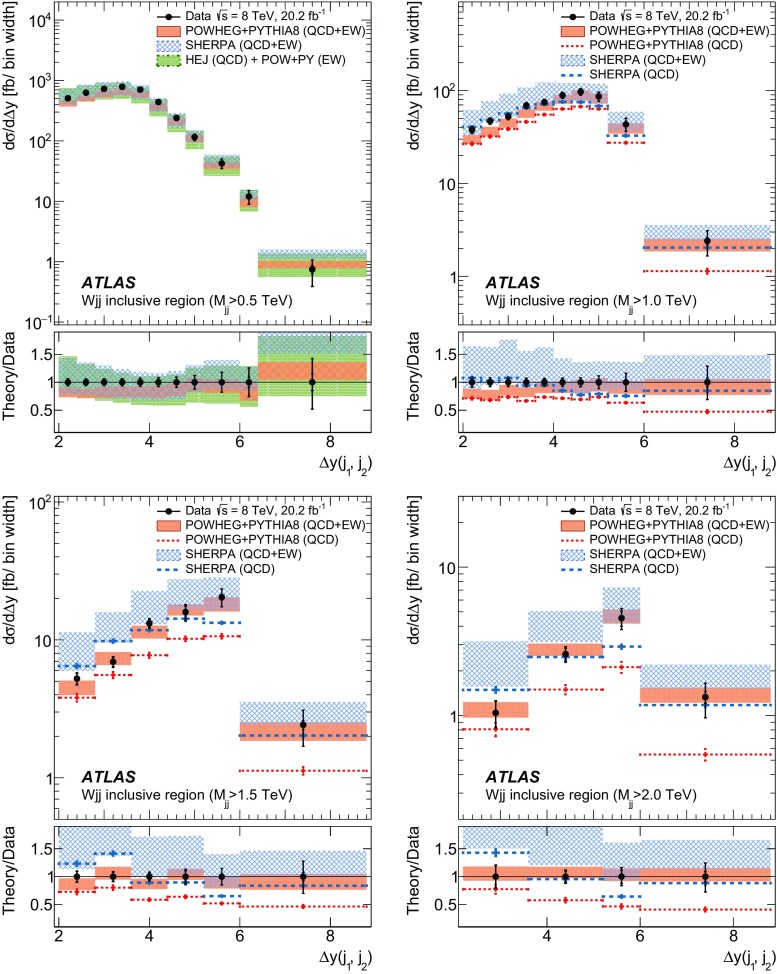
Unfolded absolute differential $Wjj$ production cross sections as a function of $\Delta y(j_1,j_2)$ for the inclusive fiducial region with progressively increasing dijet mass thresholds. Both statistical (*inner bar*) and total (*outer bar*) measurement uncertainties are shown, as well as ratios of the theoretical predictions to the data (the *bottom panel* in each distribution)

**Fig. 33:**
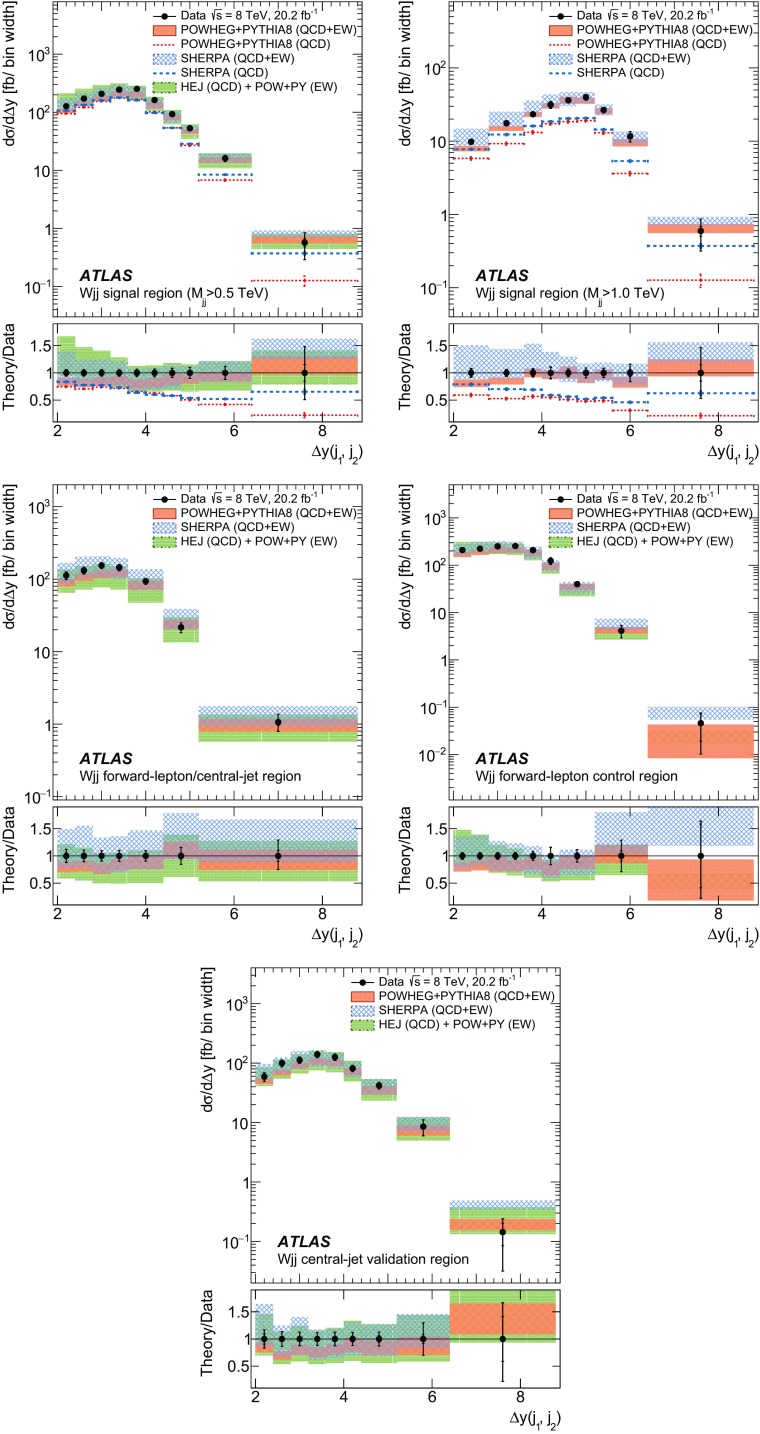
Differential $Wjj$ production cross sections as a function of $\Delta y(j_1,j_2)$ in the signal and high-mass signal fiducial regions, and in the forward-lepton, central-jet validation, and forward-lepton/central-jet fiducial regions. Both statistical (*inner bar*) and total (*outer bar*) measurement uncertainties are shown, as well as ratios of the theoretical predictions to the data (the *bottom panel* in each distribution)

**Fig. 34:**
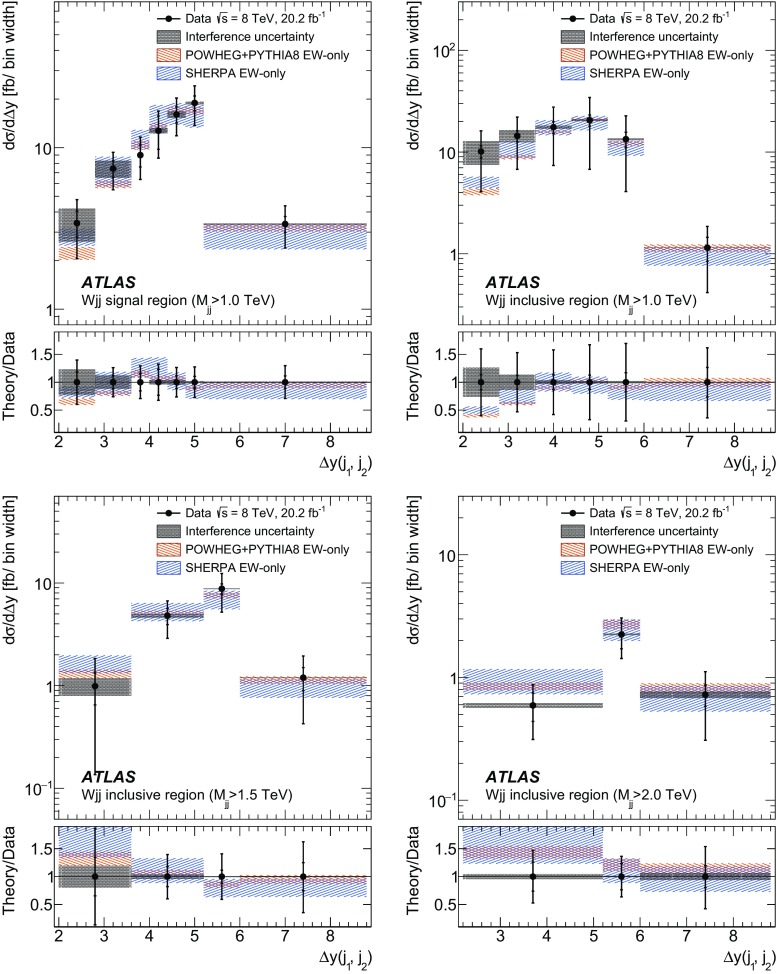
Differential electroweak $Wjj$ production cross sections as a function of $\Delta y(j_1,j_2)$ in the high-mass signal region and the inclusive fiducial region with three thresholds on the dijet invariant mass (1.0, 1.5, and 2.0). Both statistical (*inner bar*) and total (*outer bar*) measurement uncertainties are shown, as well as ratios of the theoretical predictions to the data (the *bottom panel* in each distribution)

**Fig. 35:**
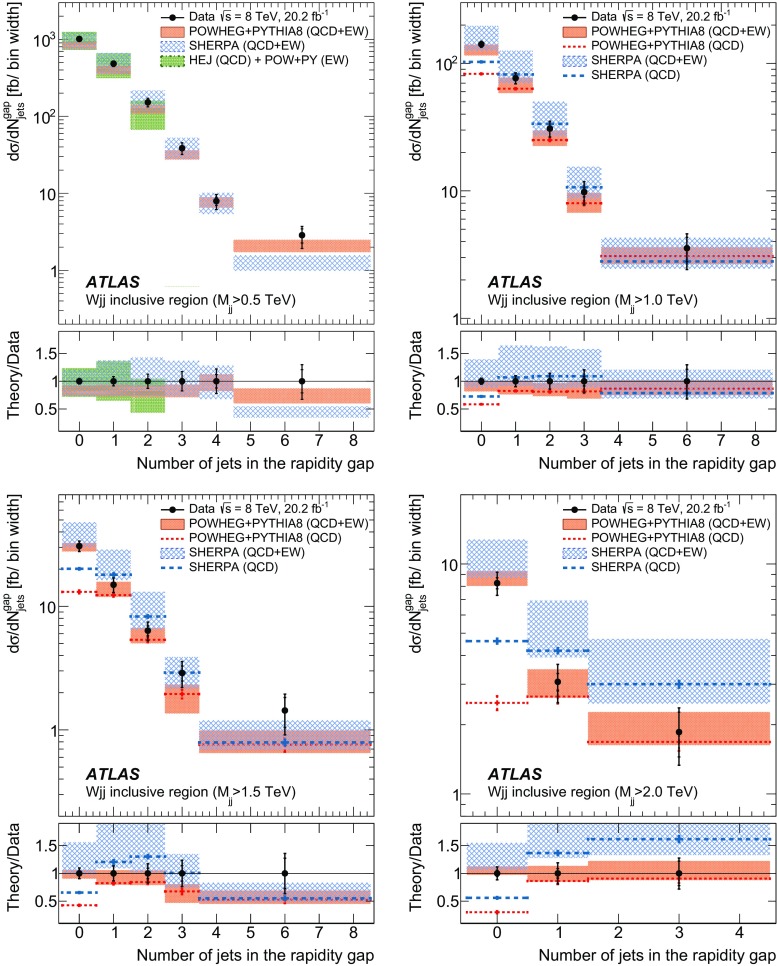
Differential $Wjj$ production cross sections as a function of the number of hard jets in the rapidity interval between the two leading jets in the inclusive fiducial region with four thresholds on the dijet invariant mass (0.5, 1.0, 1.5, and 2.0 $\text {TeV}$). Both statistical (*inner bar*) and total (*outer bar*) measurement uncertainties are shown, as well as ratios of the theoretical predictions to the data (the *bottom panel* in each distribution)

**Fig. 36:**
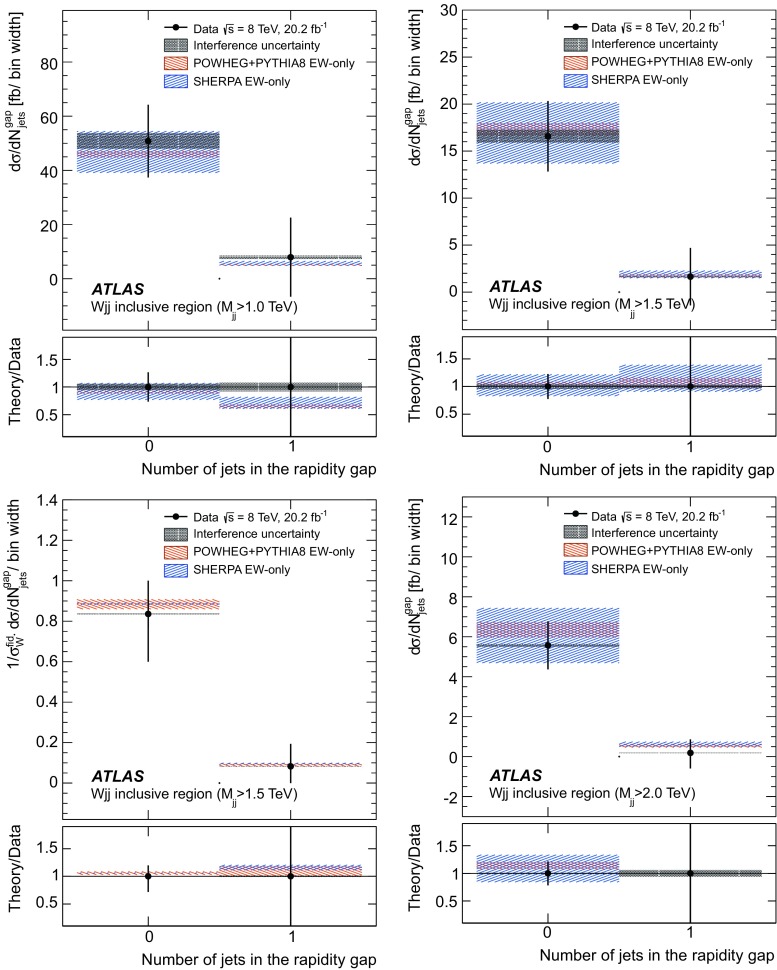
Differential electroweak $Wjj$ production cross sections as a function of the number of hard jets in the rapidity gap between the two leading jets in the inclusive fiducial region with $M_{jj} >1.0$ $\text {TeV}$ (*top left*), 1.5 $\text {TeV}$ (*top right* and *bottom left*), and 2.0 $\text {TeV}$(*bottom right*). The region with $M_{jj} > 1.5$ $\text {TeV}$, includes both absolute (*top right*) and normalized (*bottom left*) distributions. Both statistical (*inner bar*) and total (*outer bar*) measurement uncertainties are shown, as well as ratios of the theoretical predictions to the data (the *bottom panel* in each distribution)

**Fig. 37:**
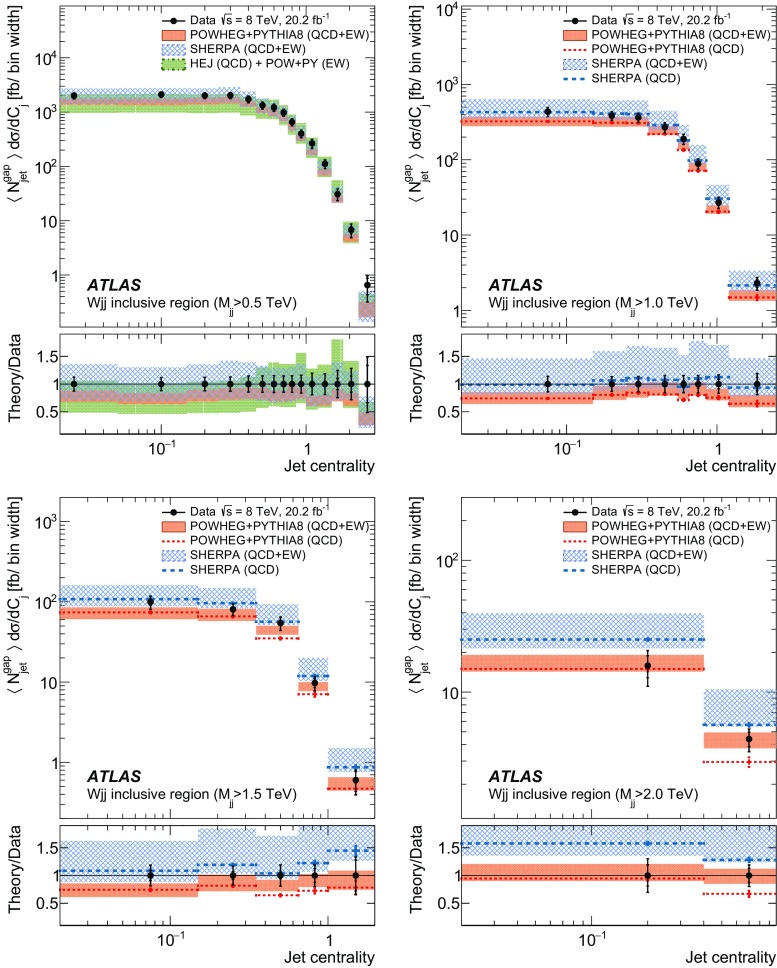
Differential $Wjj$ production cross sections as a function of jet centrality in the inclusive fiducial region with four thresholds on the dijet invariant mass (0.5, 1.0, 1.5, and 2.0 $\text {TeV}$). Both statistical (*inner bar*) and total (*outer bar*) measurement uncertainties are shown, as well as ratios of the theoretical predictions to the data (the *bottom panel* in each distribution)

**Fig. 38:**
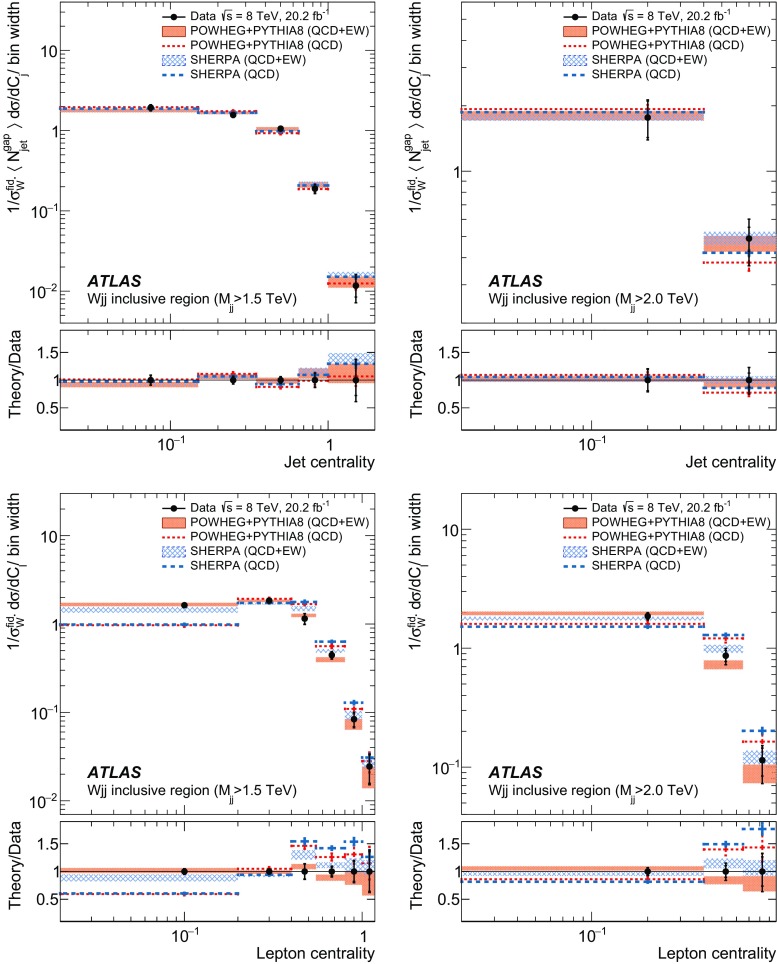
Unfolded normalized differential $Wjj$ production cross sections as a function of jet centrality (*top*) and lepton centrality (*bottom*) for the inclusive fiducial region with $M_{jj} > 1.5$ $\text {TeV}$ (*left*) and 2.0 $\text {TeV}$ (*right*). Both statistical (*inner bar*) and total (*outer bar*) measurement uncertainties are shown, as well as ratios of the theoretical predictions to the data (the *bottom panel* in each distribution)

**Fig. 39:**
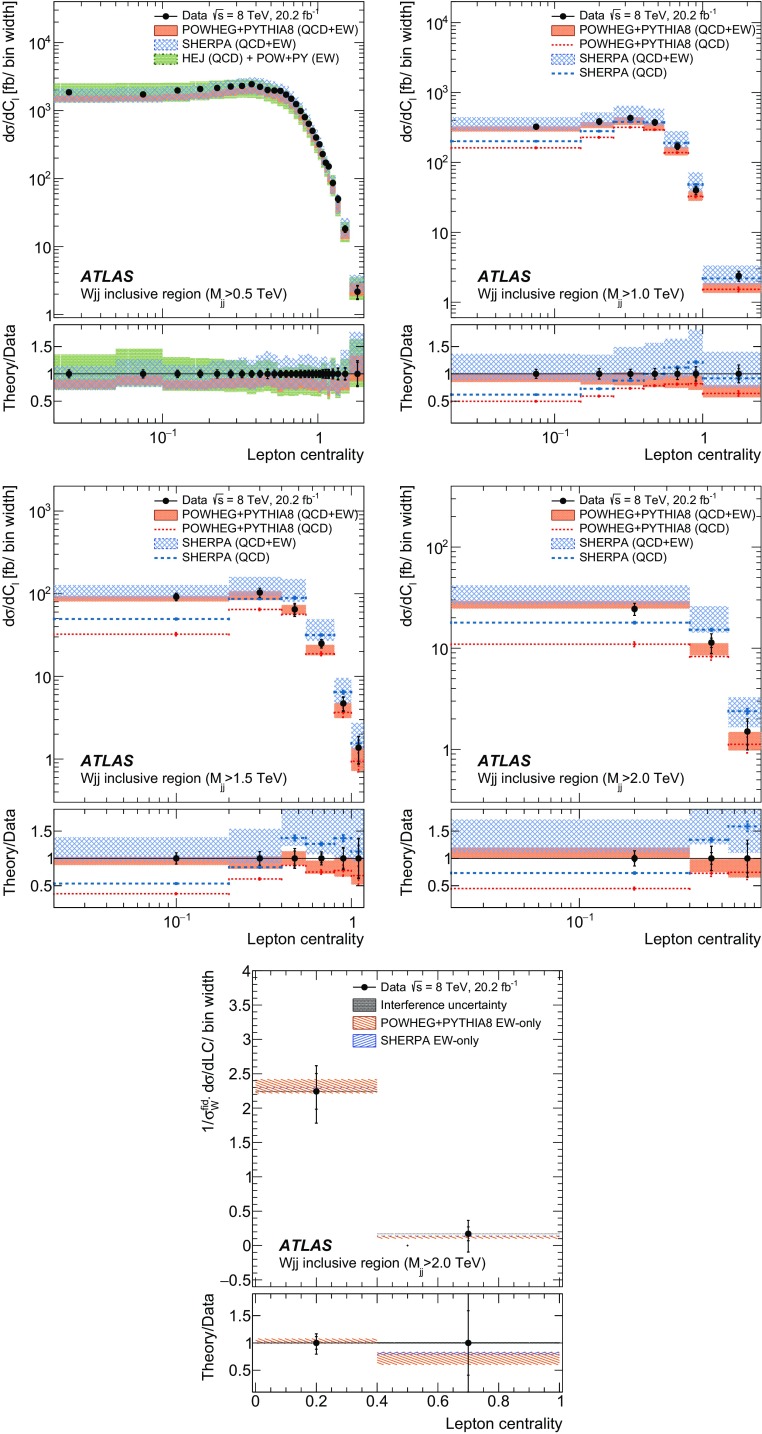
Unfolded differential $Wjj$ production cross sections as a function of lepton centrality in the inclusive fiducial region with four thresholds on the dijet invariant mass (0.5, 1.0, 1.5, and 2.0 $\text {TeV}$). The *bottom plot* shows the normalized distribution for $M_{jj} > 2.0$ $\text {TeV}$. Both statistical (*inner bar*) and total (*outer bar*) measurement uncertainties are shown, as well as ratios of the theoretical predictions to the data (the *bottom panel* in each distribution)

**Fig. 40:**
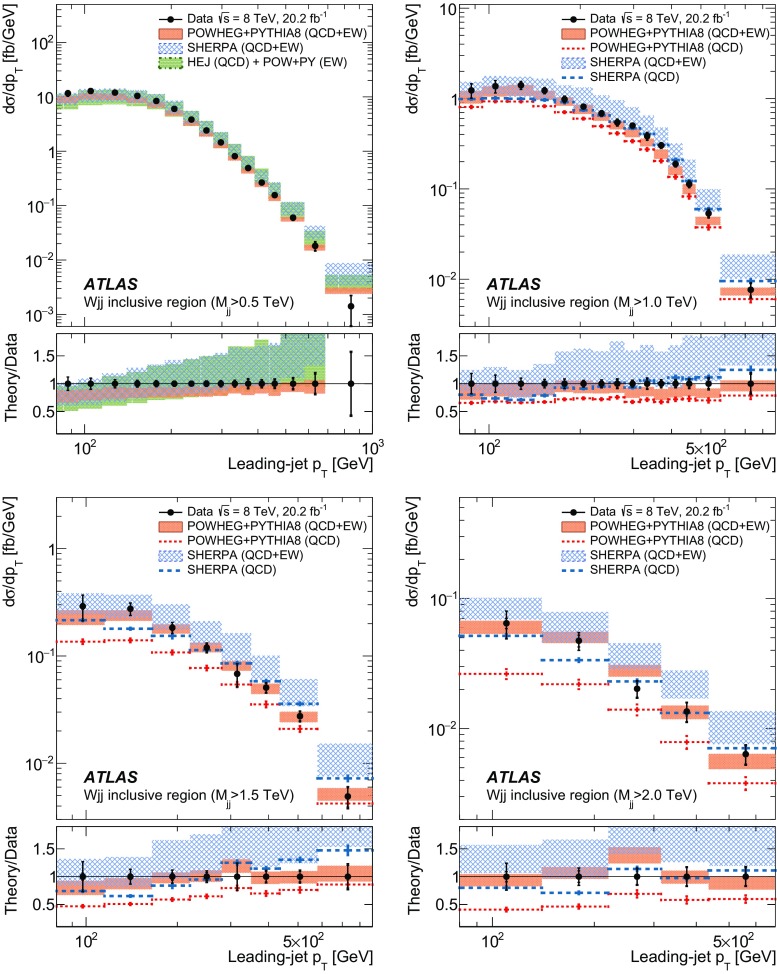
Unfolded absolute differential $Wjj$ production cross sections as a function of leading-jet $p_{\text {T}}$ for the inclusive fiducial region when the dijet invariant mass threshold is progressively raised in 500 $\text {GeV}$ increments from 0.5 $\text {TeV}$ (*top left*) to 2.0 $\text {TeV}$ (*bottom right*). Both statistical (*inner bar*) and total (*outer bar*) measurement uncertainties are shown, as well as ratios of the theoretical predictions to the data (the *bottom panel* in each distribution)

**Fig. 41:**
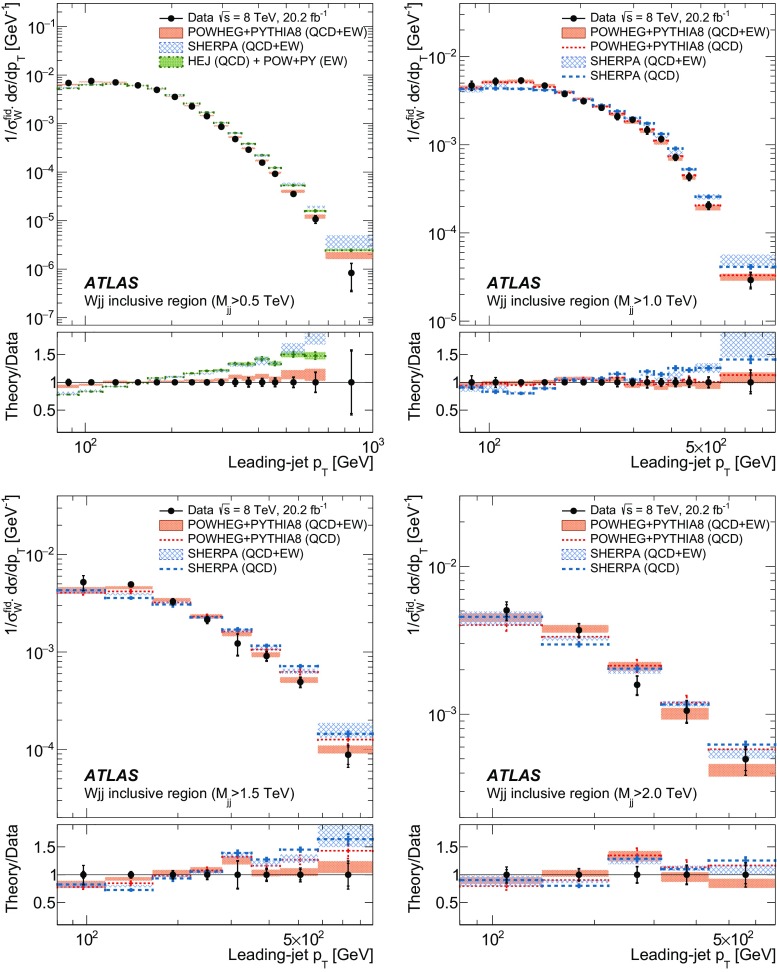
Unfolded normalized differential $Wjj$ production cross sections as a function of the leading-jet $p_{\text {T}}$ in the inclusive fiducial region with four thresholds on the dijet invariant mass (0.5, 1.0, 1.5, and 2.0 $\text {TeV}$). Both statistical (*inner bar*) and total (*outer bar*) measurement uncertainties are shown, as well as ratios of the theoretical predictions to the data (the *bottom panel* in each distribution)

**Fig. 42:**
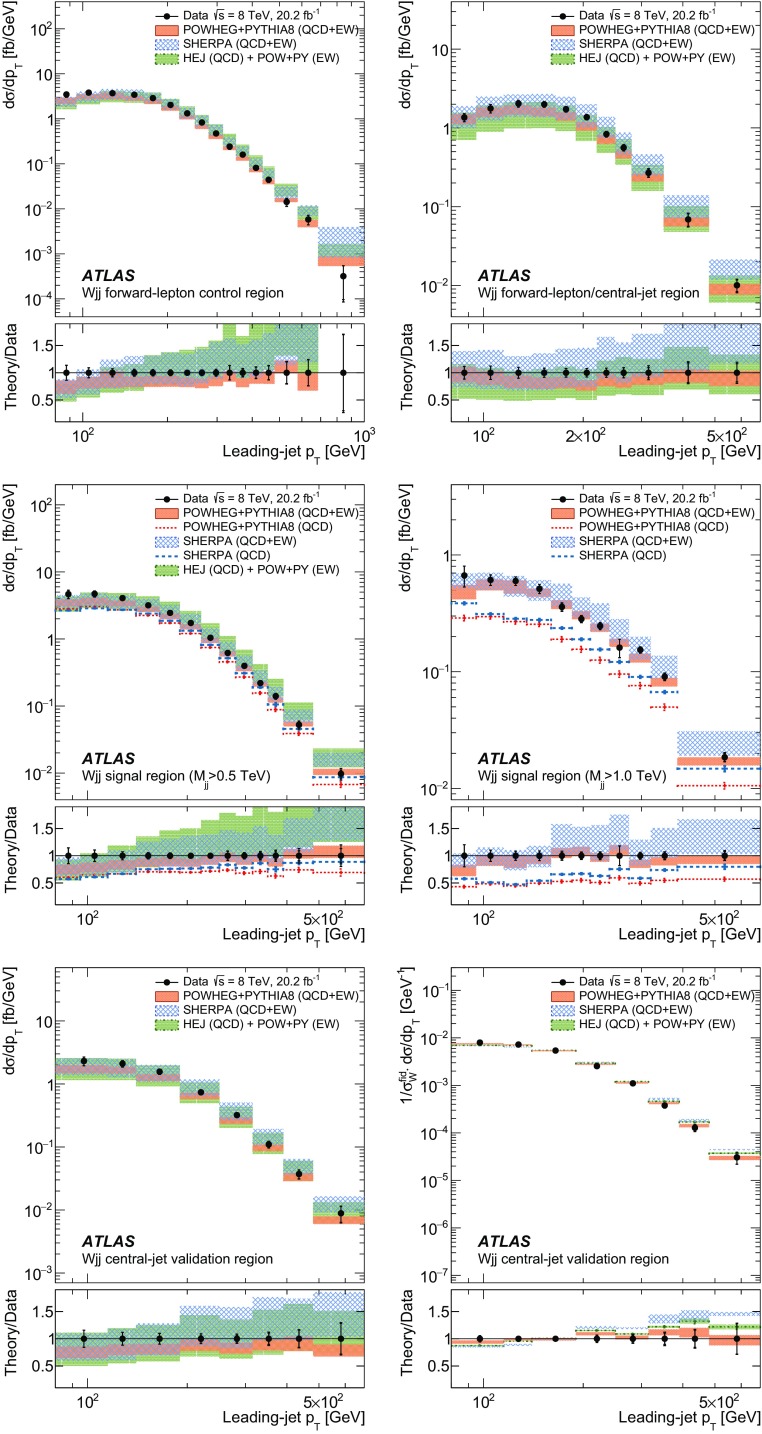
Unfolded absolute differential $Wjj$ production cross sections as a function of leading-jet $p_{\text {T}}$ for the forward-lepton control region (*top left*), forward-lepton/central-jet fiducial region (*top right*), the signal regions with $M_{jj} >0.5$ TeV (*middle left*) and 1.0 $\text {TeV}$(*middle right*), and the central-jet validation region (*bottom*). The absolute (*left*) and normalized (*right*) distributions are shown in the central-jet region. Both statistical (*inner bar*) and total (*outer bar*) measurement uncertainties are shown, as well as ratios of the theoretical predictions to the data (the *bottom panel* in each distribution)

**Fig. 43:**
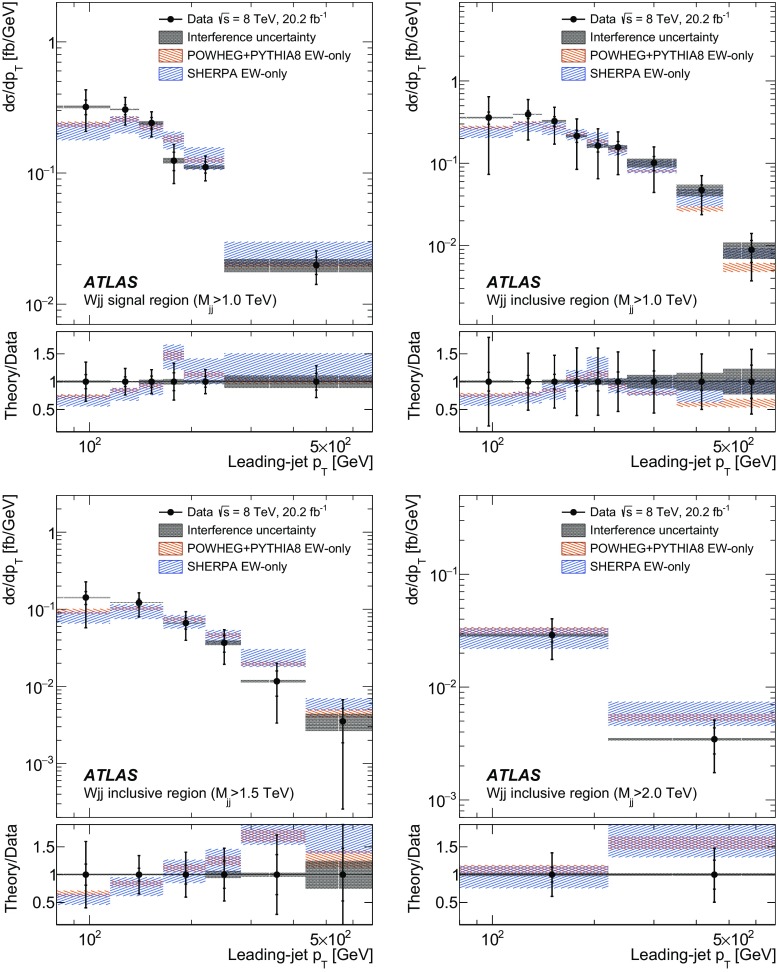
Differential electroweak $Wjj$ production cross sections as a function of the leading-jet $p_{\text {T}}$ in the high-mass signal region and the inclusive fiducial region with three thresholds on the dijet invariant mass (1.0, 1.5, and 2.0 $\text {TeV}$). Both statistical (*inner bar*) and total (*outer bar*) measurement uncertainties are shown, as well as ratios of the theoretical predictions to the data (the *bottom panel* in each distribution)

**Fig. 44:**
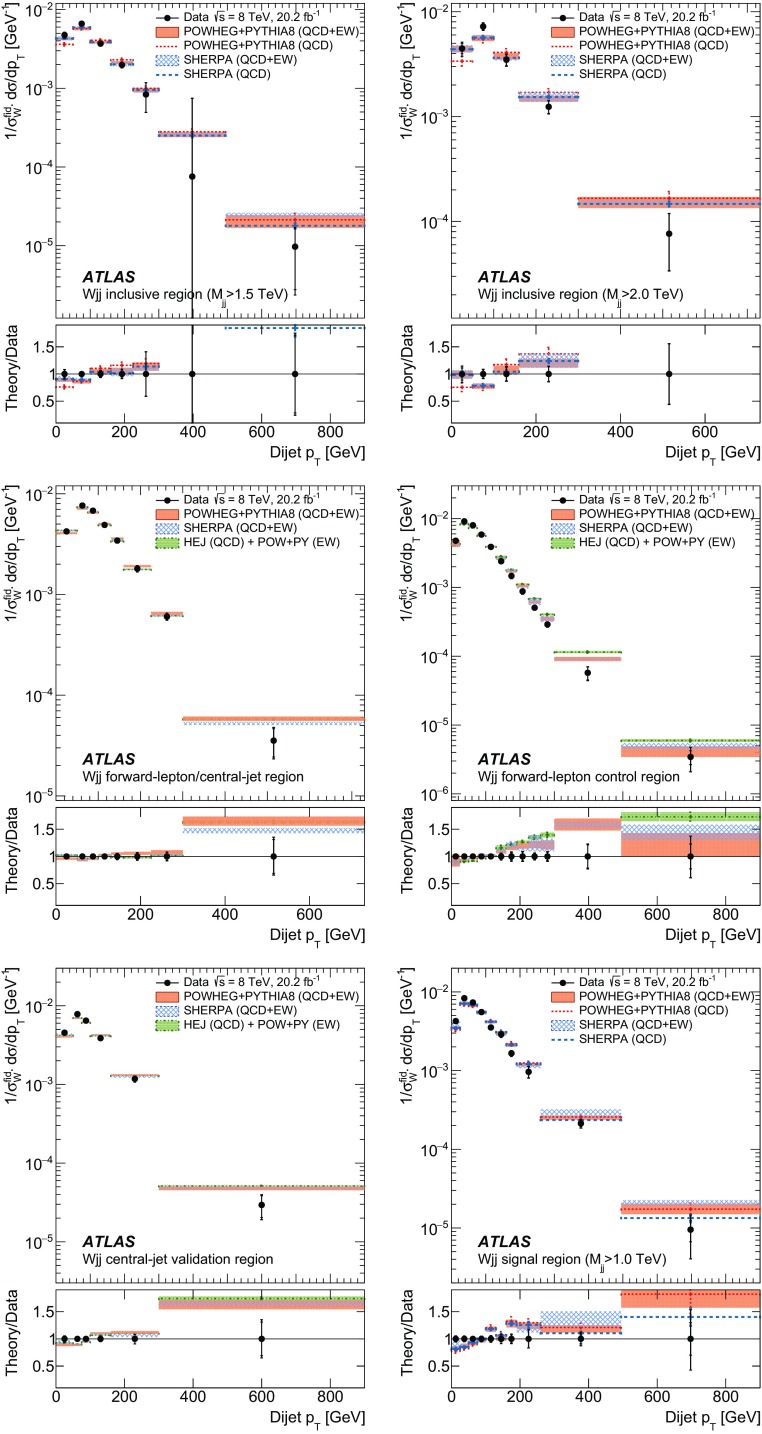
Unfolded normalized differential $Wjj$ production cross sections as a function of dijet $p_{\text {T}}$ in the inclusive (*top*), forward-lepton/central-jet (*middle left*), forward-lepton (*middle right*), central-jet (*bottom left*), and high-mass signal (*bottom right*) fiducial regions. The inclusive regions show the distributions for $M_{jj}$ thresholds of 1.5 $\text {TeV}$ (*left*) and 2.0 $\text {TeV}$ (*right*). Both statistical (*inner bar*) and total (*outer bar*) measurement uncertainties are shown, as well as ratios of the theoretical predictions to the data (the *bottom panel* in each distribution)

**Fig. 45:**
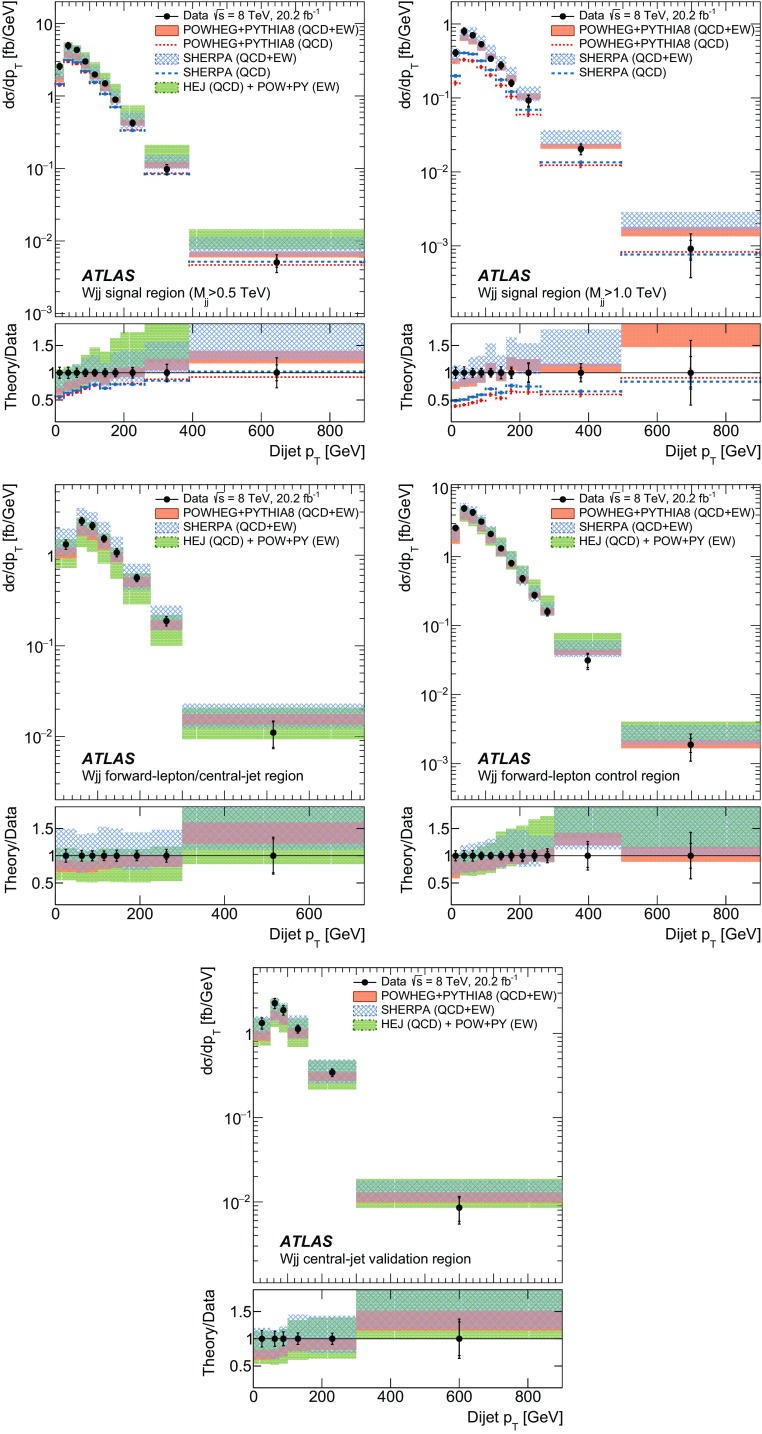
Differential $Wjj$ production cross sections as a function of dijet $p_{\text {T}}$ in the signal, high-mass signal, forward-lepton/central-jet, and forward-lepton regions fiducial regions. Both statistical (*inner bar*) and total (*outer bar*) measurement uncertainties are shown, as well as ratios of the theoretical predictions to the data (the *bottom panel* in each distribution)

**Fig. 46:**
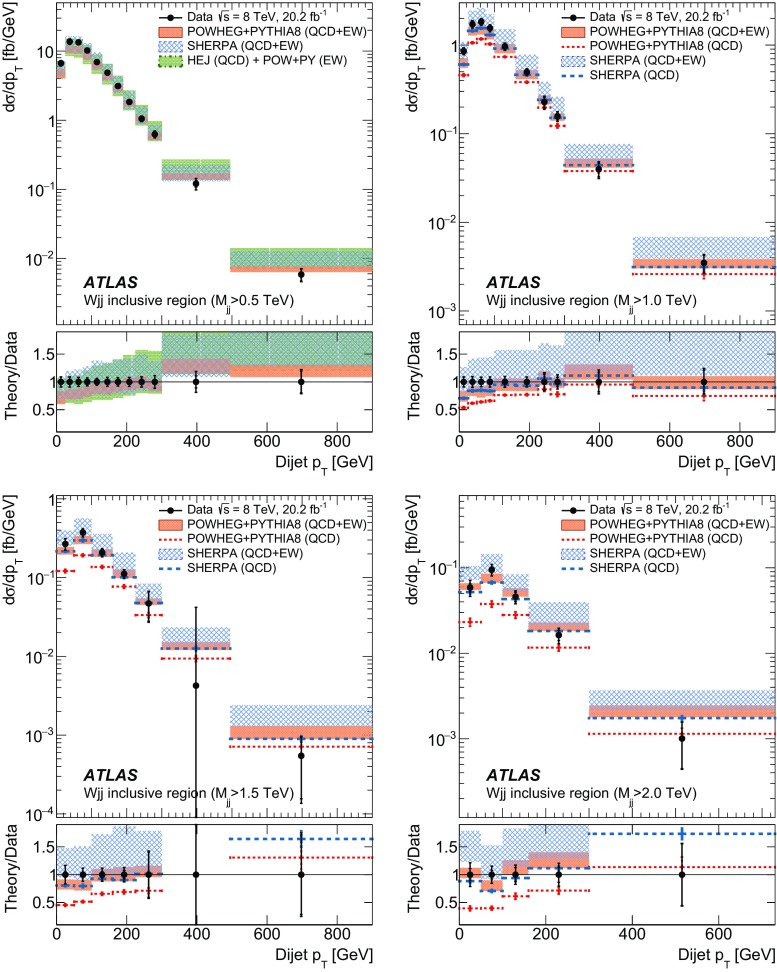
Differential $Wjj$ production cross sections as a function of dijet $p_{\text {T}}$ in the inclusive fiducial region with four thresholds on the dijet invariant mass (0.5, 1.0, 1.5, and 2.0 $\text {TeV}$). Both statistical (*inner bar*) and total (*outer bar*) measurement uncertainties are shown, as well as ratios of the theoretical predictions to the data (the *bottom panel* in each distribution)

**Fig. 47:**
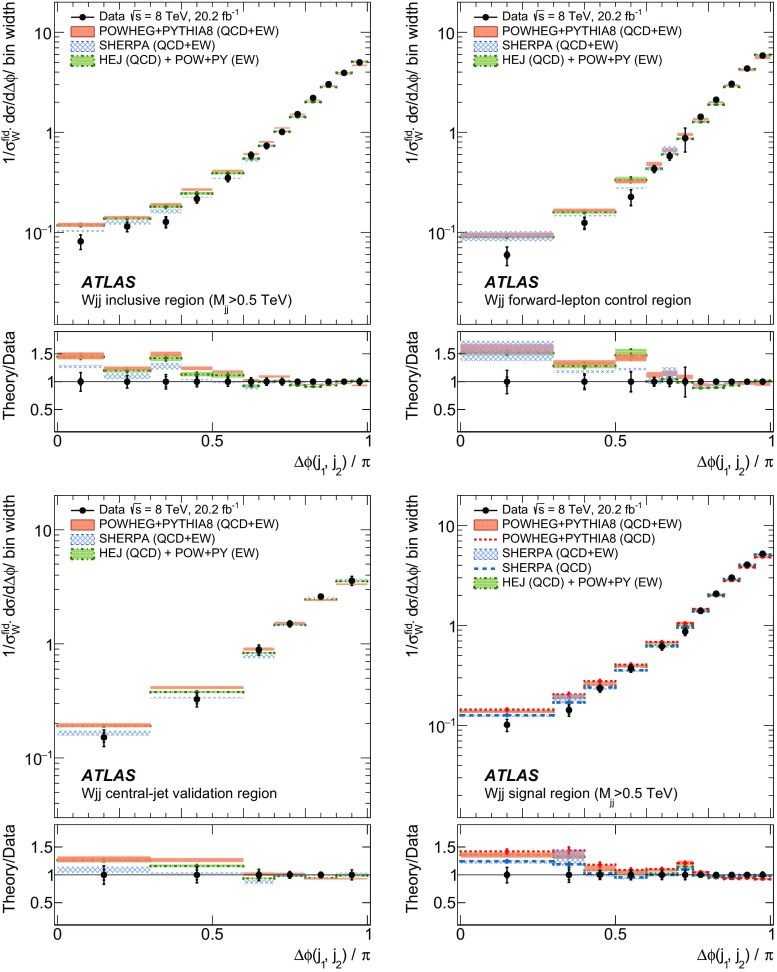
Unfolded normalized differential production cross sections as a function of $\Delta \phi (j_1,j_2)$ for the inclusive, forward-lepton control, central-jet validation, and signal fiducial regions. Both statistical (*inner bar*) and total (*outer bar*) measurement uncertainties are shown, as well as ratios of the theoretical predictions to the data (the *bottom panel* in each distribution)

**Fig. 48:**
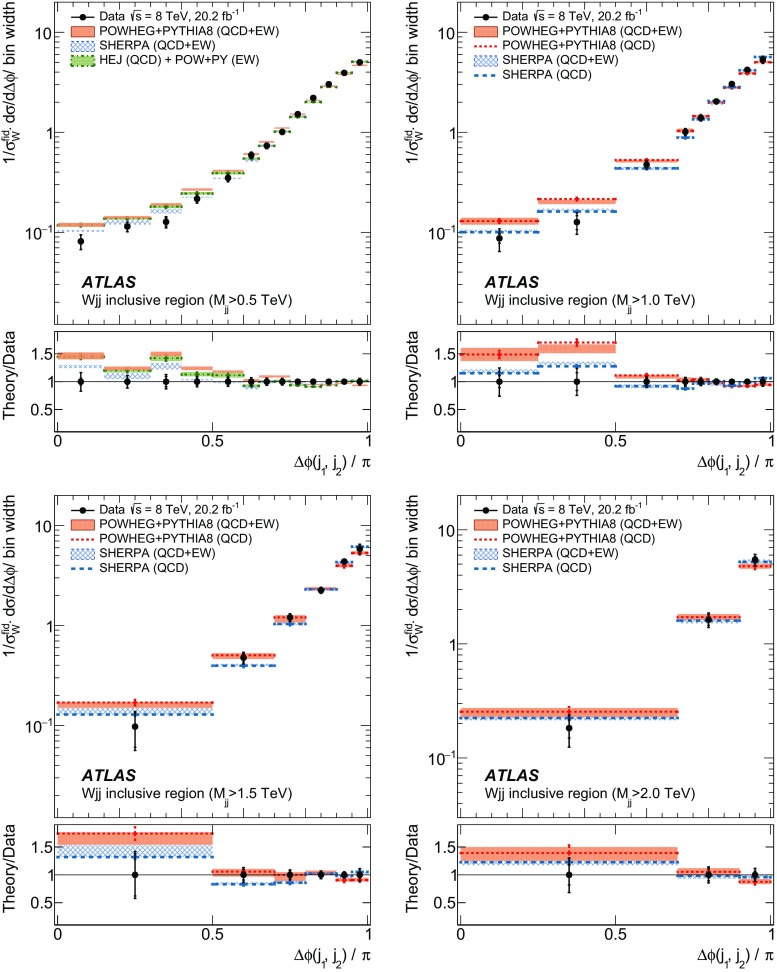
Unfolded normalized differential $Wjj$ production cross sections as a function of $\Delta \phi (j_1,j_2)$ in the inclusive fiducial region with four thresholds on the dijet invariant mass (0.5, 1.0, 1.5, and 2.0 $\text {TeV}$). Both statistical (*inner bar*) and total (*outer bar*) measurement uncertainties are shown, as well as ratios of the theoretical predictions to the data (the *bottom panel* in each distribution)

**Fig. 49:**
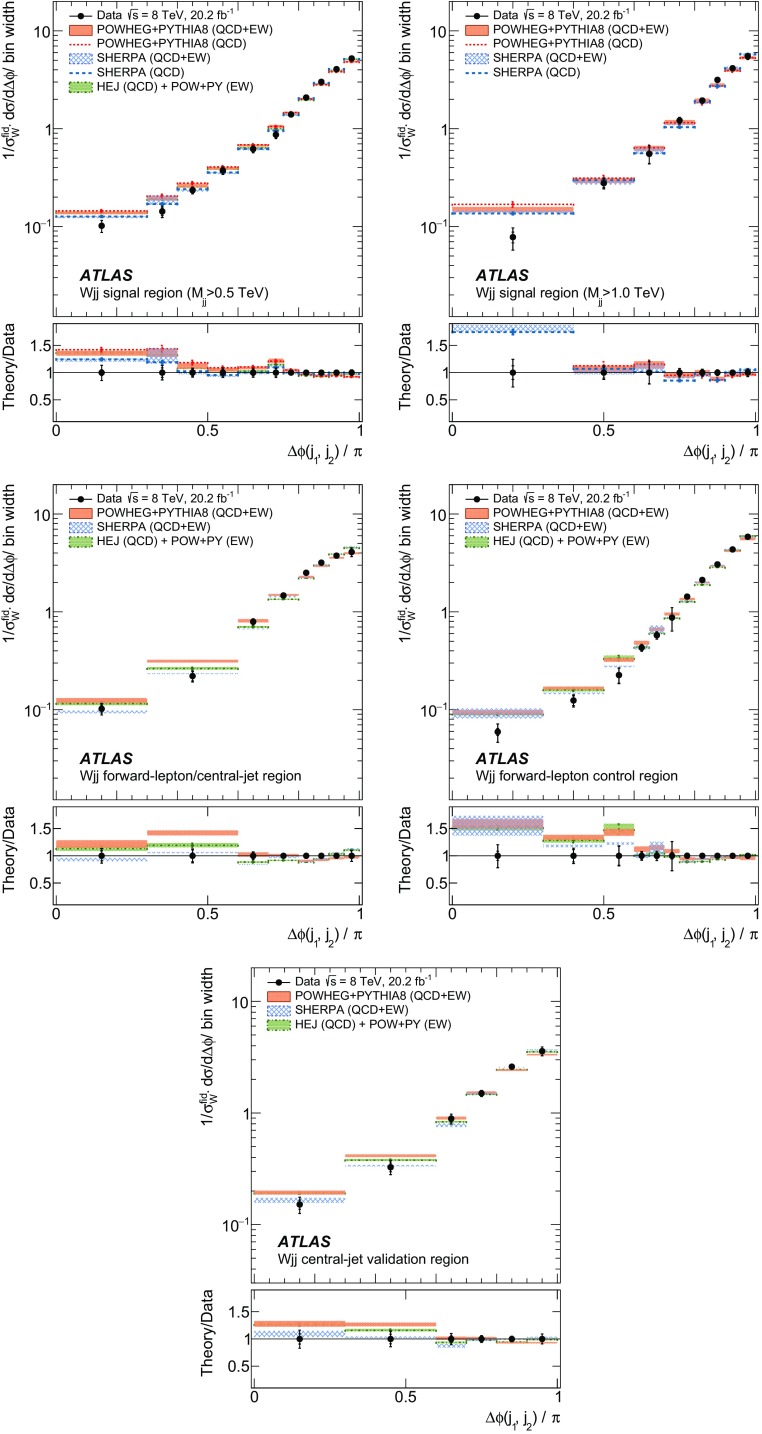
Unfolded normalized differential $Wjj$ production cross sections as a function of $\Delta \phi (j_1,j_2)$ in the signal, high-mass signal, forward-lepton/central-jet, forward-lepton, and central-jet fiducial regions. Both statistical (*inner bar*) and total (*outer bar*) measurement uncertainties are shown, as well as ratios of the theoretical predictions to the data (the *bottom panel* in each distribution)

**Fig. 50:**
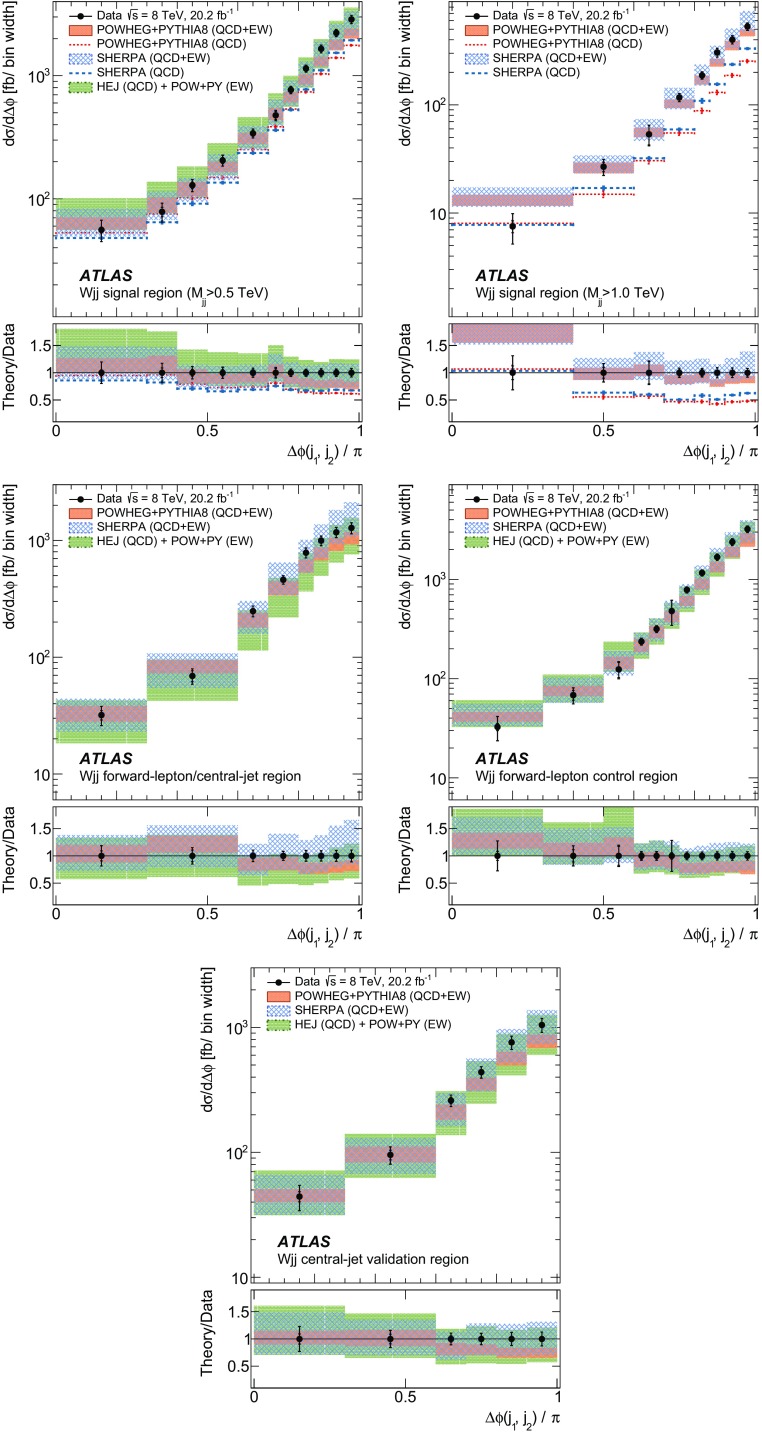
Differential $Wjj$ production cross sections as a function of $\Delta \phi (j_1,j_2)$ in the signal, high-mass signal, forward-lepton/central-jet, forward-lepton, and central-jet fiducial regions. Both statistical (*inner bar*) and total (*outer bar*) measurement uncertainties are shown, as well as ratios of the theoretical predictions to the data (the *bottom panel* in each distribution)

**Fig. 51:**
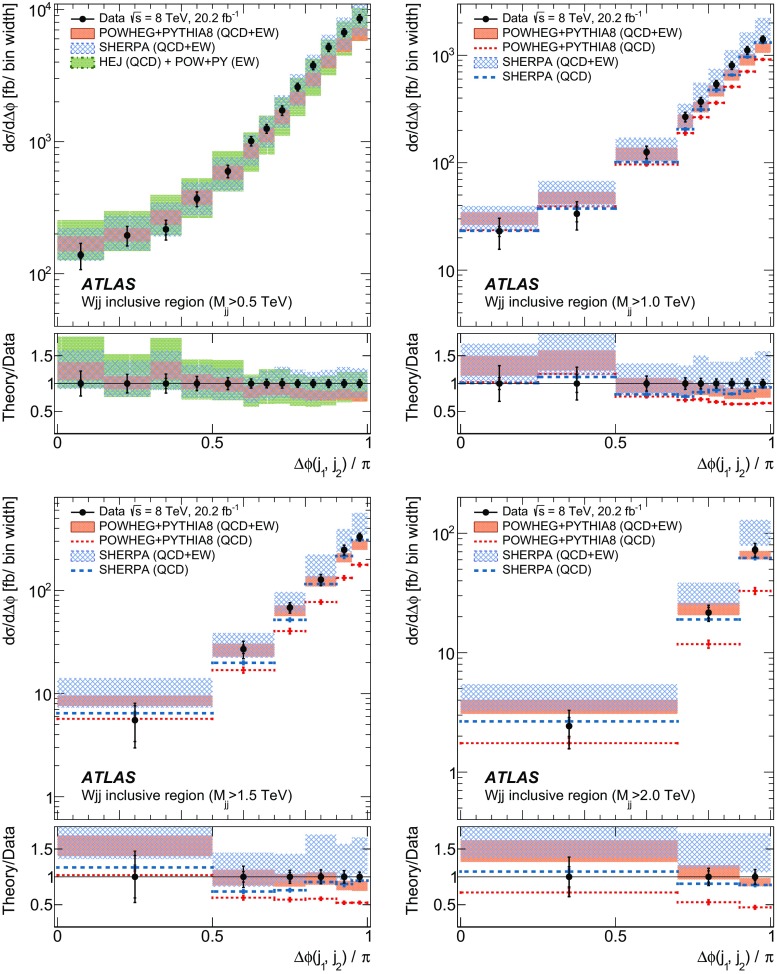
Differential $Wjj$ production cross sections as a function of $\Delta \phi (j_1,j_2)$ in the inclusive fiducial region with four thresholds on the dijet invariant mass (0.5, 1.0, 1.5, and 2.0 $\text {TeV}$). Both statistical (*inner bar*) and total (*outer bar*) measurement uncertainties are shown, as well as ratios of the theoretical predictions to the data (the bottom panel in each distribution)

**Fig. 52:**
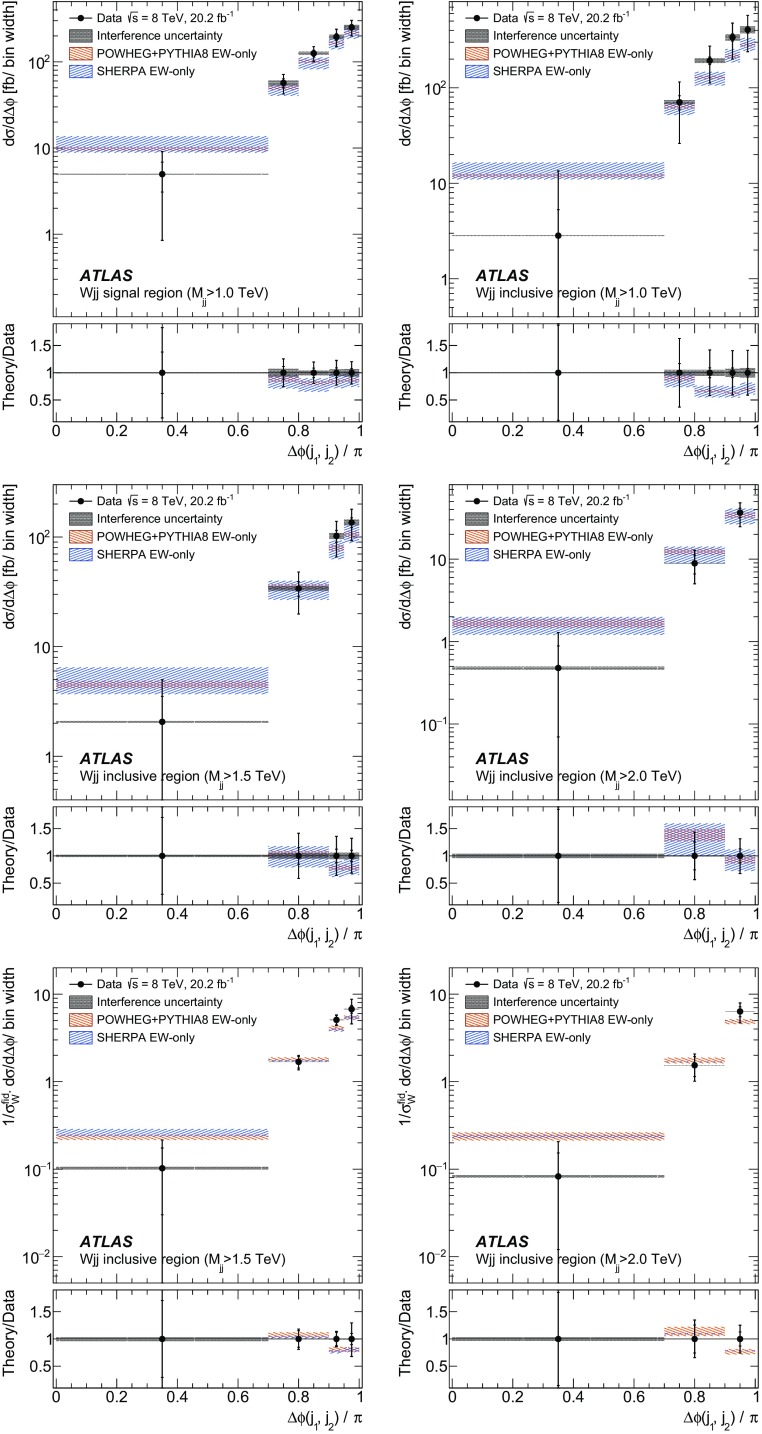
Differential electroweak $Wjj$ production cross sections as a function of $\Delta \phi (j_1,j_2)$ in the high-mass signal region and the inclusive fiducial region with three thresholds on the dijet invariant mass (1.0, 1.5, and 2.0 $\text {TeV}$). The *bottom* two distributions are normalized, the rest are absolute. Both statistical (*inner bar*) and total (*outer bar*) measurement uncertainties are shown, as well as ratios of the theoretical predictions to the data (the *bottom panel* in each distribution)

## A Appendix

This section includes normalized and absolute differential QCD+EW and EW $Wjj$ production cross-section measurements not directly discussed in the main text. (Figs. 30, 31, 32, 33, 34, 35, 36, 37, 38, 39, 40, 41, 42, 43, 44, 45, 46, 47, 48, 49, 50, 51, 52). The complete set of measured differential spectra is available in hepdata [77].

## Footnotes

1 ATLAS uses a right-handed coordinate system with its origin at the nominal interaction point in the centre of the detector and the *z*-axis along the beam pipe. The *x*-axis points from the interaction point to the centre of the LHC ring, and the *y*-axis points upward. Cylindrical coordinates $(r,\phi )$ are used in the transverse plane, $\phi$ being the azimuthal angle around the *z*-axis. The pseudorapidity is defined in terms of the polar angle $\theta$ as $\eta =-\ln \tan (\theta /2)$. The rapidity is defined as $y=0.5\ln [(E+p_z)/(E-p_z)]$, where *E* and $p_z$ are the energy and longitudinal momentum, respectively. Momentum in the transverse plane is denoted by $p_{\text {T}}$.
